# Triazole‐Linked Pyrimidine Nucleoside Dimers: Design, Synthesis, and Applications in Medicinal and Nucleic Acid Chemistry. Part IV

**DOI:** 10.1111/cbdd.70407

**Published:** 2026-10-04

**Authors:** Dagmara Baraniak, Dariusz Wawrzyniak, Daniel Baranowski

**Affiliations:** ^1^ Institute of Bioorganic Chemistry, Polish Academy of Sciences Poznań Poland

**Keywords:** 1,2,3‐triazol, 4′‐ethynyl‐5′‐deoxy‐5′‐nor‐nucleoside, AddU, AFddU, anticancer activity, AZT, click chemistry, CuAAC, nucleoside dimers, SAR

## Abstract

A new class of pyrimidine nucleoside dimers featuring an ultrashort 1,4‐disubstituted 1,2,3‐triazole linker was designed and synthesized to explore the biological potential of structurally robust nucleoside analogues as anticancer agents. The target compounds were efficiently obtained via Cu(I)‐catalyzed azide‐alkyne cycloaddition (CuAAC) from appropriately functionalized 3′‐azido‐ and 4′‐ethynyl‐5′‐deoxy‐5′‐nor nucleoside precursors and fully characterized by multinuclear 1D/2D NMR spectroscopy and ESI‐MS. Their antiproliferative activity was evaluated against a panel of human cancer cell lines comprising cervical (HeLa), breast (T47D), ovarian (SK‐OV‐3), and glioblastoma (U‐138 MG and U‐251 MG) cells, with MRC‐5 human fibroblasts serving as the control. Several dimers displayed pronounced and cell line‐selective antiproliferative activity while exhibiting limited toxicity toward normal cells. Dimers **4**, **9**, **11**, and **17** demonstrated the highest activity against HeLa cells, whereas Dimers **7** and **15** were the most potent against U‐251 MG glioblastoma cells, with activities comparable to or exceeding that of 5‐fluoro‐2′‐deoxyuridine (5FdU). Mechanistic studies performed on the most active glioblastoma‐directed dimers revealed a concentration‐dependent reduction in clonogenic survival and cell migration, accompanied by increased cell death and pronounced S‐phase cell cycle arrest, closely resembling the effect of the reference compound 5FdU. In contrast, only a modest and transient increase in intracellular reactive oxygen species (ROS) was detected, indicating that oxidative stress is unlikely to represent the primary mechanism responsible for their antiproliferative activity. Preliminary structure–activity relationship analysis demonstrated that both the nature of the nucleobase and the rigid triazole‐linked dimeric architecture substantially influence biological activity and selectivity. Moreover, the synthesized dimers exhibited excellent chemical stability in cell culture medium and human serum, highlighting their exceptional resistance to degradation. Taken together, these findings identify triazole‐linked pyrimidine nucleoside dimers as highly stable bioactive scaffolds that inhibit glioblastoma cell proliferation through disruption of DNA synthesis rather than oxidative stress and represent promising candidates for further development as anticancer agents or durable nucleic acid‐targeting molecular tools.

## Graphical abstract

A series of 18 pyrimidine nucleoside dimers analogues with a short 1,4‐disubstituted 1,2,3‐triazole linkage were synthesized using a click chemistry approach (CuAAC). Analysis of the obtained products was conducted utilizing 1D/2D NMR spectroscopy (^1^H, ^13^C, ^19^F, ^15^N, and ^29^Si), mass spectrometry (ESI‐MS), and high‐performance liquid chromatography (HPLC). The antiproliferative activity was evaluated against a panel of human cancer cell lines comprising cervical (HeLa), breast (T47D), ovarian (SK‐OV‐3), and glioblastoma (U‐138 MG and U‐251 MG) cells. Dimers **4**, **9**, **11**, and **17** demonstrated the highest activity against HeLa cells, whereas Dimers **7** and **15** were the most potent against U‐251 MG glioblastoma cells.

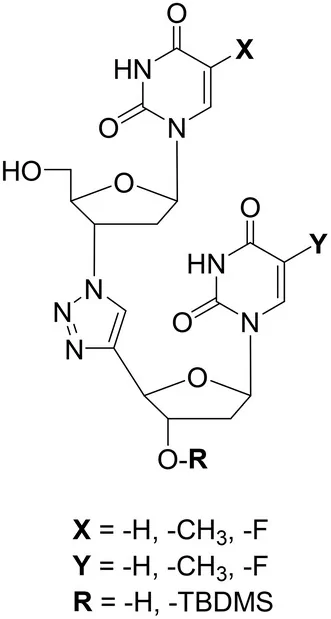

## 1 Introduction

Cancer represents a heterogeneous group of diseases characterized by dysregulated cell proliferation, impaired programmed cell death, and the capacity for local invasion and distant metastasis. Malignant transformation arises primarily from the accumulation of genetic and epigenetic alterations induced by environmental exposure or inherited predisposition, ultimately leading to uncontrolled tumor growth (WHO 2020; Hanahan and Weinberg 2011; Madkour 2019). With the exception of hematological malignancies, these processes typically result in the formation of solid tumors. According to the World Health Organization, cancer remains the second leading cause of death worldwide, surpassed only by cardiovascular diseases, with approximately 10 million cancer‐related deaths reported in 2020 alone. Despite significant advances in diagnosis and treatment, the global cancer burden continues to rise, driven in part by lifestyle‐related exposure to carcinogens and aging populations. Consequently, there is an urgent and sustained need for the development of new anticancer therapeutics that combine high efficacy with improved selectivity and reduced systemic toxicity. In this context, chemotherapy remains a cornerstone of cancer treatment, particularly for advanced and metastatic disease. However, the clinical utility of many established chemotherapeutic agents is limited by narrow therapeutic windows, dose‐limiting toxicities, and the emergence of drug resistance. These challenges underscore the importance of medicinal chemistry approaches aimed at designing novel small‐molecule and nucleoside‐based anticancer agents with optimized structure–activity relationships and favorable pharmacological profiles (Kumar et al. 2025).

Nucleosides analogues (NAs) are an important group of compounds because they were the first chemotherapeutic agents introduced for the treatment of cancer and generally due to their wide range of applications in medicine (Galmarini et al. 2002; Hruba et al. 2023). These agents behave as antimetabolites, compete with physiological nucleosides, interact with a large number of intracellular targets involved in nucleoside metabolism and signaling to induce cytotoxicity (Brunton et al. 2006). Each of these compounds has unique drug–target interactions that help explain their differences in activity in various diseases, but also may cause toxic effects by inhibiting other enzymes. Among them, fluorinated nucleobases and nucleosides analogues showed important biological properties, including 5‐fluorouracil (FUra, 5FU) and 5‐fluoro‐2′‐deoxyuridine (floxuridine, 5FdU), that represent anticancer drugs with a wide spectrum of use in clinical practice (Parker 2009). Both of the above‐mentioned medicaments were discovered and came into medical use through FDA approval about 60 years ago (Heidelberger et al. 1958). Their mechanism of action is well known, and they have been proven to work in several ways inside the cell (Longley et al. 2003; de Clercq 2023). The pivotal role is related to the metabolic transformation into their active form, which is 5‐fluoro‐2′‐deoxyuridine monophosphate (5FdUMP), that is an irreversible inhibitor of thymidylate synthase enzyme (TS) (Langenbach et al. 1972; Danenberg 1977). Blocking of the key enzyme responsible for thymidine biosynthesis influences a crucial stage in life, which consequently leads to the termination of DNA synthesis. As a result, cell replication and division are blocked, leading to cell death. The cytostatic activity of the described fluorinated nucleoside analogues results from interference with the principle of the origin of life by making impossible C−C linkage creation, namely the dUMP → dTMP reaction (de Clercq 2023). The second anticancer mechanism of fluoropyrimidine drugs consists of their further phosphorylation to 5‐fluoro‐2′‐deoxyuridine triphosphates (5FdUTP), which can be incorporated into cellular DNA. Such 5‐fluorouracil structural element causes a decrease in DNA stability and functionality, which leads to the inhibition of cell division. It is worth mentioning that the efficiency of the 5FdU transformation into 5FdUMP in the cell is hampered by a simultaneous cleavage of the glycosyl bond of the nucleoside by thymidine phosphorylase that releases the nucleobase 5FU, and this diminished the anticancer potency of the original 5FdU drug (Chaudhuri et al. 1959). Although clinically valuable, 5FdU discloses off‐target side effects mainly in the gastrointestinal tract and bone marrow, moreover, it exhibits low oral absorption, which results in expensive and often painful therapy in the form of intravenous infusions for patients. To improve clinical efficacy of 5FdU, different strategies for its delivery are being explored, for example, an important prodrug approach (Wagner et al. 2000) or a newer hybrid drugs methodology (Meunier 2008; Corso et al. 2019). Nucleoside‐based drugs are also being studied in their conjugated form (Szymańska‐Michalak et al. 2016; Baraniak, Baranowski, et al. 2019; Baraniak, Ruszkowski, et al. 2019; Michalska et al. 2019; Cardona‐G et al. 2021). The other sense of searching for new and modifying existing NAs is to introduce them into therapeutic oligonucleotides, as native structure‐based oligonucleotides exhibit low binding affinity, weak nuclease resistance, poor cellular uptake, and are rapidly excreted from the body (Wan and Seth 2016; Bege and Borbás 2022; Anwar et al. 2023; Egli and Manoharan 2023). The development of nucleic acid medicines is based on chemical technologies, of which the most important include antisense therapy (ASOs) and RNA interference (siRNAs), and both work on targeting the molecular messenger (mRNA) to stop the synthesis of proteins related to diseases (Kawamoto et al. 2023). Various methods have been used to improve the drug‐like properties of oligonucleotides by heading for their increased binding affinity toward RNA targets and improvement in stability against nucleases to slow down degradation, and all this is achieved by the introduction of chemically modified nucleosides and/or linkers between them (Kulkarni et al. 2021; Kumar and Brown 2022; Van Giesen et al. 2023).

As a proof‐of‐concept, an aptamer intrinsically comprising 5‐fluoro‐2′‐deoxyuridine for targeted chemotherapy was designed (Kruspe and Hahn 2014). Upon degradation of the aptamer by intracellular nucleases, the active drug was released within the targeted cells exclusively. In this way, the aptamer acted as a prodrug meeting two major prerequisites of a drug delivery system: specific cell targeting and the controlled release of the drug triggered by an endogenous stimulus. With the example reported here, the modified aptamer with a single fluorinated nucleoside inside fulfills the requirements for an active drug targeting in an unprecedented manner. Alternatively, the aptamers with high specific binding affinity to their targets can be built from various therapeutic nucleoside analogues, which are released from drug‐integrated DNA after endocytosis. This solution has been known from 2022 and is one of the promising strategies for drug delivery system in cancer therapy; it is named drug‐constituted DNA‐like oligomers system, in short called drugtamers (Zhu et al. 2022). The enzyme‐mediated release of active therapeutics can strongly induce the apoptosis of cancer cells and inhibit tumor progression, which enables a new potential for a better targeted cancer therapy. The results also reveal that drugtamers can effectively inhibit the tumor's development with no obvious side effects. Additionally, modifying the phosphodiester (PO) internucleotide linker is widely investigated to induce and enhance the therapeutic properties through introducing various artificial connections therein nonphosphorus backbones like cationic guanidine (Blaskó et al. 1996) as well as charge‐neutralized triazoles (Amblard et al. 2009; Sharma and Watts 2015). In the last case, the modification of internucleotide linkages is conveniently provided by the click chemistry approach, in particular using the copper(I)‐catalyzed azide‐alkyne cycloaddition (CuAAC) (Kondengadan et al. 2023).

The CuAAC reaction is regarded as a prime example of “click” reaction, because it turned out to be a promising tool to create triazole‐linker compounds also as invaluable support in medical use (i.e., malignant diseases) (Thirumurugan et al. 2013; Bozorov et al. 2019; Xu et al. 2019). From this moment, the click chemistry has been rapidly becoming a reality because of the revolutionary properties of the triazole moiety and increased investment in medicine and pharmaceutical field (Maitz et al. 2022; Peplow 2023; Guan et al. 2024), that was distinctly confirmed by awarding the Nobel Prize in Chemistry 2022 “for the development of click chemistry and bioorthogonal chemistry”. The 1,2,3‐triazole linkage is unique (Kolb and Sharpless 2003). Here, the synthesis of “clickable” nucleoside analogues is also a burgeoning field and given their broad biological remit, there is a continued requirement to explore new nucleoside chemotype structure–activity relationship space (Meanwell et al. 2020; Khandazhinskaya et al. 2026). However, when searching for compounds such as nucleoside dimers with a 1,2,3‐triazole linker dedicated to anticancer research, only a few examples can be found in the literature (Trznadel et al. 2019; Saftić et al. 2023). More often triazole structural elements were introduced to modify nucleic acids (Baraniak and Boryski 2021; Clavé et al. 2021; Fantoni et al. 2021; Perrone et al. 2021; Sharma et al. 2024). It was generally assumed that triazole‐bridged oligonucleotides may be more chemically stable and resistant to nucleases, thus improving the therapeutic properties of nucleic acids.

The pioneers in obtaining triazole oligonucleotides as replacements for internucleotide bonds were Dondoni (Nuzzi et al. 2007) and Isobe (Isobe et al. 2008). These two have extensively tested thymidine in order to obtain oligothymidine analogues, which were to be used as structural models to construct modified nucleic acids intended for antisense oligonucleotides. It was assumed that by removing the charge repulsion associated with bringing two negative strands together, neutral linkages should allow for increased binding affinity. The fundamental study gave a positive answer, namely the triazole‐linked analogue of DNA (10‐mer ^TL^DNA) formed a stable double strand with the complementary strand of natural DNA (^TL^DNA:DNA duplex). After the initial results of the thymidine decamer with five‐bond triazole linkage (as in natural distance corresponding to the phosphodiester bond) in the double strand hybridization, further research was undertaken. The aim was to check whether the effect of a single triazole change on the durability and stability of duplexes compared to their complete modification (Sharma and Watts 2015; Clavé et al. 2021). The experiences led to the general conclusions that a fully triazole‐linked sequence of an oligo‐dT ^TL^DNA analogue showed high binding to complementary DNA (Isobe et al. 2008), but a single triazole linkage within a phosphate‐linked oligonucleotide reduces binding affinity (Varizhuk et al. 2012). Subsequent studies investigated the use of nucleosides other than thymidine, creating a library of four canonical nucleoside analogues bearing clickable groups for ^TL^DNA oligonucleotides design and synthesis (Fujino et al. 2010). Achievements in chemical synthesis enabled the exploitation of triazole‐modified nucleic acids for physicochemical research such as studies on improving their solubility by using triazole ring substitution with a methyl group (Fujino et al. 2013). A one‐step methylation of electroneutral triazole in the pentameric oligothymine ^TL^DNA not only changes into a positively charged triazolium variant ^TL^DNA+ with dramatically improved solubility, but also formed a stable triple helix with natural oligoadenine DNA, as well as a mercury‐mediated self‐duplex. However, in most cases, triazole as a substitute for internucleotide bonds has a destabilizing effect on nucleic acid duplexes. Despite this fact, combining two additional combinations can improve their binding affinity. Increased hybridization, in other words enhanced DNA duplex stability, is possible when triazole linkage is in connection with modified nucleobases such as G‐clamp (El‐Sagheer and Brown 2014) or combined with LNA sugar (Kumar et al. 2017; Sharma et al. 2017; Kumar et al. 2018). Also, the formation of stable duplexes can be achieved when complementary DNA strands were covalently cross‐linked between bases (Kočalka et al. 2008).

Consistently chemical advances have provided new molecular tools for biologists. The new connections with a triazole linker can be obtained by a “quick click”, which is as rapid as ligase and also more selective (Osman et al. 2018). Testing triazole‐modified oligonucleotides potential for their biocompatibility was started by Brown, who simultaneously revealed the valuable properties of the triazole ring (El‐Sagheer and Brown 2012, 2015). Here, due to the scope of the material presented, the examples given will be limited to the aspects regarding ^TL^DNA series. The first literature reports concerned DNA synthesis using click chemistry in the following methods: polymerase chain reaction (PCR) (El‐Sagheer and Brown 2009), the rolling circle amplification (RCA) (Chen et al. 2018), and reverse transcription (RT) (Fujino et al. 2011). For in vitro replication, PCR or RCA templates containing an artificial linkage were obtained by ligation or cyclization clickable oligonucleotides, respectively. In both cases the triazole linker formed by CuAAC method was correctly read‐through by various DNA polymerases and enabled copy DNA on ^TL^DNA template. In turn, during in vitro reverse transcription complementary DNA (cDNA) was synthesized on mRNA template and in the presence of short ^TL^DNA which served as a primer surrogate, and RTase enzyme. It was shown that DNA synthesis was not inhibited by the triazole linkage, but it was not clear wheater this would also be true for living systems. Encouraged by in vitro experiments scientists investigated in vivo replication within the cellular machinery of *Escherichia coli* by constructing a plasmid containing the BLA gene (TEM‐1 β‐lactamase) with the triazole insert (Dallmann et al. 2011; El‐Sagheer et al. 2011). The click linkers were introduced via modified PCR primers that amplified the BLA gene, next after transfection *E. coli* and DNA sequencing, it was confirmed that the sequence around the triazole was copied correctly. So the initial experiment proved that the triazole plasmids replicated accurately by the *E. coli* polymerases to give the expected amplicons and thus confirm its functionality in bacteria. The examples mentioned showed that the triazole linker is a plausible phosphodiester surrogate, but for an arificial DNA backbone linkage to be truly bicompatible it must be also functional in other fundamental biological processes, particularly DNA‐templated RNA synthesis. The first RNA synthesis by in vitro transcription through a purely synthetic analogue of a DNA backbone was achieved with T7 RNA polymerase (T7‐RNAP) (El‐Sagheer and Brown 2011). It was demonstrated that T7‐RNAP enzyme transcribed across a triazole‐modified DNA template to synthesize fully complementary and biologically active RNA (the selected RNA transcript contains the 54‐mer DicF sequence which inhibits the growth of *E. coli* ) in good yield. Following on from the above successful in vitro experiments the properties of triazole‐linked DNA in living organisms were investigated to determine whether native RNA polymerases (RTase) can tolerate the triazole linkages. The biocompatibility of a triazole mimic of the DNA phosphodiester linkage in *E. coli* was re‐evaluated on a non‐essential gene for correct bacterial function and survival (Sanzone et al. 2012). In vivo experiment on the click‐linked mCherry gene was probed via a plasmid containing ligated click DNA backbone in each strand of the gene encoding the red‐fluorescent protein mCherry. The click‐modified plasmid was found to encode mCherry at a similar level to the canonical equivalent. Additionally, the click‐linked DNA is replicated with high fidelity despite the very close proximity of two triazole backbone linkers (4‐bp apart). The ability of the cellular machinery to read through the modified mCherry gene was further probed by using the above click‐linked plasmid to express mCherry in an in vitro transcription/translation system. Efficient protein production from the click‐containing mCherry gene led to the formation of functional mCherry protein at similar quantity to that from the equivalent canonical DNA (the yield of the mCherry protein expression from the click‐linked plasmid was the same as for natural plasmid). To assess the fidelity of transcription through the click‐linked mCherry gene, mRNA was isolated from injected cells and reverse transcribed. The mCherry cDNA was amplified by PCR and cloned to enable the analysis of individual copies of the gene. The sequencing confirmed that the modified mCherry gene was faithfully transcribed and contained no deletions or mutations. Next, in order to determine whether biocompatibility could be achieved in eukaryotic cells, which have more complex biochemistry, a plasmid containing triazole‐linked DNA (a non‐natural DNA‐backbone linker) encoding mCherry was tested in human MCF‐7 breast cancer cells (Birts et al. 2014). Upon transfection the single‐cell analysis of fluorescent protein expression identified correct transcription and translation of the click‐linked gene in human cells. Furthermore, reverse transcription of mRNA isolated from these cells and subsequent sequencing of the mCherry cDNA shows error‐free transcription. These results strongly suggest that RNA polymerase II, the enzyme responsible for all mRNA synthesis in eukaryotes, correctly transcribes the genetic information contained on a click‐linked strand of DNA. Following the above discoveries, it was valuable to investigate whether the click ligation strategy would enable the synthesis of an entire click‐containing gene at once. The first report of a fully chemical one‐pot method described the assembly of a 335 base pair gene for encoding the iLOV green fluorescent protein (Kukwikila et al. 2017). The resulting click‐iLOV gene from 10 clikckable oligonucleotides had eight triazole modifications and was fully biocompatible – it was correctly replicated by DNA polymerase in vitro and encoded the functional iLOV protein in *E. coli* . Interestingly, the genes replicated from the click‐iLOV template had a lower number of genetic mutations compared to a natural analogue produced by enzymatic ligation of 10 canonical oligonucleotides. Also the power and potential of this enzyme‐free gene assembly method was revealed by preparing an epigenetically modified variant of the iLOV gene.

Achievements in the field of molecular biology confirmed that triazole‐modified oligonucleotides have the potential to construct therapeutic nucleic acids, which may ultimately have clinical applications. Therapeutic oligonucleotides are designed to interact with specific genes, including carcinogenic, for example: BCL2 (inhibits apoptosis), KRAS (guided proliferation), and MYC (code transcription factors) (Xu et al. 2026). Oligonucleotide therapy is mainly based on antisense mechanisms (blocking the mRNA of oncogenes and inhibiting the production of proteins) and RNA interference (they may be an element of systems similar to RNA interference, leading to the degradation of the mRNA of the cancer cell). Although most triazole oligonucleotides are still at the stage of chemical and biochemical research, some scientific reports revealed their value as potential anticancer agents (Dysko et al. 2022; Ghosh et al. 2025).

## 2 Results and Discussion

### 2.1 Chemical Synthesis

The first stage involved preparation of 3′‐azido‐ and 4′‐ethynyl‐2′,5′‐dideoxy‐5′‐nor pyrimidine derivatives as substrates for nucleoside dimers synthesis (Schemes 1 and 2, respectively). Three 3′‐azidonucleosides, namely 3′‐azido‐2′,3′‐dideoxyuridine (**21a**, AddU), 3′‐azido‐thymidine (**21b**, AZT), and 3′‐azido‐5‐fluoro‐2′,3′‐dideoxyuridine (**21c**, AFddU) were obtained as follows: **21a** according to modified Balagopala method (Balagopala et al. 1996), **21b** was purchased, and the procedure described in previous work was used for **21c** (Baraniak, Baranowski, et al. 2019). Two‐step synthesis of AddU is based on the Mitsunobu reaction conditions (PPh_3_, DEAD or DIAD in ACN at −15°C) followed by azidation reaction (NaN_3_, *p*‐TsOH, DMF, 120°C) at the 3′ position (Scheme 1) (Balagopala et al. 1996). In the first step, 2′‐deoxyuridine (**19a**) was converted into 2,3′‐anhydro‐2′‐deoxyuridine (**20**) via intramolecular cyclization with *ca*. 88% yield and then the first‐step reaction product was transformed into **21a**.

**SCHEME 1:**
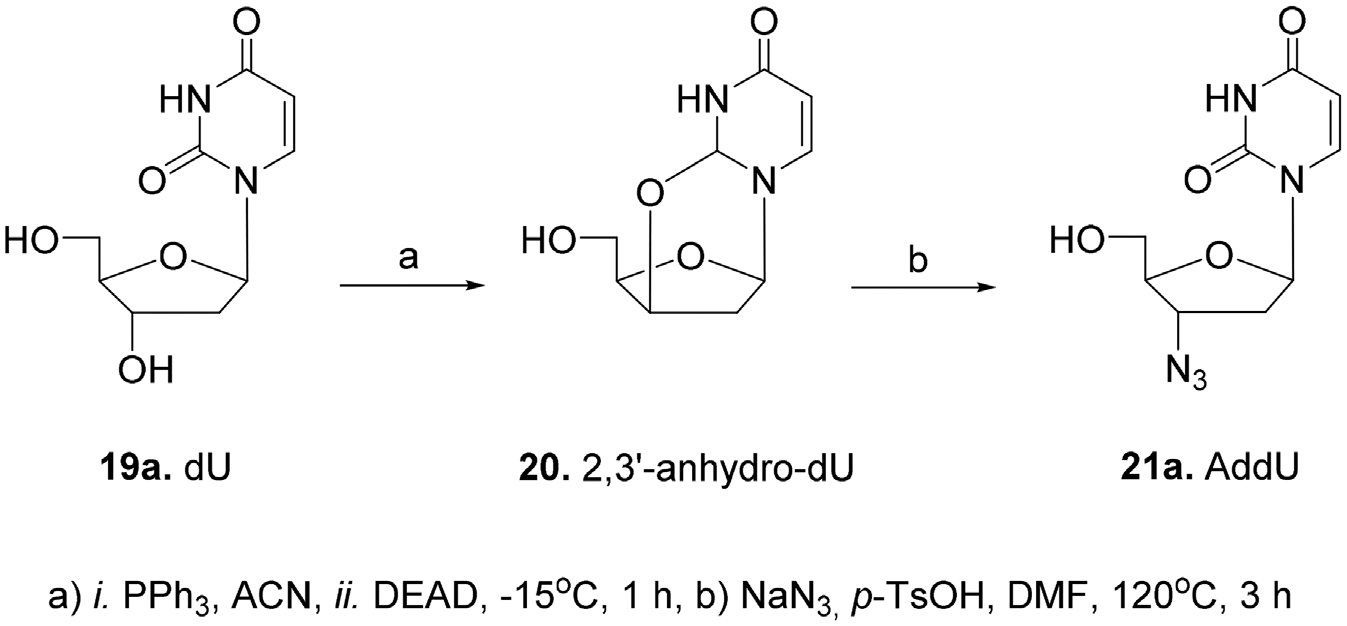
The synthesis of 3′‐azido‐2′,3′‐dideoxyuridine (AddU).

**SCHEME 2:**
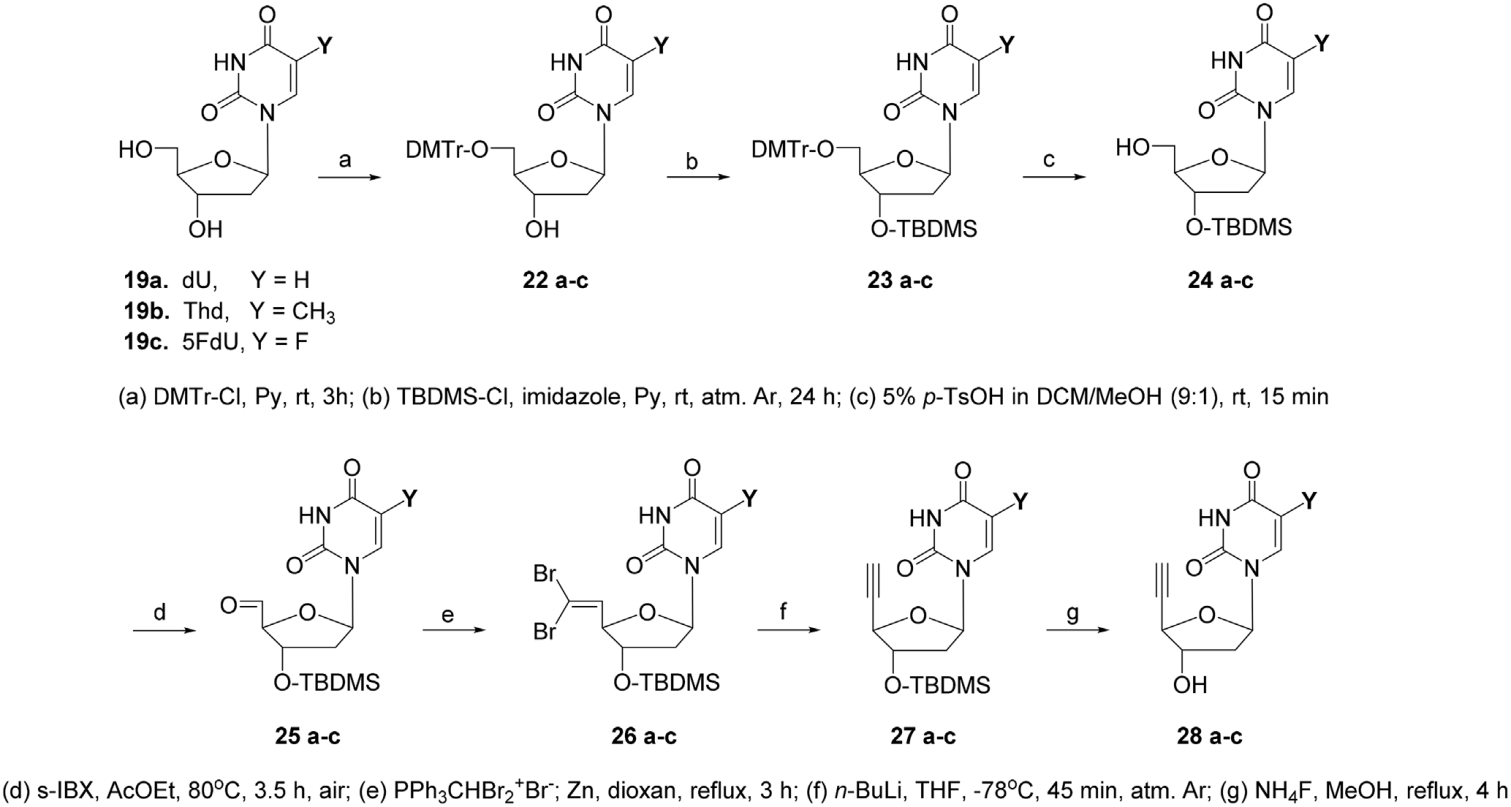
The synthesis of 4′‐ethynyl‐2′,5′‐dideoxy‐5′‐nor pyrimidine nucleoside analogues.

The three 4′‐ethynyl‐2′,5′‐dideoxy‐5′‐nor pyrimidine nucleoside analogues, that is, **28a–c** comprising uracil, thymine or 5‐fluorouracil, respectively, were synthesized using Florentiev method (Scheme 2) (Varizhuk et al. 2011). Generally the synthesis consisted of seven steps, of which the first three included preparation of a key‐intermediate **24a–c** until the appropriate reactions followed. In order to obtain **24a–c** the three consecutive reactions were carried out, namely: protection of the 5′‐hydroxyl group with 4,4′‐dimethoxytrityl chloride (DMTr‐Cl) in pyridine using a Khorana's procedure (Smith et al. 1962; Schaller et al. 1963), then silylation of the 3′‐hydroxyl group with *tert*‐butyldimethylsilyl chloride (TBDMS‐Cl) in pyridine (Ogilvie 1973; Ogilvie et al. 1979), and removal of the DMTr‐ group with a 5% solution of *para*‐toluenesulfonic acid in a mixture of methylene chloride and methanol (5% *p*‐TsOH in DCM/MeOH, 9:1, v/v) (Jl et al. 1990). The total yield for the 3′‐*O*‐(*tert*‐butyldimethylsilyl) pyrimidine nucleosides **24a–c** synthesis was about 70%. In the next stage, four consecutive reactions were carried out starting with the oxidation of **24a–c** with 2‐iodoxybenzoic acid (s‐IBX) in anhydrous ethyl acetate (AcOEt) at 80°C to an aldehyde **25a–c**, which was subjected to Wittig condensation with dibromomethyltriphenylphosphonium bromide (PPh_3_CHBr_2_^+^Br^−^) in the presence of zinc (Zn) in anhydrous dioxane under reflux (Dolhem et al. 2002) to obtain di‐bromovinyl derivative **26a–c**. Finally, two further reactions involved the elimination reaction of **26a–c** in the presence of *n*‐BuLi in anhydrous tetrahydrofuran (THF) at −78°C to an alkyne derivative **27a–c** followed by the removal of the TBDMS group with ammonium fluoride (NH_4_F) in anhydrous MeOH, yielding the final product **28a–c** (Robins et al. 2006). Structure of **27a–c** and **28a–c** were confirmed by NMR spectroscopy (see discussion and Tables 1 and 2 in Section 2.2, and spectra in Supporting Information) and were characterized by ESI‐MS method.

**TABLE 1:** ^1^H NMR spectral data for compounds **27a–c** and **28a–c** ^a^.

	NH	H‐6	H‐1′	H‐2′	H‐2″	H‐3′	H‐4′	H‐6′
**Thd**	11.30	7.71	6.18	2.08		4.26	3.77	—
**27a**	11.35	7.60	6.18	2.34–2.29	2.24–2.20	4.55	4.47	3.82
**27b**	11.36	7.49	6.21	2.37–2.31	2.21–2.15	4.56	4.45	3.86
**27c**	11.90	7.83	6.17	2.41–2.34	2.24–2.18	4.57	4.47	3.89
**28a**	11.36	7.60	6.23	2.28–2.23	2.22–2.17	4.38	4.51	3.79
**28b**	11.34	7.50	6.27	2.24–2.21		4.39	4.49	3.85
**28c**	11.88	7.78	6.22	2.31–2.22		4.39	4.52	3.88

^a^ Chemical shifts in DMSO‐d6. Spectra are presented in Supporting Information.

**TABLE 2:** ^13^C NMR spectral data for compounds **27a–c** and **28a–c** ^a^.

	C‐2	C‐4	C‐5	C‐6	C‐1′	C‐2′	C‐3′	C‐4′	C‐5′	C‐6′
**Thd**	150.4	163.6	109.3	136.0	83.73	39.47	70.37	87.21	61.28	—
**27a**	150.4	163.0	102.3	140.2	85.09	39.01	76.74	75.39	80.66	79.16
**27b**	150.4	163.6	109.9	135.8	84.77	38.88	76.82	75.32	80.83	79.22
**27c**	149.0	157.0	140.1	124.4	85.49	38.94	76.54	75.41	80.64	79.35
**28a**	150.5	163.1	102.4	139.9	85.60	38.76	75.60	75.90	81.23	79.16
**28b**	150.5	163.6	109.9	135.6	85.20	38.43	75.60	75.76	81.41	79.17
**28c**	149.0	156.9	140.1	124.2	86.09	38.54	75.46	75.96	81.18	79.41

^a^ Chemical shifts in DMSO‐d6. Spectra are presented in Supporting Information.

The dimer nucleosides **1**–**18** with a short 1,4‐disubstituted 1,2,3‐triazole linker were received using the click chemistry approach (Figure 1 and Scheme 3), namely the 1,3‐dipolar Huisgen cycloaddition catalyzed by Cu(I)‐ions (Breugst and Reissig 2020). The click reaction was carried out between three 3′‐azido‐nucleosides (**21a–c**) and six 4′‐ethynyl‐5′‐deoxy‐5′‐nor‐ nucleoside derivatives (**27a–c**, **28a–c**) under modified Sharpless conditions (Rostovtsev et al. 2002; Varizhuk et al. 2011) however different click reaction conditions have been examined in order to eliminate generation of unwanted byproducts. The first attempt for click reaction used the classic Sharpless conditions, namely: Cu^1+^ as the catalyst generated in situ from Cu^2+^ (0.4 eq CuSO_4_ *x*5H_2_O) by reducing agent sodium ascorbate (0.8 eq NaAsc) in THF/H_2_O (3:1, v/v) at room temperature for 12 h testing the different ratio of substrates on the progress of reaction. The above studies of various reaction conditions did not lead to the expected products; only substrates were observed on TLC. The second approach involved the use of Meldal conditions, that is, Cu^1+^ as the catalyst (CuI, 2 eqs) in the presence of *N*,*N*‐diisopropylethylamine (DIPEA, 3 eqs) and anhydrous ACN as a solvent under argon atmosphere in 4 h at room temperature (Tornøe et al. 2002). As a results of the reaction, a mixture of two products was obtained, that is, 5‐iodo‐1,4‐disubstituted‐1,2,3‐triazole derivative and expected product (Hein et al. 2009; Barsoum et al. 2015). So modification for the Meldal method was applied, which used slight excess of an alkyne to an azide (1.1/1.0 eq, respectively), a smaller amounts of copper(I) catalyst (CuI, 0.4 eq), and larger excess of DIPEA (8 eqs) in the mixture of toluene with dimethylformamide (toluene/DMF, 10:1, v/v) under argon atmosphere in 36 h at room temperature (Nuzzi et al. 2007). The reaction progress for both variants was similar, yielding a mixture of unreacted substrates and products (or no product), and in most cases with low yield. Furthermore, isolation of the products, that is, 5‐iodo‐1,4‐disubstituted‐1,2,3‐triazole derivative and expected dimer by column chromatography was impossible due to the very small *Rf* differences regardless of the eluent used. Literature data indicate the possibility of iodine atom substitution reactions with a proton using a reducing agent such as sodium borohydride (NaBH_4_) in THF (Testa et al. 2014). However, the results of one experiment showed complete conversion but also low yield after column isolation, so this approach was abandoned. These difficulties led to the search for a third solution, which turned out to be the most effective, namely the Sharpless procedure was repeated in a refluxing solvent system (Scheme 3) (Verma et al. 2021). The final procedure resulted in the formation of only one product with a high yield of *ca*. 80%, whose structure was consistent with the desired Dimers **1**–**18** containing 1,4‐disubstituted 1,2,3‐triazole as a linker. Literature data shown that despite the theoretical possibility of producing another thermal product as the 1,5‐regioisomer of the 1,2,3‐triazole (Beutick et al. 2022) or the 5‐iodo‐1,2,3‐triazol as a side product (Hein et al. 2009; Barsoum et al. 2015), we only obtained the designed 1,4‐regioisomer of 1,2,3‐triazole.

**FIGURE 1:**
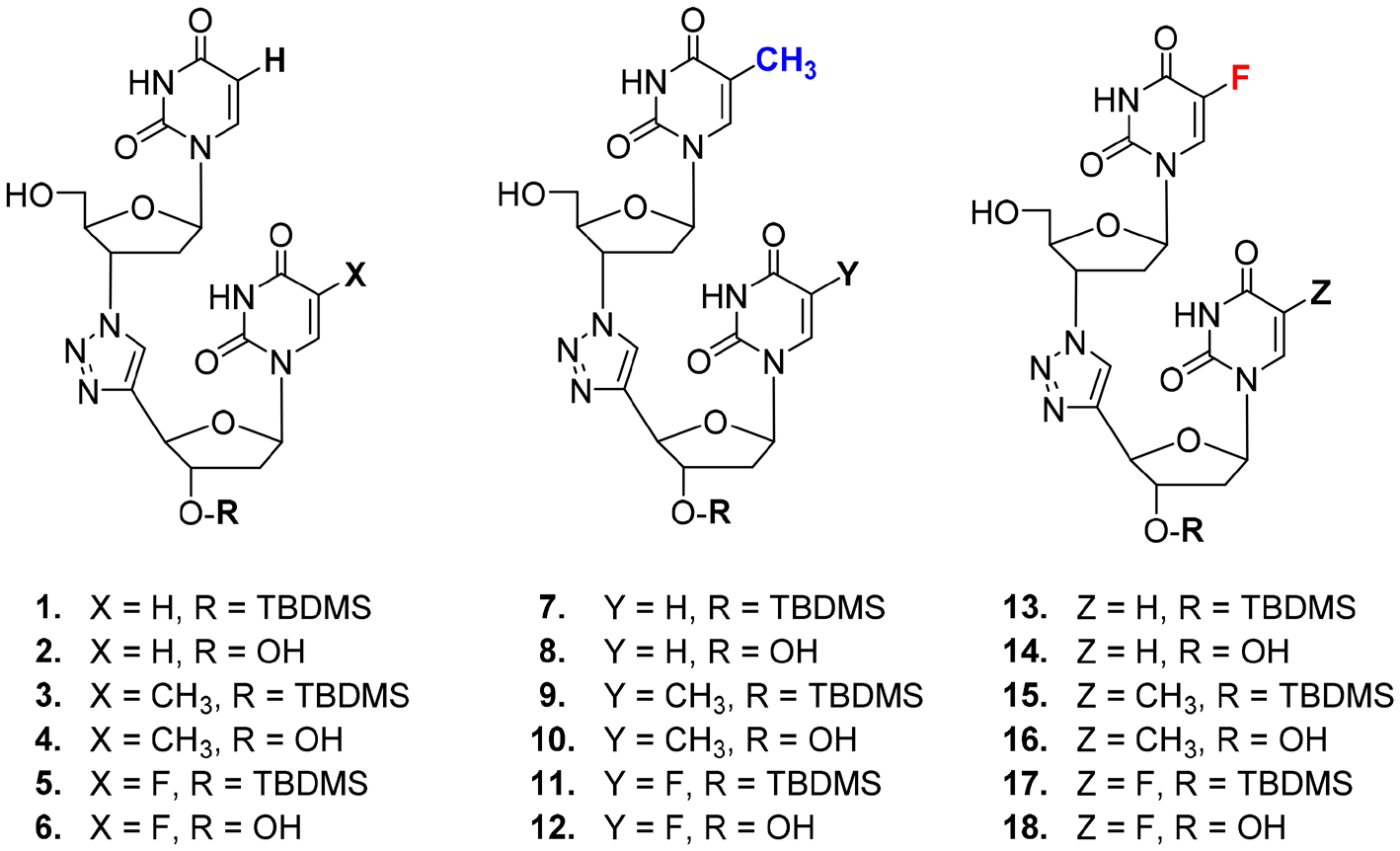
Pyrimidine nucleoside dimers with the 1,4‐disubstituted 1,2,3‐triazole linker.

**SCHEME 3:**
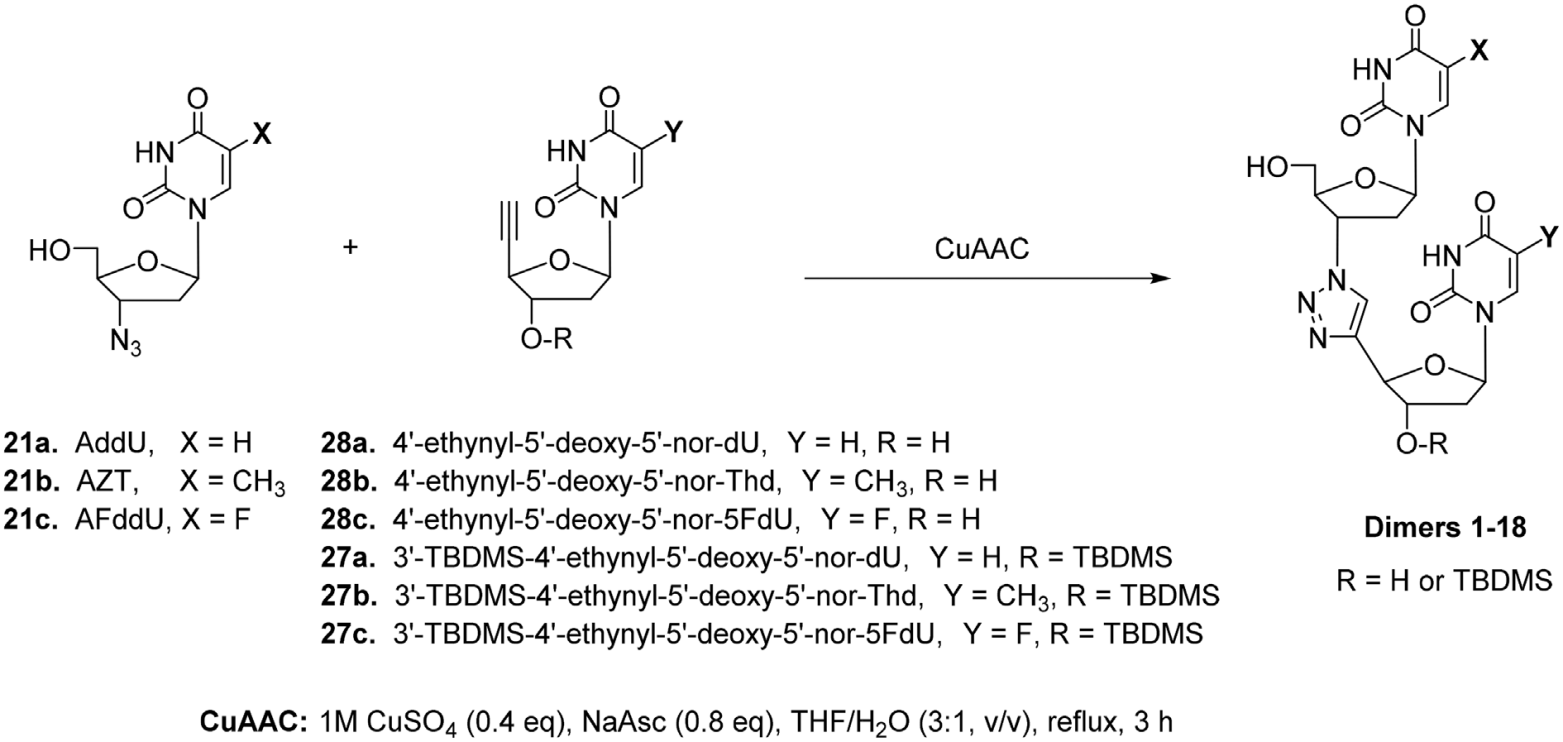
The synthesis of pyrimidine nucleoside dimers with the 1,4‐disubstituted 1,2,3‐triazole linker.

The spectroscopic (NMR), spectrometric (ESI‐MS), and HPLC analysis data of all compounds are included in Figures S1–S235 and were in perfect agreement with their expected structures.

### 2.2 NMR Spectroscopy Analysis

Structure verification of nucleoside monomers (**27a–c**, **28a–c**) and nucleoside dimers (**1**–**18**) were obtained by series of ^1^H, ^13^C, ^15^N, ^19^F, and ^29^Si 1D and 2D NMR measurements. Unambigous assignment of ^1^H, ^13^C, ^15^N resonances was accomplished by careful and comprehensive analysis of 2D spectra (^1^H–^1^H COSY, ^1^H–^13^C/^15^N gHSQC, and gHMBC). NMR data and spectra were provided in Tables 1, 2, 3, 4, 5 and Supporting Information, respectively.

The presence of the H6 proton of the terminal alkyne at the C4′ position of 2′‐deoxyribose of nucleoside monomers was confirmed by a doublet at 3.79–3.89 ppm (^4^ *J*_HH_ ≈ 2.1 Hz), although in a range exceeding the tabulated values of 2.0–3.0 ppm. The observed deshielding effect of the H6 proton resulted primarily from the withdrawing of alkyne π‐electrons by the electronegative oxygen atom O4′, although the influence of the pyrimidine system is not insignificant. The anisotropic effect of triple bond was also noted for H3′ and H4′, that is, downfield shift of resonances by *ca*. 0.3 and 0.7 ppm, respectively, compared with ^1^H NMR data for thymidine (Table 1) (SDBS 2024–2025). Similarly, on the ^13^C spectrum two signals at 80.64–81.41 and 79.16–79.35 ppm corresponding to C5′ and C6′, respectively, clearly confirmed the presence of an alkyne fragment (Table 2). Structure verification of 4′‐alkynyl nucleoside monomers was also obtained by the heteronuclear long‐range correlations C6–H4′ and C5–H3′/H4′ on the ^1^H–^13^C gHMBC spectra (e.g., see Figure S45). The ^1^H and ^13^C chemical shifts changes of pyrimidine moiety substituted with fluorine or methyl group of **27a–c** and **28a–c** were in agreement with previously published NMR data (Baraniak, Baranowski, et al. 2019; Baraniak, Ruszkowski, et al. 2019) and will not be discussed.

The dimeric structure of compounds **1**–**18** was clearly confirmed by the presence of ^1^H, ^13^C, and ^15^N signals corresponding to the sugar and base parts of both monomers and all chemical shifts are summarized in Tables 3, 4, 5. The differences in the ^1^H and ^13^C chemical shifts of both sugar moieties resulted from substitution at the C3′ or C4′ position with an azide group or an alkenyl fragment, respectively. Presence of an azide nitrogen of triazole ring at C3′ caused apparent upfield shift of resonances for C3′A by *ca*. 11 ppm and downfield shift for H3′A by *ca*. 1.1 ppm, compared with NMR data for thymidine (Tables 1 and 2) and were in agreement with previously published results (Baraniak, Baranowski, et al. 2019). Similarly, presence at C4′ position an π‐electron rich alkene triggered upfield shift of resonances for C4′B by *ca*. 7 ppm and downfield shift for H4′B by *ca*. 1.1 ppm. Thus, the signals at 8.41 ± 0.04, 123.4 ± 0.2, and 145.5 ± 0.4 ppm corresponding to H5c, C5c, and C4c, respectively clearly indicate the presence of an alkene fragment in the triazole linker. In case of pyrimidine moieties of dimers **1**–**18** the direction of changes in ^1^H, ^13^C, and ^15^N chemical shifts depending on the type of substituents at the C5 position were in agreement with previously published NMR data (Baraniak, Baranowski, et al. 2019; Baraniak, Ruszkowski, et al. 2019) and will not be discussed.

**TABLE 3:** ^1^H NMR data for compounds nucleoside dimers **1–18** ^a^ (letters A or B correspond to 3′ or 5′ substituted monomer of nucleoside dimer, respectively; *n.d.* = not determined).

	H3A H3B	H5A H5B	H6A H6B	H5 triazole	H1′A H1′B	H2′A H2″A	H2′B H2″B	H3′A H3′B	H4′A H4′B	H5′A H5″A	5′OH 3′OH	^5^CH_3_A ^5^CH_3_B	CH_3_ tBuSi	CH_3_Si
**Thd**	11.30	—	7.71	—	6.18	2.08		4.26	3.77	3.60–3.55	5.04, 5.25	1.78	—	—
**1**	11.35 11.35	5.69 5.69	7.97 7.88	8.42	6.40 6.29	2.78–2.73 2.68–2.62	2.50–2.46 2.31–2.26	5.37 4.63	4.24 4.89	3.72–3.68 3.64–3.60	5.31 —	— —	0.82	0.02 −0.03
**2**	11.36 11.36	5.69 5.69	7.97 7.85	8.37	6.41 6.32	2.79–2.74 2.68–2.62	2.44–2.39 2.31–2.26	5.35 4.48–4.45	4.26 4.92	3.72–3.68 3.66–3.61	5.29 5.63	— —	—	—
**3**	11.34 11.34	5.69 —	7.96 7.67	8.41	6.40 6.30	2.80–2.74 2.69–2.63	2.53–2.48 2.26–2.22	5.38 4.68	4.25 4.89	3.72–3.68 3.63–3.59	5.29 —	— 1.77	0.84	0.03 −0.01
**4**	11.34 11.34	5.69 —	7.96 7.65	8.38	6.40 6.33	2.80–2.75 2.68–2.62	2.46–2.40 2.27–2.23	5.37 4.52	4.26 4.91	3.72–3.68 3.65–3.61	5.30 5.62	— 1.75	—	—
**5**	11.37 11.85	5.69 —	7.97 8.24	8.44	6.40 6.27	2.79–2.74 2.69–2.63	2.53–2.48 2.29–2.24	5.38 4.62	4.25 4.89	3.72–3.69 3.63–3.60	5.30 —	— —	0.82	0.01 −0.04
**6**	11.37 11.86	5.69 —	7.97 8.24	8.40	6.40 6.30	2.80–2.75 2.68–2.63	2.47–2.42 2.30–2.25	5.37 4.46–4.43	4.26 4.93	3.72–3.68 3.65–3.61	5.29 5.63	— —	—	—
**7**	11.35 11.35	— 5.70	7.81 7.87	8.42	6.43 6.29	2.75–2.63	2.50–2.46 2.31–2.26	5.39 4.64	4.21 4.90	3.73–3.69 3.64–3.60	5.29 —	1.81 —	0.83	0.02 −0.03
**8**	11.34 11.34	— 5.66	7.83 7.86	8.41	6.43 6.32	2.76–2.71 2.68–2.62	2.44–2.38 2.31–2.26	5.39 4.48–4.45	4.22 4.93	3.73–3.69 3.66–3.62	5.34 5.66	1.81 —	—	—
**9**	11.34 11.34	— —	7.81 7.67	8.42	6.42 6.30	2.76–2.71 2.69–2.63	2.53–2.48 2.26–2.22	5.39 4.68	4.22 4.89	3.73–3.69 3.63–3.59	5.30 —	1.81 1.77	0.83	0.03 −0.01
**10**	11.33 11.33	— —	7.82 7.66	8.40	6.42 6.33	2.77–2.72 2.69–2.63	2.46–2.41 2.27–2.23	5.39 4.53	4.23 4.91	3.73–3.69 3.65–3.61	5.29 5.62	1.81 1.75	—	—
**11**	11.83 11.34	— —	7.81 8.26	8.45	6.42 6.27	2.77–2.63 2.55–2.50	2.31–2.24	5.40 4.63	4.22 4.90	3.73–3.69 3.64–3.60	5.29 —	1.81 —	0.82	0.01 −0.03
**12**	11.35 11.85	— —	7.82 8.24	8.41	6.43 6.30	2.78–2.62	2.46–2.41 2.31–2.25	5.38 4.45	4.24 4.93	3.73–3.70 3.65–3.62	5.29 5.63	1.81 —	—	—
**13**	11.33 *n.d*.	— 5.69	8.29 7.88	8.43	6.39 6.29	2.78–2.73 2.69–2.63	2.50–2.45 2.30–2.25	5.39 4.63	4.23 4.89	3.74–3.71 3.64–3.61	5.45 —	— —	0.082	0.01 −0.03
**14**	11.84 11.35	— 5.68	8.27 7.86	8.38	6.39 6.32	2.78–2.73 2.68–2.62	2.43–2.38 2.30–2.26	5.37 4.46	4.25 4.92	3.74–3.71 3.66–3.63	5.45 5.66	— —	—	—
**15**	11.88 11.34	— —	8.34 7.67	8.41	6.38 6.30	2.81–2.76 2.71–2.65	2.53–2.48 2.26–2.22	5.39 4.68	4.26 4.89	3.76–3.72 3.64–3.60	5.43 —	— 1.77	0.83	0.03 −0.01
**16**	11.87 11.32	— —	8.34 7.65	8.38	6.38 6.33	2.82–2.76 2.70–2.65	2.46–2.40 2.27–2.23	5.38 4.54–4.50	4.28–4.26 4.91	3.76–3.72 3.66–3.62	5.42 5.61	— 1.75	—	—
**17**	11.88 11.88	— —	8.26 8.34	8.44	6.37 6.27	2.81–2.75 2.72–2.65	2.55–2.48 2.30–2.24	5.43–5.37 4.62	4.26 4.89	3.77–3.72 3.65–3.60	5.43–5.37 —	— —	0.82	−0.01 −0.04
**18**	11.88 11.88	— —	8.23 8.34	8.39	6.38 6.33	2.81–2.76 2.70–2.65	2.47–2.42 2.30–2.25	5.38 4.46–4.43	4.27 4.93	3.76–3.72 3.66–3.62	5.43 5.62	— —	—	—

^a^ Chemical shifts in DMSO‐d_6_. Spectra are presented in Supporting Information.

**TABLE 4:** ^13^C NMR data for compounds nucleoside dimers **1–18** ^a^ (letters A or B correspond to 3′ or 5′ substituted monomer of nucleoside dimer, respectively).

	C2A C2B	C4A C4B	C5A C5B	C6A C6B	C4 triazole	C5 triazole	C1′A C1′B	C2′A C2′B	C3′A C3′B	C4′A C4′B	C5′A	^5^CH_3_A ^5^CH_3_B	CH_3_ tBuSi	CH_3_Si
**Thd**	150.4	163.6	109.3	136.0	—	—	83.73	39.47	70.37	87.21	61.28	12.14	—	—
**1**	150.4 150.4	163.1 163.1	102.2 102.0	140.7 140.6	145.3	123.5	84.33 84.33	37.38 39.51	59.39 75.67	84.67 79.91	60.68	— —	25.60	−4.97 −5.04
**2**	150.5 150.4	163.1 163.0	102.1 102.0	140.7 140.4	145.8	123.3	84.51 84.37	37.37 39.51	59.45 74.08	84.64 80.07	60.79	— —	—	—
**3**	150.5 150.4	163.7 163.1	101.9 109.7	140.7 136.2	145.4	123.4	84.33 84.20	37.38 39.51	59.32 75.70	84.65 79.98	60.63	— 12.33	25.61	−4.94
**4**	150.5 150.4	163.6 163.1	101.9 109.6	140.7 136.1	145.9	123.3	84.34 84.25	37.38 39.51	59.35 74.00	84.63 80.04	60.72	— 12.31	——	—
**5**	150.4 149.2	163.1 157.3	101.9 140.2	140.7 124.8	145.3	123.6	84.34 84.72	37.40 39.50	59.33 75.58	84.64 79.87	60.61	— —	25.59	−4.97
**6**	150.4 149.1	163.1 157.0	101.9 140.1	140.7 124.8	145.8	123.4	84.37 84.97	37.38 38.72	60.72 74.04	84.63 80.11	60.72	— —	—	—
**7**	150.4 150.4	163.7 163.0	109.6 102.2	136.2 140.6	145.3	123.4	83.85 84.33	37.10 39.51	59.35 75.66	84.43 79.91	60.69	12.25 —	25.59	−5.04
**8**	150.5 150.5	163.7 163.1	109.7 102.1	136.2 140.4	145.8	123.3	83.90 84.47	37.08 38.89	59.44 74.08	84.51 80.09	60.79	12.25 —	—	—
**9**	150.5 150.4	163.7 163.7	109.7 109.6	136.2 136.2	145.4	123.4	84.22 83.86	37.12 39.51	59.30 75.71	84.44 79.99	60.65	12.34 12.26	25.61	−4.93 −4.99
**10**	150.5 150.4	163.7 163.7	109.6 109.6	136.2 136.1	145.9	123.2	83.87 84.27	37.11 38.69	59.35 74.01	84.42 80.05	60.74	12.32 12.25	—	—
**11**	150.4 149.1	163.7 157.0	109.6 140.1	136.2 124.9	145.3	123.5	83.87 84.74	37.12 38.75	59.32 75.58	84.43 79.91	60.63	12.24 —	25.59	−4.98 −5.05
**12**	150.4 149.1	163.7 157.0	109.6 140.1	136.2 124.8	145.8	123.3	83.89 84.97	37.11 38.73	59.39 74.07	84.41 80.12	60.73	12.25 —	—	—
**13**	149.6 150.5	157.8 163.1	140.2 102.2	124.5 140.6	145.2	123.5	84.56 84.32	37.40 39.51	59.08 75.68	84.73 79.91	60.46	— —	25.60	−4.97 −5.04
**14**	149.8 150.5	158.1 163.1	140.3 102.1	124.4 140.4	145.8	123.3	84.60 84.52	37.38 38.88	59.18 74.08	84.69 80.08	60.62	— —	—	—
**15**	149.0 150.5	157.0 163.7	140.0 109.6	124.9 136.2	145.4	123.4	84.53 84.20	37.40 39.51	58.91 75.69	84.74 79.96	60.36	— 12.32	25.60	−5.01
**16**	149.0 150.5	157.0 163.6	140.0 109.6	124.9 136.1	145.9	123.3	84.56 84.26	37.39 38.66	58.98 74.01	84.74 80.04	60.47	— 12.31	—	—
**17**	149.0 149.0	157.1 156.9	140.1 140.0	124.8 125.1	145.2	123.6	84.54 84.72	37.42 38.88	58.91 75.54	84.72 79.88	60.34	— —	—	−5.00 5.08
**18**	149.2 149.2	157.2 157.2	140.2 140.2	125.0 124.9	145.9	123.5	84.74 85.12	37.52 38.83	59.19 74.19	84.88 80.19	60.61	— —	—	—

^a^ Chemical shifts in DMSO‐d_6_. Spectra are presented in Supporting Information.

**TABLE 5:** ^15^N NMR data for compounds nucleoside dimers **1**–**18** ^a^ (letters A or B correspond to 3′ or 5′ substituted monomer of nucleoside dimer, respectively, *δ*‐scale with liquid NH_3_ as reference, *n.d.* = not determined).

	N1A N1B	N3A N3B	N1 triazole	N2 triazole	N3 triazole
**1**	−147.1 −146.2	−158.1 −158.1	−254.3	−360.8	−350.8
**2**	−146.8 −146.0	−158.1 −158.1	−254.1	−360.6	−350.5
**3**	−147.2 −143.2	−156.1 −156.1	−254.3	−360.1	−350.7
**4**	−147.1 −143.5	−158.1 *n.d*.	−254.1	−360.7	−350.6
**5**	−147.1 −135.9	−156.1 *n.d*.	−254.2	−360.8	−349.5
**6**	−146.9 −135.6	−158.1 *n.d*.	−254.1	−360.6	−349.6
**7**	−144.0 −146.5	−156.1 −158.2	−254.2	−360.8	−350.6
**8**	−144.1 −146.1	*n.d*. −158.1	−254.3	−360.7	−350.2
**9**	−144.1 −143.2	*n.d*. *n.d*.	−254.2	−360.8	−350.7
**10**	−144.1 −143.2	*n.d*. *n.d*.	−254.3	−360.5	−350.5
**11**	−144.2 −135.7	*n.d*. *n.d*	−254.3	−361.1	−350.1
**12**	−144.2 −135.9	*n.d*. *n.d*.	−254.3	−360.3	−349.0
**13**	−136.5 −146.4	*n.d* −158.5	−253.9	−360.6	−350.7
**14**	−136.3 −146.3	*n.d*. −157.8	−253.8	−360.5	−350.4
**15**	−136.5 −143.4	*n.d*. *n.d*.	−253.8	−360.8	−350.8
**16**	−136.6 −143.5	*n.d*. −156.1	−253.7	−360.5	−350.7
**17**	−136.3 −135.5	−156.1 −156.1	−253.5	−360.5	−349.7
**18**	−135.6 −136.4	*n.d*. *n.d*.	−253.7	−360.1	−349.1

^a^ Chemical shifts in DMSO‐d_6_. Spectra are presented in Supporting Information.

Structure of 1,4‐disubstituted 1*H*‐1,2,3‐triazole as a linker of nucleoside dimers **1**–**18** were confirmed by repetitive patterns of ^1^H–^13^C and ^1^H–^15^N long‐range heteronuclear correlations in gHMBC spectra of all compounds as well as by ^13^C NMR spectra with gated decoupling. In case of ^1^H–^13^C long range correlations between carbons and protons of triazole and ribose moiety the following correlations C5c–H3′a, C5c–H4′b, C3′a–H5c, C4c–H3′b, C4c–H4′b were observed (Figure 2). The aforementioned correlations clearly confirmed the presence of an alkene fragment between position C3′ of monomer A and position C4′ of monomer B in the obtained dimers **1**–**18**. Additionally, ^1^H–^15^N gHMBC connectivities observed for N1c–H2′/2″a, N1c–H4′a, N2c–H3′a, N3c–H4′b were in agreement with literature data that gave us a proof of 1,4‐disubstitution patterns of 1*H*‐1,2,3‐triazole (Figure 2) (Corredor et al. 2013). It is worth mentioning the long‐range internal correlations within the triazole ring, that is, N1c/N2c/N3c–H5c or C4c–H5c (not showed in Figure 2), which further confirm the structure of the triazole linker between the monomers.

**FIGURE 2:**
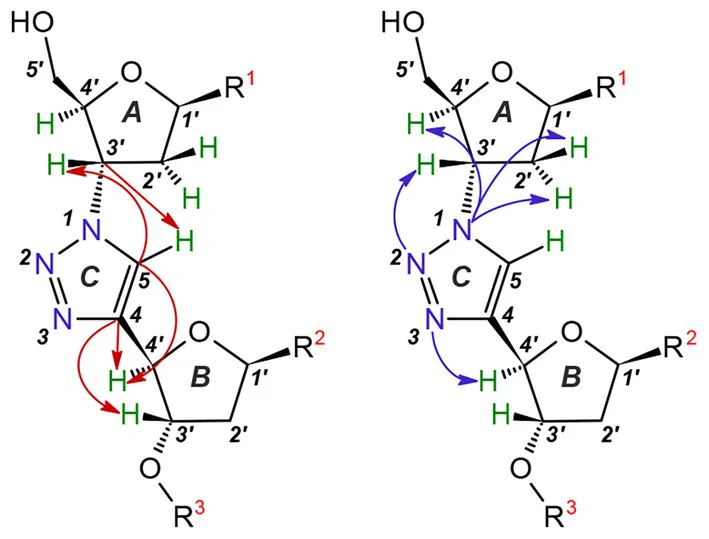
The long‐range heteronuclear correlations in triazole fragment. Red or blue arrows correspond to correlations between ^1^H and ^13^C or ^15^N, respectively. Correlations between H5c and N1c–N3c or C4c were omitted for better clarity.

Structure verification of organic compounds based on ^1^H–^13^C/^15^N gHMBC spectra remains a reliable NMR spectroscopy analysis technique, although distinguishing between 1,4‐ and 1,5‐1*H*‐1,2,3‐triazole may be subjected to uncertainty of the correct interpretation of long‐range correlation signals due to structural similarity of triazole isomers. This uncertainty was unequivocally resolved by the publication of Creary et al. (2012), who demonstrated application of simple one‐dimensional ^13^C NMR spectroscopy using gated decoupling for identification of 1,5‐ or 1,4‐disubstituted 1*H*‐1,2,3‐triazole isomers. Authors excellently proved that chemical shift of triazole tertiary carbons C5 or C4 as well as their one or three bond C–H coupling constants (^1^ *J*_CH_, ^3^ *J*_CH_) were diagnostic for differentiation of disubstitution type of 1*H*‐1,2,3‐triazole moiety. The C5 signal of 1,4‐disubstituted‐1*H*‐1,2,3‐triazoles appears at *δ* = 120 ± 3 ppm, while the C4 signal of 1,5‐disubstituted isomer is shifted downfield and appears at *δ* = 133 ± 3 ppm. For model compounds, that is, 1‐benzyl‐4‐phenyl‐ and 1‐benzyl‐5‐phenyl‐1*H*‐1,2,3‐triazoles, they obtained values of *δ* = 120 ppm ^1^ *J*_CH_ = 191 Hz ^3^ *J*_CH_ = 2.7 Hz (doublet of triplets) and *δ* = 133 ppm ^1^ *J*_CH_ = 195 Hz ^3^ *J*_CH_ = 0 Hz (doublet), respectively. However, taking into accounts all compounds that were investigated in that paper, the value range of ^1^ *J*_CH_ for both isomers were partially overlapped depending on the electronegativity of substituent in position 1,4 or 1,5 of 1*H*‐1,2,3‐triazole ring.

The ^13^C NMR spectra with gated decoupling revealed doublet of triplets for carbon C5c of **1**–**18** nucleoside dimers (Figure 3). The triplets resulted from coupling of C5c via three bonds with proton H3′a and H4′b of ribose A and B, respectively and values of ^3^ *J*_CH_ were 2.30 ± 0.15 Hz falling within observed value range of ^3^ *J*_CH_ in Creary's publication. Conversely, the average one bond C5c–H5c coupling constants was 197.1 ± 0.2 Hz, which was slighty higher by 6 Hz than that reported in Creary's publications. The average chemical shifts of carbon C5c observed for **1**–**18** nucleoside dimers were of 123.4 ± 0.2 ppm. Our results obtained from ^13^C NMR spectra with gated decoupling measurements are in line with Creary's conclusions and support structure of 1,4‐disubstituted 1*H*‐1,2,3‐triazole as a linker.

**FIGURE 3:**
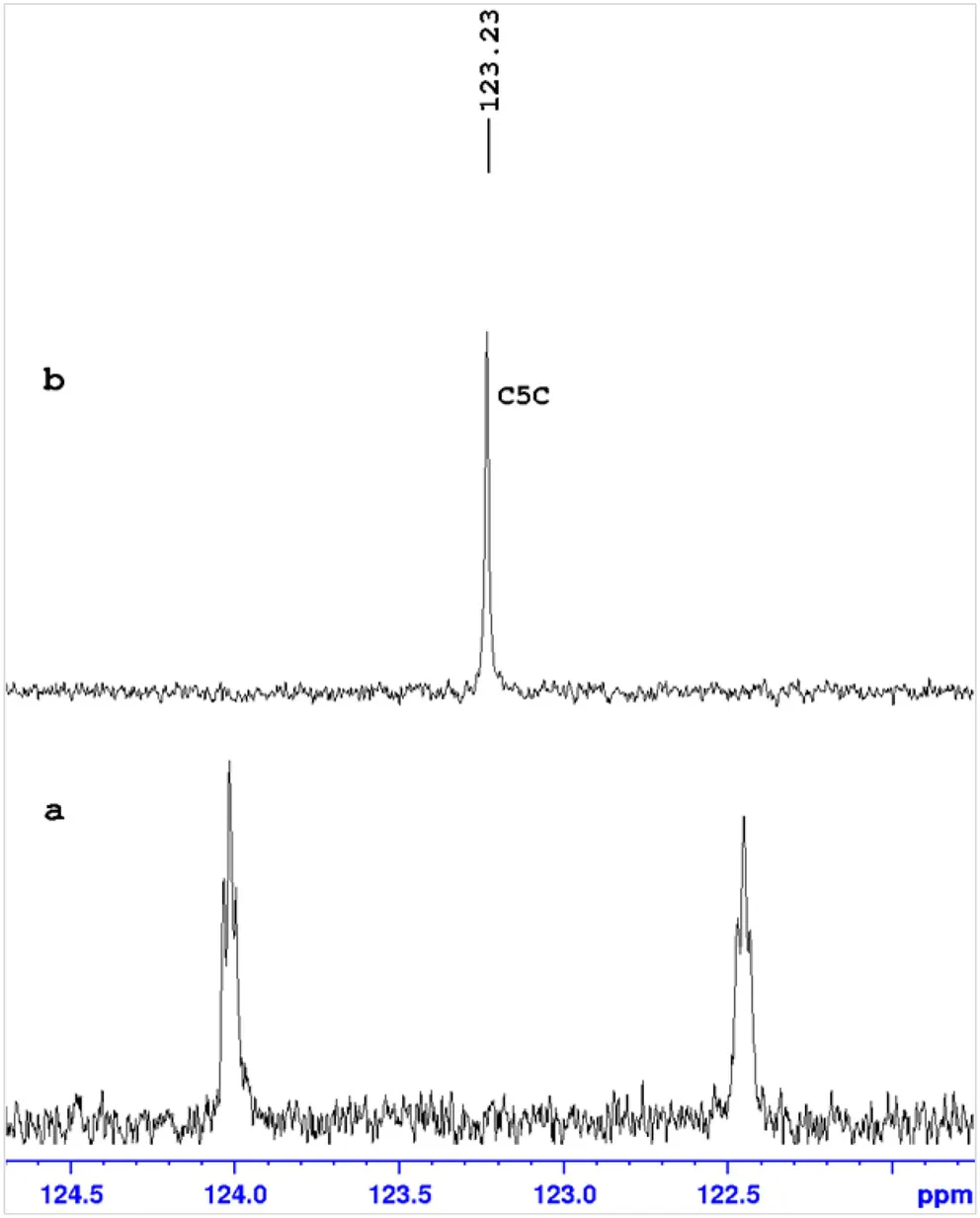
Expanded ^13^C NMR spectra coupled (a) and decoupled (b) with ^1^H for carbon C5c of dimer **10**.

### 2.3 Biological Properties

#### 2.3.1 ADME Prediction and Drug Likeness

SwissADME software was employed to predict the pharmacokinetic properties of novel bioactive nucleoside derivatives with potential medical applications. Key properties, including absorption, distribution, metabolism, and excretion (ADME), are crucial in determining a compound's suitability for human therapeutic use. The prediction process relies on the compound's structure and physicochemical properties. Lipinski's rule of five is a widely used criterion for assessing the drug‐likeness of substances. It takes into account the values of molecular weight of compounds, value of LogP, HBD, and HBA (Figure S236–S262). As can be seen from the data presented in Table 6, all substrates (**21a–c**, **27a–c**, **28a–c**) fulfill this rule. Analysis of the data shows that the compounds have 10 or fewer rotatable bonds and polar surface are much under 140 Å^2^. Such properties suggest a high probability of good oral bioavailability. All compounds are also characterized by high intestinal absorption, which additionally can positively affect their bioavailability. Moreover, two of the studied substrates (**27b**, **27c**) can penetrate the blood–brain barrier. This is particularly noteworthy considering the potential antitumor activity within the brain of these compounds. However, out of the 18 nucleoside dimers (**1–18**) that were obtained, only five (**2**, **4**, **6**, **8**, **14**) adhere to the Lipinski rule, while the remaining 13 do not. This is mainly due to the fact that their MW is > 500 Da. Unfortunately, the high PSA value (above 140 Å^2^) and the hydrophobic nature of these compounds **1**–**18** result in low oral bioavailability and limited distribution to the brain.

**TABLE 6:** Selected physicochemical data for the obtained nucleoside dimers analogues.

Cpd	MW	PSA [Å^2^]	LogP	LogS	HBD	HBA	RB	HIA	BB	Lipinski
Substrates										
**19c**	246.19	104.55	−0.60	−0.76	3	6	2	High	No	Yes
**21a**	253.21	134.07	−0.51	−1.26	2	7	3	High	No	Yes
**21b**	267.24	134.07	−0.15	−1.56	2	7	3	High	No	Yes
**21c**	271.21	134.07	−0.15	−1.55	2	8	3	High	No	Yes
**27a**	336.46	73.32	1.76	−3.25	1	4	4	High	No	Yes
**27b**	350.48	73.32	2.07	−3.56	1	4	4	High	Yes	Yes
**27c**	354.45	73.32	2.09	−3.55	1	5	4	High	Yes	Yes
**28a**	222.20	84.32	−0.27	−0.73	2	4	1	High	No	Yes
**28b**	236.22	84.32	0.02	−0.90	2	4	1	High	No	Yes
**28c**	240.19	84.32	0.05	−0.93	2	5	1	High	No	Yes
Dimers										
**1**	589.67	188.35	0.04	−3.19	3	10	8	Low	No	No
**2**	475.41	199.35	−1.85	−0.65	4	10	5	Low	No	Yes
**3**	603.70	188.35	0.39	−3.51	3	10	8	Low	No	No
**4**	489.44	199.35	−1.56	−0.96	4	10	5	Low	No	Yes
**5**	607.66	188.35	0.43	−3.49	3	11	8	Low	No	No
**6**	493.40	199.35	−1.52	−0.95	4	11	5	Low	No	Yes
**7**	603.70	188.35	0.38	−3.51	3	10	8	Low	No	No
**8**	489.44	199.35	−1.57	−0.96	4	10	5	Low	No	Yes
**9**	617.73	188.35	0.80	−3.83	3	10	8	Low	No	No
**10**	503.47	199.35	−1.32	−1.28	4	10	5	Low	No	No
**11**	621.69	188.35	0.73	−3.81	3	11	8	Low	No	No
**12**	507.43	199.35	−1.24	−1.26	4	11	5	Low	No	No
**13**	607.66	188.35	0.41	−3.49	3	11	8	Low	No	No
**14**	493.40	199.35	−1.56	−0.95	4	11	5	Low	No	Yes
**15**	621.69	188.35	0.74	−3.81	3	11	8	Low	No	No
**16**	507.43	199.35	−1.27	−1.26	4	11	5	Low	No	No
**17**	625.65	188.35	0.70	−3.79	3	12	8	Low	No	No
**18**	511.39	199.35	−1.23	−1.25	4	12	5	Low	No	No

Abbreviations: BB, blood–brain barrier penetration; HBA, number of hydrogen bond acceptors; HBD, number of hydrogen bond donors; HIA, gastrointestinal absorption; Lipinski, match the Lipinski's rules; LogP, an octanol–water partition coefficient; LogS, solubility in water; MW, molecular weight; PSA, polar surface area; RB, rotatable bonds number.

#### 2.3.2 Anticancer Activity (In Vitro Cytotoxicity)

The synthesized compounds were tested for their cytotoxic activity in five human cancer cell lines: cervical (HeLa), gliomas (U‐138 MG, U‐251 MG), breast (T47D), and ovarian (SK‐OV‐3) using the MTT assay. MRC‐5 as referential healthy cells were also used. For comparison, IC_50_ values reflecting inhibitory activity of 5‐fluoro‐2′‐deoxyuridine, (known anticancer drug, **19c**), in the chosen cell lines were included in Table 7. The cytotoxic studies' results are summarized in Table 7. As the table shows, almost all compounds exhibited activity against all tested cancer cell lines. Off all the molecules, the parent drug 5FdU (**19c**) showed IC_50_ value of 49.6 μM against HeLa cell line, 5.3 μM against T47D breast cancer cell line, 12.8 μM against SK‐OV‐3 ovary cancer cell line, and 9.8 and 15.3 μM against two glioma cell lines – U‐138 MG and U‐251 MG. Experiments showed that five nucleoside dimers analogues with a short 1,2,3‐triazole linkage showed high anticancer activity compared to the drug 5FdU. In order from the most active, they are: dimer **11** and **17** with a result IC_50_ = 6.3 μM, dimer **9** with a result IC_50_ = 8.3 μM, and dimer **4** with a result IC_50_ = 8.9 μM, all in HeLa cells. The dimer **9** exhibited also a relatively high activity especially against SK‐OV‐3 cells (IC_50_ = 15.2 μM). The fifth most active compound is dimer **15**, which obtained the highest biological activity in U‐251 MG cells with the result IC_50_ = 4.69 μM. Apart from that, dimers **7**, **12**, **15**, **16** were also highly active. All analogues of pyrimidine nucleoside dimers exhibit satisfactory result in healthy cells, that is low cytotoxicity against normal human fibroblasts (MRC‐5). Among the substrates for dimer synthesis, the most active substrate was 4′‐ethynyl‐5′‐deoxy‐5′‐nor‐5FdU (**28c**). This substrate showed the activity against HeLa cells with IC_50_ = 87.0 μM, against T47D cells IC_50_ was 46.4 μM, against SK‐OV‐3 cells IC_50_ was 28.5 μM, and in U‐251 MG cell line IC_50_ was 24.56 μM. Also, the **27c** substrate (3′‐TBDMS‐4′‐ethynyl‐5′‐deoxy‐5′‐nor‐5FdU) showed high activity in cancer cell lines, IC_50_ values were in the range from 22 μM (T47D) to 91.4 μM (HeLa).

**TABLE 7:** In vitro cytotoxic activity (IC_50_, μM).

	MRC‐5	U‐138 MG	U‐251 MG	T47D	HeLa	SK‐OV‐3
5FdU (**19c**)	> 100	9.8 ± 1.7	15.3 ± 2.2	5.3 ± 0.4	49.6 ± 9.9	12.8 ± 2.8
AddU (**21a**)	53.6 ± 7.7	159.1 ± 28.8	112.9 ± 10.9	21.1 ± 4.6	163.1 ± 13.8	35.8 ± 6.1
AFddU (**21c**)	> 200	> 200	26.51 ± 5.8	163.8 ± 27.3	85.8 ± 18.0	> 200
4′‐ethynyl‐5′‐deoxy‐5′‐nor‐dU (**28a**)	> 200	117.7 ± 24.9	94.64 ± 7.4	100.4 ± 20.5	> 200	> 200
4′‐ethynyl‐5′‐deoxy‐5′‐nor‐Thd (**28b**)	> 200	> 200	38.75 ± 8.5	99.9 ± 23.1	50.6 ± 10.8	186.4 ± 12.6
4′‐ethynyl‐5′‐deoxy‐5′‐nor‐5FdU (**28c**)	> 200	> 200	24.56 ± 1.4	46.4 ± 9.4	87.0 ± 15.1	28.5 ± 6.2
3′‐TBDMS‐4′‐ethynyl‐5′‐deoxy‐5′‐nor‐dU (**27a**)	> 200	> 200	44.07 ± 3.6	53.6 ± 9.1	33.7 ± 4.0	153.7 ± 32.9
3′‐TBDMS‐4′‐ethynyl‐5′‐deoxy‐5′‐nor‐Thd (**27b**)	> 200	151.6 ± 23.5	35.65 ± 5.7	49.1 ± 8.9	34.8 ± 4.5	165.2 ± 28.1
3′‐TBDMS‐4′‐ethynyl‐5′‐deoxy‐5′‐nor‐5FdU (**27c**)	126.0 ± 20.2	> 200	25.42 ± 5.5	22.0 ± 5.0	91.4 ± 16.9	70.6 ± 15.8
Dimer **1**	> 200	98.6 ± 10.6	103.9 ± 21.8	118.2 ± 12.3	> 200	106.8 ± 10.5
Dimer **2**	> 200	142.4 ± 30.2	117.5 ± 20.6	142.4 ± 25.8	171.1 ± 34.5	190.2 ± 8.7
Dimer **3**	> 200	82.37 ± 6.5	96.73 ± 20.1	65.05 ± 7.1	116.6 ± 24.6	> 200
Dimer **4**	> 200	120.1 ± 19.9	50.78 ± 7.1	48.7 ± 6.1	8.9 ± 0.2	139.2 ± 29.3
Dimer **5**	> 200	40.49 ± 6.1	56.4 ± 9.1	40.08 ± 5.8	77.07 ± 9.9	130.1 ± 15.2
Dimer **6**	> 200	109.5 ± 23.2	49.42 ± 4.2	95.55 ± 15.4	162.5 ± 25.5	> 200
Dimer **7**	> 200	79.6 ± 14.2	11.78 ± 1.3	22.9 ± 4.5	47.0 ± 7.5	88.39 ± 2.5
Dimer **8**	> 200	160.9 ± 14.9	88.03 ± 16.4	153.3 ± 10.6	82.78 ± 18.8	> 200
Dimer **9**	> 200	110.6 ± 21.2	38.32 ± 9.2	48.9 ± 11.1	8.3 ± 1.6	15.2 ± 1.9
Dimer **10**	> 200	152.9 ± 24.6	46.41 ± 9.2	104.3 ± 14.6	177.6 ± 27.2	133.1 ± 24.5
Dimer **11**	> 200	59.2 ± 12.3	27.98 ± 4.3	54.5 ± 9.2	6.3 ± 1.1	26.9 ± 4.7
Dimer **12**	> 200	149.8 ± 29.4	40.98 ± 6.5	89.0 ± 18.5	37.5 ± 5.1	13.02 ± 2.1
Dimer **13**	> 200	119.5 ± 19.9	120.5 ± 15.2	156.5 ± 32.5	183.1 ± 26.7	156.1 ± 8.6
Dimer **14**	> 200	188.8 ± 39.1	154.5 ± 29.9	170.3 ± 27.5	171.2 ± 29.1	135.4 ± 14.9
Dimer **15**	> 200	55.4 ± 9.8	4.69 ± 0.9	32.7 ± 4.3	39.4 ± 4.9	108.5 ± 15.2
Dimer **16**	> 200	120.7 ± 22.1	44.2 ± 8.1	25.4 ± 5.2	25.7 ± 5.6	57.8 ± 11.1
Dimer **17**	> 200	97.2 ± 18.9	49.17 ± 7.0	38.9 ± 7.6	6.3 ± 0.4	30.9 ± 3.5
Dimer **18**	> 200	94.0 ± 3.9	> 200	53.7 ± 5.1	69.5 ± 12.3	> 200

*Note:* In vitro cytotoxic activity of the substrates and synthesized compounds in five human cancer cell lines: cervical (HeLa), gliomas (U‐138 MG, U‐251 MG), breast (T47D), ovarian (SK‐OV‐3), and normal human fibroblast cell line (MRC‐5). The IC_50_ values were calculated using GraphPad Prism software.

#### 2.3.3 Functional Analysis (Mechanisms of Anticancer Action)

To better understand the differences in the antiproliferative activity of the synthesized dimers among the tested cancer cell lines, it should be noted that the selected panel was chosen not only to represent different tumor types but also based on their molecular characteristics. In addition to the two glioblastoma cell lines (U‐138 MG and U‐251 MG), cervical (HeLa), ovarian (SK‐OV‐3), and breast (T47D) cancer cells were selected according to transcriptomic data available from The Cancer Genome Atlas (TCGA), which indicate that glioblastoma (GBM), cervical squamous cell carcinoma and endocervical adenocarcinoma (CESC), ovarian serous cystadenocarcinoma (OV), and breast invasive carcinoma (BRCA) frequently exhibit elevated thymidylate synthase (TYMS) expression (Figure 4). Since thymidylate synthase is the primary molecular target of fluoropyrimidine‐based nucleoside analogues, these cell lines constitute a biologically relevant model for evaluating the activity of the synthesized dimers. Nevertheless, despite the common feature of TYMS overexpression, substantial differences in cytotoxicity were observed among the investigated cell lines. This finding suggests that TYMS expression alone is not sufficient to determine cellular sensitivity. Instead, the biological response is likely influenced by additional factors, including differences in nucleoside transporter expression, intracellular nucleoside metabolism, proliferation rate, DNA repair capacity, and apoptotic signaling pathways. Consequently, the observed antiproliferative activity should be considered cell line‐specific rather than tissue‐specific, highlighting the complexity of the molecular mechanisms governing the response to these nucleoside dimers.

**FIGURE 4:**
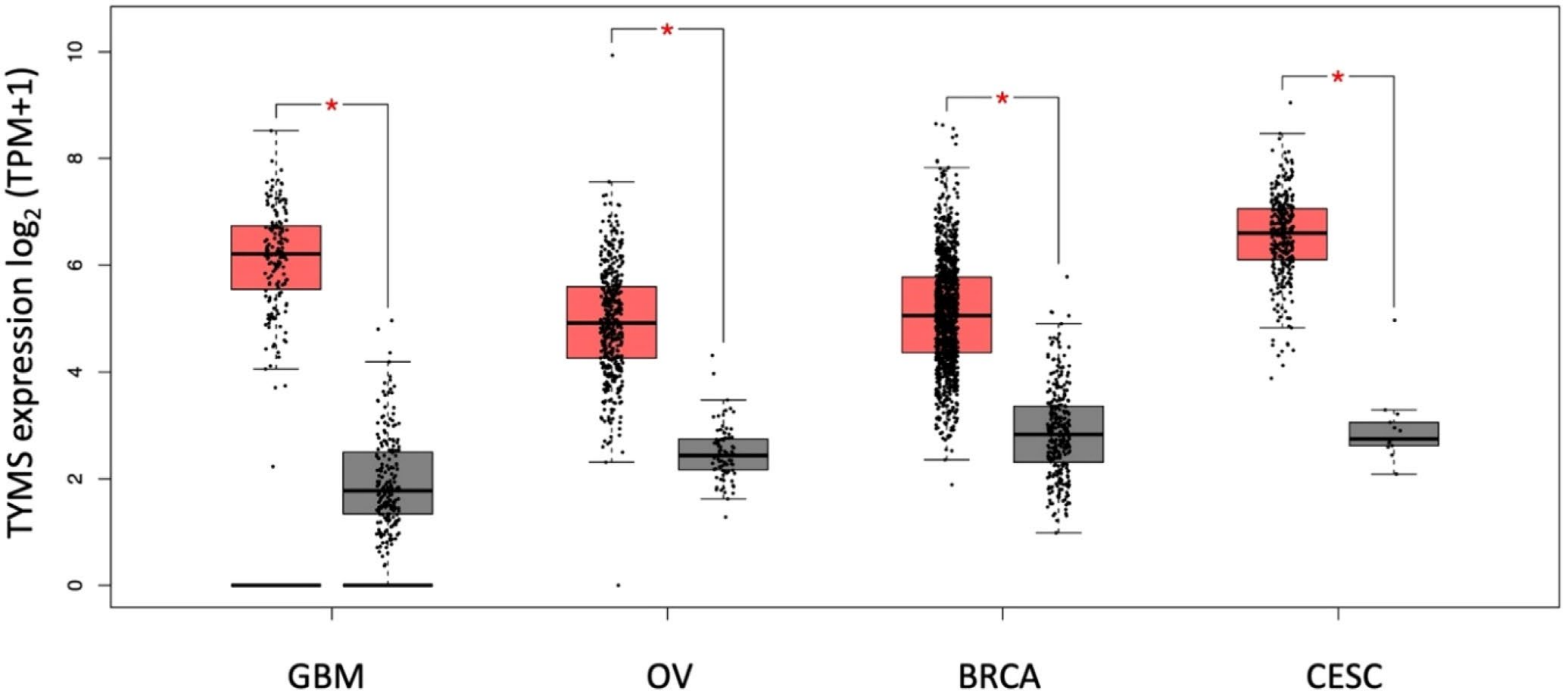
The expression of TYMS in diverse human cancers. The expression levels of TYMS in pan‐cancers were performed by GEPIA database. Red boxes – number of tumor samples (*T*). Gray boxes – number of normal samples (*N*). BRCA – breast invasive carcinoma (*T* = 1085; *N* = 291). CESC – cervical squamous cell carcinoma and endocervical adenocarcinoma (*T* = 306; *N* = 13). GBM – glioblastoma multiforme (*T* = 163; *N* = 207). OV – ovarian serous cystadenocarcinoma (*T* = 426; *N* = 88). *p < 0.05.

##### 2.3.3.1 Live/Dead Cell Staining

To further evaluate the effect of the most active dimers on U‐251 MG cell viability, Live/Dead fluorescence staining was performed after treatment with dimers **7**, **9**, **11**, **15**, **16**, and **17** at concentrations of 10 and 50 μM, using 5‐fluoro‐2′‐deoxyuridine (5FdU) as the reference compound. In this assay, viable cells were stained green, dead cells red, while cell nuclei were counterstained blue (Figure 5). The untreated control and DMSO‐treated cells exhibited predominantly green fluorescence with a dense monolayer of spindle‐shaped cells and only sporadic red‐stained cells, confirming high viability and the absence of solvent‐induced cytotoxicity. Exposure to all tested compounds resulted in a concentration‐dependent reduction in the number of viable cells accompanied by a progressive increase in red fluorescence, indicating enhanced cell death.

**FIGURE 5:**
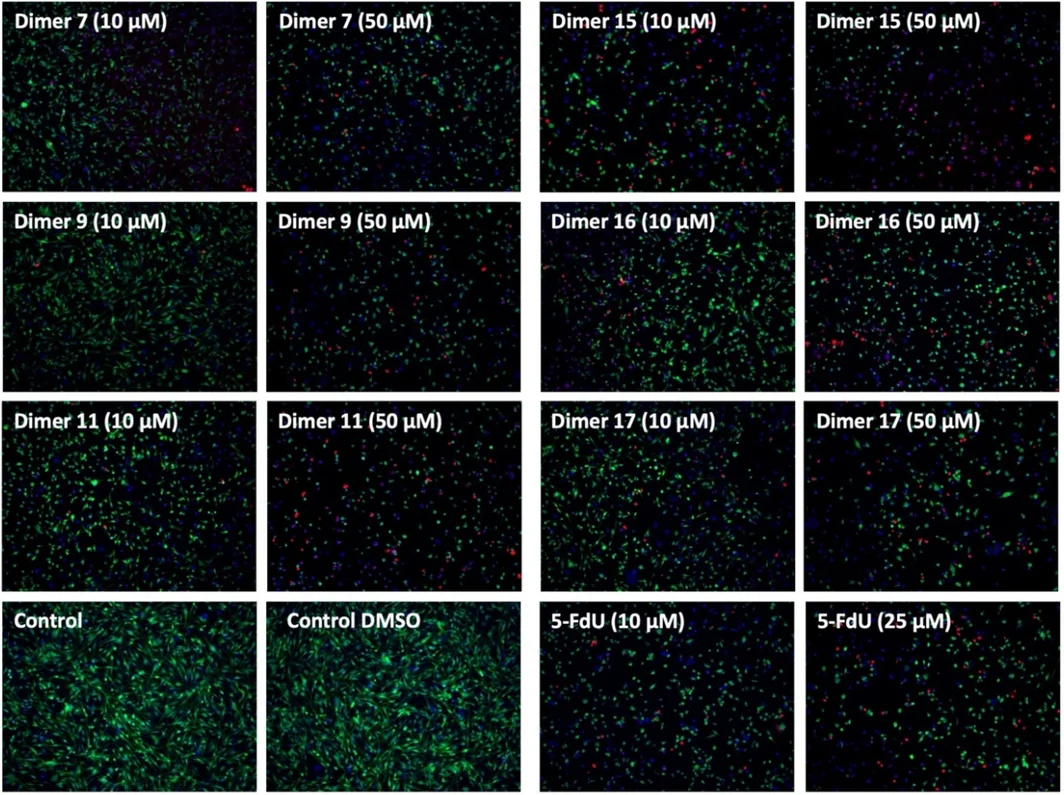
Live/Dead fluorescence staining of U‐251 MG cells treated with selected nucleoside dimers. Cells were incubated with dimers **7**, **9**, **11**, **15**, **16**, and **17** at concentrations of 10 and 50 μM. 5‐Fluoro‐2′‐deoxyuridine (5FdU) was used as the reference compound. Untreated cells and DMSO‐treated cells served as negative controls. Viable cells are stained green (calcein‐AM), dead cells are stained red (ethidium homodimer‐1), and cell nuclei are counterstained blue (Hoechst 33342). Representative fluorescence microscopy images demonstrate a concentration‐dependent increase in cell death, evidenced by an increased number of red‐stained cells and a concomitant reduction in viable (green) cells following treatment with the tested dimers. Representative images were acquired at ×10 magnification.

At 10 μM, the majority of cells remained viable after treatment with all dimers, although an increased number of red‐stained cells compared with the control was already visible, particularly for dimers **7**, **11**, and **15**, as well as for the reference compound 5FdU. Increasing the concentration to 50 μM markedly enhanced the cytotoxic effect of all tested compounds. A substantial decrease in cell density and green fluorescence was accompanied by a clear increase in the number of red‐stained cells, indicating a pronounced loss of viability. In addition, treated cells displayed morphological alterations, including cell shrinkage, rounding, and detachment from the culture surface, which are characteristic of cells undergoing programmed cell death.

Among the tested compounds,d imers **7**, **11**, and **15** produced the most evident increase in dead cells at 50 μM, showing an effect comparable to or exceeding that observed for 5FdU at the same concentration. Dimers **9**, **16**, and **17** also induced a clear concentration‐dependent increase in cell death, although the effect appeared less pronounced. Overall, the Live/Dead assay confirmed that the synthesized dimers induce dose‐dependent cytotoxicity, which is consistent with the antiproliferative activity determined in the MTT assay and supports the conclusion that increasing compound concentration leads to progressive loss of cell viability.

##### 2.3.3.2 Clonogenic Assay

The long‐term antiproliferative effects of the selected dimers on the clonogenic potential of U‐251 MG glioblastoma cells were evaluated using a colony formation assay (Figure 6). Untreated control cells were considered to represent 100% colony‐forming ability. Treatment with all tested dimers resulted in a concentration‐dependent reduction in clonogenic survival. At the lowest concentration (5 μM), dimers **7**, **11**, and **15** reduced colony formation to 75.2%, 77.3%, and 75.1%, respectively, whereas dimers **9**, **16**, and **17** exhibited a weaker inhibitory effect, with surviving fractions of 81.5%, 90.1%, and 90.4%, respectively. Increasing the concentration to 10 μM further decreased the colony‐forming capacity of U‐251 MG cells. The most pronounced inhibition was observed for dimer **11**, reducing colony formation to 57.7% of the control, followed by dimers **7** (67.3%), **9** (69.6%), **15** (69.2%), **17** (71.5%), and **16** (80.8%). The strongest suppression of clonogenic growth was observed at 50 μM, where all compounds markedly impaired the ability of U‐251 MG cells to generate colonies. Dimers **15** (42.3%), **11** (43.6%), and **7** (44.2%) exhibited the greatest inhibitory activity, reducing colony formation by approximately 56%–58% relative to the untreated control. Dimer **9** also showed a pronounced effect, decreasing clonogenic survival to 51.9%, whereas dimers **17** and **16** were comparatively less active, with surviving fractions of 59.6% and 69.1%, respectively.

**FIGURE 6:**
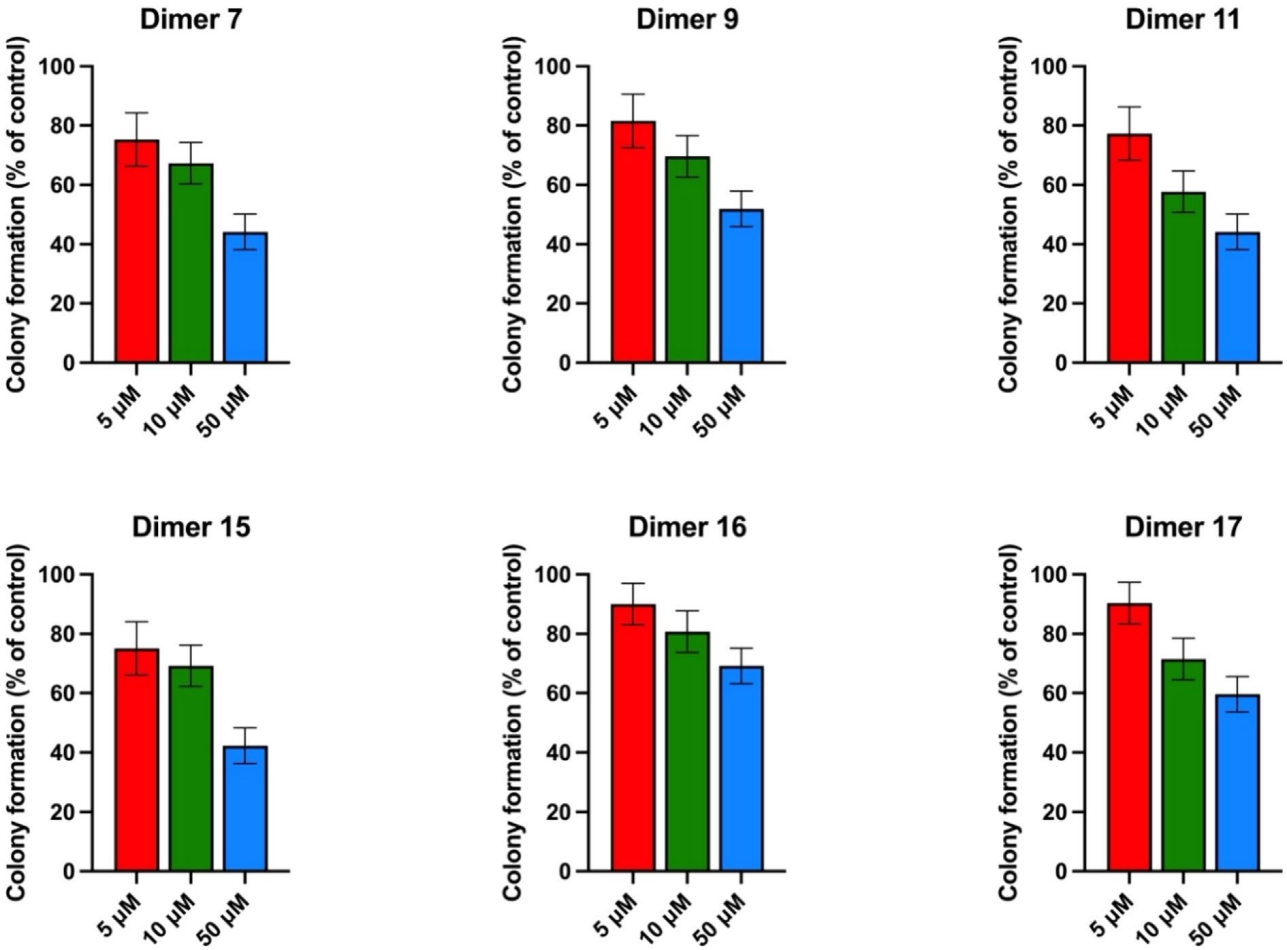
Effect of selected nucleoside dimers on the clonogenic survival of U‐251 MG glioblastoma cells. Cells were treated with dimers **7**, **9**, **11**, **15**, **16**, and **17** at concentrations of 5, 10, and 50 μM and allowed to form colonies for the indicated incubation period. Untreated cells served as the control (100% colony‐forming efficiency). Colony formation was quantified and expressed as the percentage of surviving colonies relative to the untreated control. Data are presented as the mean ± SD of three independent experiments.

Overall, the clonogenic assay demonstrated that all selected dimers inhibited the long‐term reproductive capacity of U‐251 MG glioblastoma cells in a dose‐dependent manner. Among the tested compounds, dimers **11**, **15**, and **7** showed the strongest suppression of colony formation, indicating a sustained antiproliferative effect that extends beyond the acute cytotoxicity observed in short‐term viability assays.

##### 2.3.3.3 Intracellular ROS Generation

To determine whether oxidative stress contributes to the antiproliferative activity of the selected nucleoside dimers, intracellular reactive oxygen species (ROS) levels were measured in U‐251 MG glioblastoma cells using the DCFH‐DA assay following treatment with dimers **7**, **9**, **11**, **15**, **16**, and **17** at concentrations of 5, 10, and 50 μM (Figure 7).

**FIGURE 7:**
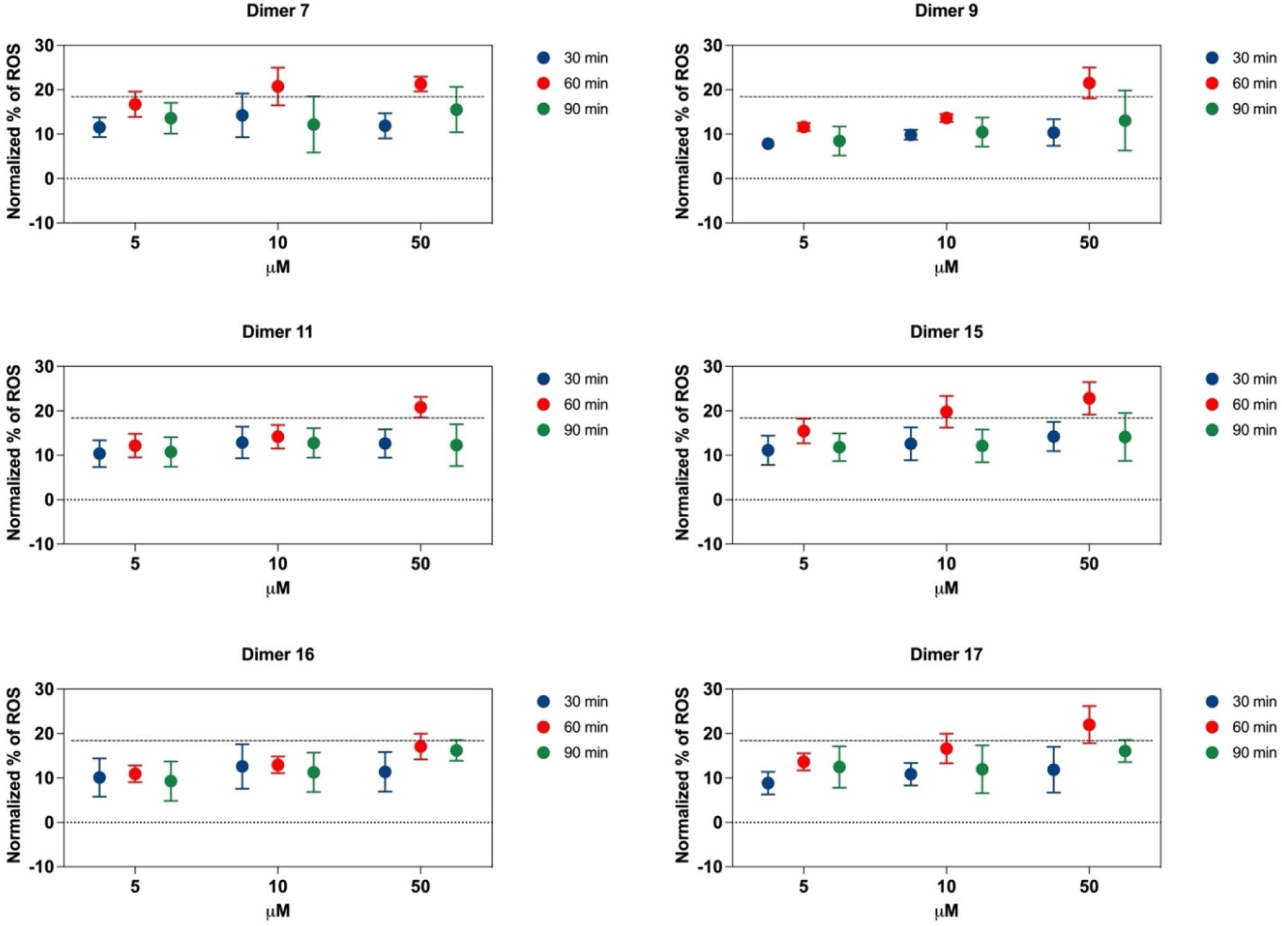
Intracellular reactive oxygen species (ROS) generation in U‐251 MG glioblastoma cells following treatment with selected nucleoside dimers. Cells were treated with dimers **7**, **9**, **11**, **15**, **16**, and **17** at concentrations of 5, 10, and 50 μM. Intracellular ROS levels were determined using the DCFH‐DA fluorescence assay after 30, 60, and 90 min of incubation and are expressed as the percentage increase relative to untreated control cells. Data are presented as the mean ± SD of three independent experiments. The results demonstrate a modest, transient increase in ROS production, with the highest levels observed after 60 min of treatment, followed by a decline toward baseline values at 90 min.

Overall, all investigated dimers induced only a modest increase in intracellular ROS production, which was most pronounced 60 min after treatment. At this time point, ROS levels increased to approximately 20%–23% above the untreated control, particularly following exposure to the highest concentration (50 μM). The most evident ROS generation was observed for dimers **9**, **15**, and **17**, whereas dimers **7**, **11**, and **16** produced a comparable but slightly less pronounced response. At 30 min, ROS levels remained close to baseline, with only minor concentration‐dependent increases, while after 90 min the ROS signal decreased and approached control values, indicating that the oxidative response was transient rather than sustained. Importantly, the magnitude of ROS induction observed for all tested dimers was relatively low. Compounds whose cytotoxic activity is primarily mediated through oxidative stress typically trigger a several‐fold increase in intracellular ROS, frequently exceeding 100% of the control level. In contrast, none of the investigated dimers induced ROS production exceeding approximately 20%–23% above control, even at the highest concentration tested. These findings suggest that although the selected nucleoside dimers are capable of eliciting a small and temporary oxidative response, oxidative stress is unlikely to represent the primary mechanism underlying their antiproliferative activity. Instead, the observed ROS generation is more likely a secondary consequence of cellular stress induced by the compounds rather than the major driver of cell death. This conclusion is consistent with the Live/Dead and clonogenic assays, which demonstrated pronounced cytotoxic and antiproliferative effects despite only minimal ROS induction.

##### 2.3.3.4 Cell Migration Assay

The effect of the selected nucleoside dimers on the migratory capacity of U‐251 MG glioblastoma cells was evaluated using the IncuCyte Scratch Wound Assay. Cell migration was continuously monitored for 96 h, with images acquired every 6 h. Wound closure was analyzed at 24, 48, 72, and 96 h, while the quantitative results presented in Figure 8 focus on the 24 and 72 h time points, which best illustrate the early and late stages of wound healing. Figure 9 shows representative images of the impact of selected nucleoside dimers on U‐251 MG glioblastoma cell migration. In untreated control cells, the relative wound density (RWD) reached 45.39% after 24 h, increasing to 91.74% after 72 h, indicating nearly complete wound closure. Treatment with all tested dimers reduced cell migration in a concentration‐dependent manner, although the magnitude of inhibition differed among the compounds. After 24 h, all investigated dimers exhibited a tendency to decrease wound closure with increasing concentration. At the highest tested concentration (50 μM), statistically significant inhibition of migration was observed for dimers **7**, **9**, **11**, **15**, **16**, and **17**. Among these, the strongest effects were produced by dimer **7**, which reduced the relative wound density to 26.78%, followed by dimer **11** (27.22%), dimer **15** (27.09%), dimer **17** (29.41%), dimer **16** (32.21%), and dimer **9** (34.02%), compared with 45.39% in the untreated control. Lower concentration (10 μM) produced more moderate reductions in wound closure.

**FIGURE 8:**
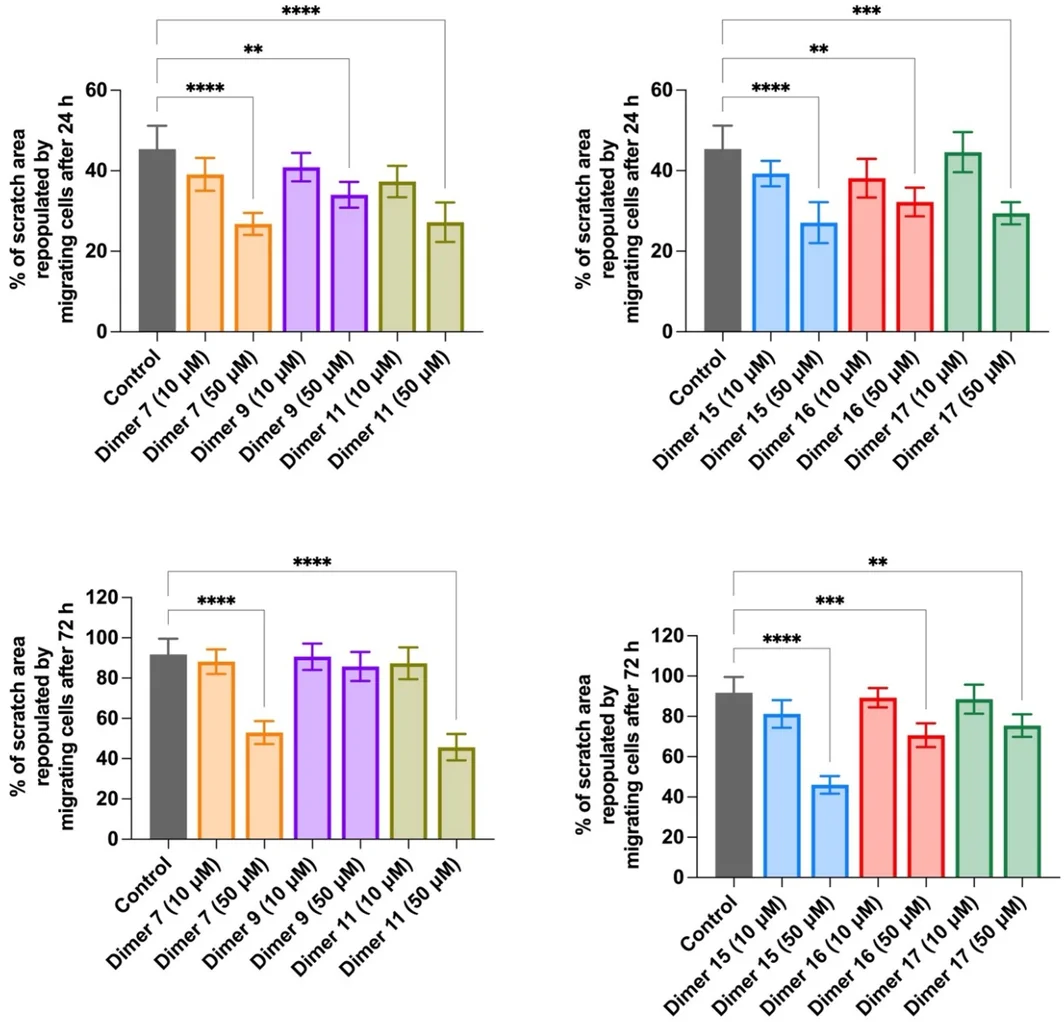
Effect of selected nucleoside dimers on the migration of U‐251 MG glioblastoma cells determined using the IncuCyte Scratch Wound Assay. Cells were treated with dimers **7**, **9**, **11**, **15**, **16**, and **17** at the indicated concentrations (10–50 μM). Cell migration was monitored continuously for 96 h, and phase‐contrast images were acquired every 12 h using the IncuCyte Live‐Cell Analysis System. Wound closure was quantified as relative wound density (RWD). Representative quantitative data obtained after 24 and 72 h of treatment are shown. Data are presented as the mean ± SD of three independent experiments. Statistical significance was determined using one‐way ANOVA followed by Dunnett's multiple comparisons test, comparing treated cells with the untreated control (*p* < 0.05, *p* < 0.01, *p* < 0.001, *p* < 0.0001). Only statistically significant differences are indicated in the figure; the absence of significance markers denotes no statistically significant difference compared with the control.

**FIGURE 9:**
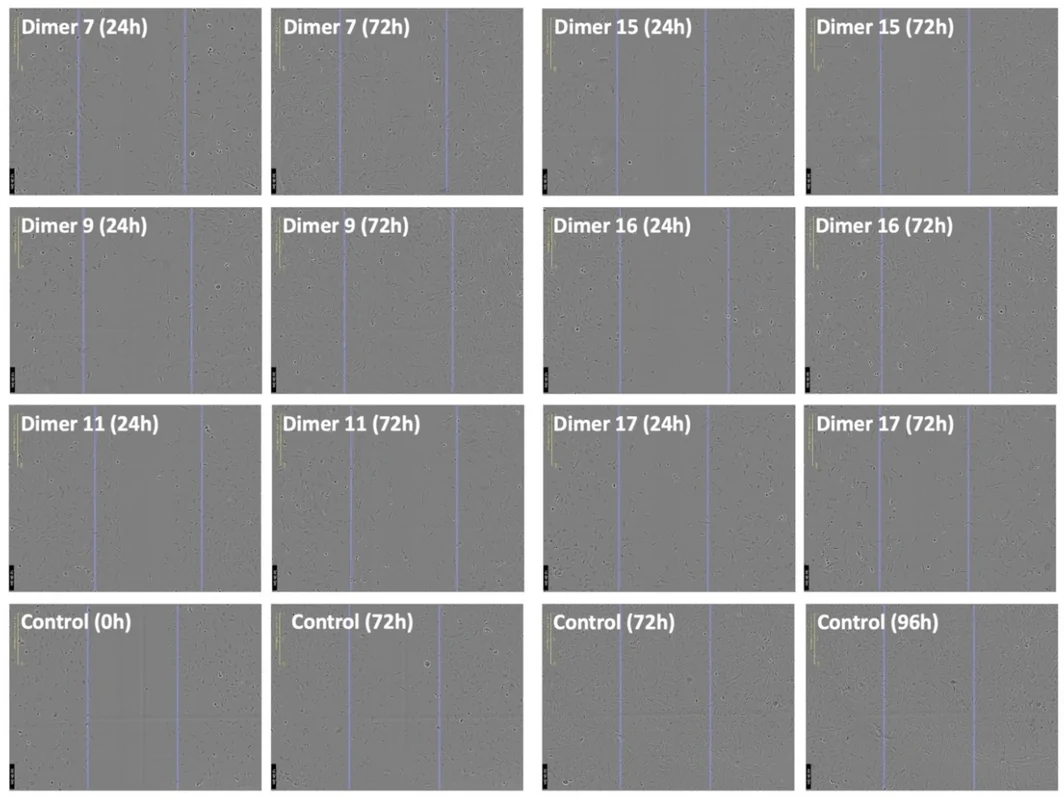
Representative IncuCyte images illustrating the effect of selected nucleoside dimers on the migration of U‐251 MG glioblastoma cells. Cells were treated with 50 μM of dimers **7**, **9**, **11**, **15**, **16**, and **17**, and wound closure was monitored using the IncuCyte Live‐Cell Analysis System for 96 h. Representative phase‐contrast images acquired after 24 and 72 h of treatment are shown for each dimer. Untreated control cells are presented at 0, 24, 72, and 96 h, demonstrating the progressive closure of the wound under normal growth conditions. In contrast, treatment with the selected dimers delayed wound closure to varying extents, with the most pronounced inhibition observed for dimers **7**, **11**, and **15**, which retained visibly wider wound areas after 72 h compared with the untreated control.

Following 72 h of incubation, untreated cells exhibited almost complete wound closure (91.74%). At this time point, treatment with 50 μM of dimers **7**, **11**, **15**, **16**, and **17** significantly suppressed cell migration, whereas dimer **9** no longer produced a statistically significant effect despite a slight reduction in wound closure. The most pronounced inhibition was observed for dimer **11**, which reduced the relative wound density to 45.68%, closely followed by dimer **15** (46.02%) and dimer **7** (52.95%). Dimers **16** and **17** exerted a more moderate inhibitory effect, yielding relative wound densities of 70.61% and 75.43%, respectively, whereas dimer **9** reached 85.77%, remaining close to the untreated control.

Overall, the IncuCyte migration assay demonstrated that the selected nucleoside dimers inhibit glioblastoma cell migration in a concentration‐dependent manner, with the strongest and most sustained anti‐migratory activity observed for dimers **7**, **11**, and **15**. These findings indicate that, in addition to their antiproliferative activity, selected dimers are capable of suppressing the migratory behavior of U‐251 MG cells, suggesting a potential role in limiting glioblastoma progression.

Since wound closure may be influenced by both cell migration and proliferation, the observed inhibitory effect likely reflects the combined consequence of reduced migratory capacity and the antiproliferative activity of the investigated dimers. Nevertheless, the persistent inhibition of wound closure observed throughout the 96 h experiment indicates that these compounds effectively impair the regenerative potential of U‐251 MG cells.

##### 2.3.3.5 Cell Cycle Analysis

To investigate whether the antiproliferative activity of the selected nucleoside dimers is associated with alterations in cell cycle progression, the distribution of U‐251 MG glioblastoma cells in the G_1_, S, and G_2_/M phases was analyzed by flow cytometry following treatment with dimers **7**, **9**, **11**, **15**, **16**, and **17** at concentrations of 10 and 50 μM. 5‐Fluoro‐2′‐deoxyuridine (5FdU, 10 μM) was included as the reference compound (Figure 10). Untreated control cells exhibited a typical cell cycle distribution, with 68.53% of cells in the G_1_ phase, 16.71% in the S phase, and 13.52% in the G_2_/M phase. As expected, treatment with 5FdU markedly altered cell cycle progression by inducing a pronounced S‐phase arrest. The percentage of cells in the S phase increased from 16.71% in the control to 33.73%, while the G_1_ and G_2_/M populations decreased to 63.04% and 3.25%, respectively. This pattern is consistent with the well‐established mechanism of action of 5FdU, which inhibits thymidylate synthase, leading to impaired DNA synthesis and accumulation of cells in the DNA replication phase. Similarly, treatment with all investigated dimers resulted in a concentration‐dependent accumulation of cells in the S phase, accompanied by a reduction in the proportion of cells in the G_1_ phase. This effect was particularly pronounced at 50 μM. Among the tested compounds, dimer **11** induced the strongest S‐phase arrest, increasing the S‐phase population from 16.71% in the control to 33.88%, while simultaneously reducing the G_1_ and G_2_/M populations to 60.52% and 4.60%, respectively. Notably, the cell cycle profile induced by dimer **11** closely resembled that observed for the reference compound 5FdU, suggesting a similar effect on DNA synthesis. A similar, although less pronounced, response was observed for dimer **7**, which increased the S‐phase fraction to 28.55%, as well as for dimers **9** and **15**, which increased the proportion of S‐phase cells to 26.21% and 28.54%, respectively. Dimers **16** and **17** also promoted S‐phase accumulation, although to a lesser extent. At 50 μM, the S‐phase population increased to 22.22% and 24.16%, respectively, compared with 16.71% in untreated cells. In parallel, treatment with dimers **7**, **9**, and **11** resulted in a marked reduction of the G_2_/M population, indicating impaired progression from DNA replication to mitosis, whereas dimers **15**, **16**, and **17** produced only moderate changes in the G_2_/M fraction.

**FIGURE 10:**
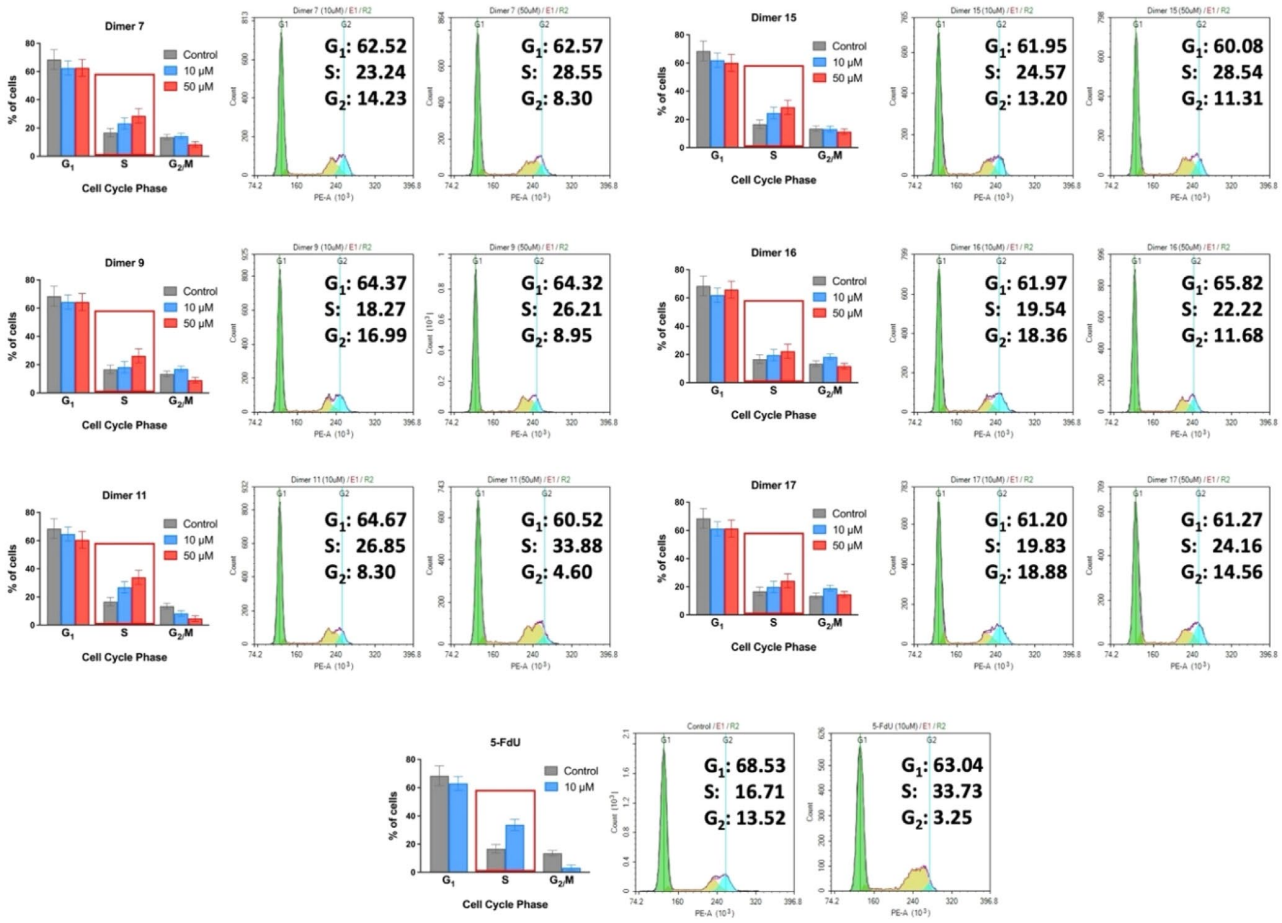
Effect of selected nucleoside dimers on cell cycle progression in U‐251 MG glioblastoma cells. Cells were treated with dimers **7**, **9**, **11**, **15**, **16**, and **17** at concentrations of 10 and 50 μM. 5‐Fluoro‐2′‐deoxyuridine (5FdU, 10 μM) was used as the reference compound. After treatment, cell cycle distribution was determined by flow cytometry following DNA staining. Representative DNA content histograms together with the quantitative distribution of cells in the G_1_, S, and G_2_/M phases are shown. Treatment with the selected dimers resulted in a concentration‐dependent accumulation of cells in the S phase, accompanied by a reduction in the G_1_ and/or G_2_/M populations, indicating inhibition of DNA synthesis and cell cycle progression. The most pronounced S‐phase arrest was observed for dimer **11** (50 μM), exhibiting a cell cycle profile comparable to that induced by the reference compound 5FdU. Data are presented as the mean ± SD of three independent experiments. Representative histograms are shown for each treatment condition.

Overall, the flow cytometric analysis demonstrates that the selected nucleoside dimers interfere with normal cell cycle progression by promoting S‐phase accumulation in U‐251 MG glioblastoma cells. The similarity between the cell cycle profiles induced by 5FdU and dimer **11**, and to a lesser extent dimers **7**, **9**, and **15**, supports the hypothesis that these compounds exert their antiproliferative effects through disruption of DNA synthesis, consistent with the expected mechanism of fluoropyrimidine‐based nucleoside analogues targeting thymidylate synthase.

#### 2.3.4 Selectivity Index

The selectivity index (SI), calculated as the ratio of the IC_50_ value in the non‐malignant MRC‐5 fibroblasts to the IC_50_ value in the respective cancer cell line, was used to evaluate the therapeutic selectivity of the synthesized compounds (Table 8). An SI value > 1 indicates preferential cytotoxicity toward cancer cells, while values exceeding 2 are generally considered indicative of meaningful selectivity.

**TABLE 8:** SI_50_ – the ratio of IC_50_ for MRC‐5 to IC_50_ for a given cell line.

	U‐138 MG	U‐251 MG	T47D	HeLa	SK‐OV‐3
5FdU (**19c**)	10.2	6.5	18.9	2	7.8
AddU (**21a**)	0.3	0.5	2.5	0.3	1.5
AFddU (**21c**)	1	7.5	1.2	2.3	1
4′‐ethynyl‐5′‐deoxy‐5′‐nor‐dU (**28a**)	1.7	2.1	2	1	1
4′‐ethynyl‐5′‐deoxy‐5′‐nor‐Thd (**28b**)	1	5.2	2	3.9	1.1
4′‐ethynyl‐5′‐deoxy‐5′‐nor‐5FdU (**28c**)	1	8.2	4.3	2.3	7
3′‐TBDMS‐4′‐ethynyl‐5′‐deoxy‐5′‐nor‐dU (**27a**)	1	4.5	3.7	5.9	1.3
3′‐TBDMS‐4′‐ethynyl‐5′‐deoxy‐5′‐nor‐Thd (**27b**)	1.3	5.6	4.1	5.7	1.2
3′‐TBDMS‐4′‐ethynyl‐5′‐deoxy‐5′‐nor‐5FdU (**27c**)	0.6	5	5.7	1.4	1.8
Dimer **1**	2	1.9	1.7	1	1.9
Dimer **2**	1.4	1.7	1.4	1.2	1.1
Dimer **3**	2.4	2.1	3.1	1.7	1
Dimer **4**	1.7	3.9	4.1	22.5	1.4
Dimer **5**	4.9	3.5	5	2.6	1.5
Dimer **6**	1.8	4.1	2.1	1.2	1
Dimer **7**	2.5	16.9	8.7	4.3	2.3
Dimer **8**	1.2	2.3	1.3	2.4	1
Dimer **9**	1.8	5.2	4.1	24.1	13.2
Dimer **10**	1.3	4.3	1.9	1.1	1.5
Dimer **11**	3.4	7.1	3.7	31.7	7.4
Dimer **12**	1.3	48.8	2.2	5.3	15.4
Dimer **13**	1.7	1.7	1.3	1.1	1.3
Dimer **14**	1.1	1.3	1.2	1.2	1.5
Dimer **15**	3.6	42.6	6.1	5.1	1.9
Dimer **16**	1.6	4.5	7.9	7.8	3.5
Dimer **17**	2.1	4.1	5.1	31.7	6.5
Dimer **18**	2.1	1	3.7	2.9	1

Among the reference compounds, 5‐fluoro‐2′‐deoxyuridine (5FdU) exhibited the highest selectivity, with SI values ranging from 2.0 to 18.9, confirming its well‐established tumor‐selective activity. In contrast, the synthesized nucleoside dimers generally displayed moderate selectivity. Most dimers showed SI values close to 1–3 against U‐138 MG and SK‐OV‐3 cells, indicating comparable cytotoxicity toward cancer and non‐malignant cells. However, several dimers demonstrated markedly improved selectivity toward specific cancer cell lines. In particular, dimers **4**, **9**, **11**, and **17** exhibited exceptionally high selectivity against HeLa cells, with SI values of 22.5, 24.1, 31.7, and 31.7, respectively. Likewise, dimers **12** and **15** showed remarkable selectivity toward U‐251 MG glioblastoma cells, with SI values of 48.8 and 42.6, respectively. Enhanced selectivity was also observed for dimer **9** against SK‐OV‐3 ovarian cancer cells (SI = 13.2) and dimer **12** (SI = 15.4). Moderate selectivity toward T47D breast cancer cells was observed for dimers **7** (SI = 8.7), **15** (SI = 6.1), **16** (SI = 7.9), and **17** (SI = 5.1).

Overall, although the dimers were generally less potent than 5FdU, several derivatives exhibited pronounced tumor selectivity toward individual cancer cell lines. These findings suggest that dimerization does not universally improve cytotoxic potency but may confer enhanced selectivity depending on the tumor type, making selected dimers attractive candidates for further mechanistic studies.

The marked differences in cytotoxic activity observed among the tested cancer cell lines most likely reflect their intrinsic molecular heterogeneity rather than their tissue of origin. Cancer cell lines differ substantially in the expression of equilibrative and concentrative nucleoside transporters (ENTs and CNTs), nucleoside‐metabolizing enzymes, proliferation rate, DNA repair capacity, and apoptotic signaling pathways, all of which are known to influence the biological activity of nucleoside analogues. In addition, the synthesized dimers possess considerably higher molecular weights and polar surface areas than the corresponding monomeric nucleosides, which may further increase their dependence on cell‐specific uptake mechanisms. Consequently, compounds such as dimers **4**, **9**, **11**, and **17** displayed pronounced activity toward HeLa cells, whereas dimers **7**, **12**, and **15** were markedly more selective toward U‐251 MG cells. Conversely, several cancer cell lines exhibited sensitivities comparable to those of the MRC‐5 pseudonormal fibroblasts, suggesting that these cells may lack the molecular determinants required for efficient uptake or intracellular activation of the dimers. Because only one representative cell line was investigated for most tumor types, these findings should be interpreted as cell line‐specific rather than tissue‐specific. Future studies using larger panels of molecularly characterized cell lines will be necessary to identify the determinants responsible for the differential sensitivity of individual tumors.

## 3 Conclusion

Despite well‐established clinical relevance, nucleoside analogues such as 5‐fluoro‐2′‐deoxyuridine suffer from metabolic liabilities, off‐target toxicity, and limited pharmacokinetic performance, which collectively restrict their therapeutic window. These limitations have stimulated medicinal chemistry efforts focused on structural modification of nucleoside scaffolds to enhance stability, selectivity, and overall anticancer efficacy. In particular, modification of internucleoside architectures using chemically robust linkers has emerged as an attractive strategy to generate novel nucleoside chemotypes with altered biological profiles. Among the available synthetic approaches, copper(I)‐catalyzed azide–alkyne cycloaddition (CuAAC) offers a powerful and versatile tool for the construction of triazole‐linked nucleoside conjugates, enabling systematic exploration of structure–activity relationships within this pharmacologically relevant class of compounds (Kondengadan et al. 2023).

In this study, we designed and synthesized a series of novel pyrimidine nucleoside dimers connected through an ultrashort 1,4‐disubstituted 1,2,3‐triazole linker using the Cu(I)‐catalyzed azide–alkyne cycloaddition (CuAAC) reaction. The synthesized compounds were fully characterized by multinuclear NMR spectroscopy and ESI‐MS and subsequently evaluated for their physicochemical properties, stability, and anticancer activity. Biological evaluation demonstrated that the triazole‐linked dimeric architecture substantially influenced antiproliferative activity in a cell line‐dependent manner. Dimers **4**, **9**, **11**, and **17** exhibited the highest activity against HeLa cervical cancer cells, whereas dimers **7** and **15** showed superior activity toward U‐251 MG glioblastoma cells, surpassing the reference compound 5‐fluoro‐2′‐deoxyuridine (5FdU). Importantly, the most active dimers displayed favorable selectivity toward cancer cells relative to MRC‐5 fibroblasts. Mechanistic studies revealed that the selected dimers markedly reduced long‐term clonogenic survival and inhibited glioblastoma cell migration while inducing concentration‐dependent cell death. Flow cytometric analysis demonstrated a pronounced accumulation of cells in the S phase, closely resembling the effect of 5FdU and supporting inhibition of DNA synthesis as the principal mechanism of action.

In contrast, only a modest and transient increase in intracellular reactive oxygen species was observed, indicating that oxidative stress is unlikely to represent the primary driver of their cytotoxic activity. In addition to their biological activity, the synthesized dimers exhibited excellent chemical and metabolic stability in physiological media. Combined with their predicted resistance to enzymatic degradation, these properties distinguish them from conventional nucleoside analogues. Although their physicochemical characteristics suggest limited oral bioavailability and a need for parenteral administration, their exceptional stability makes them attractive not only as leads for the development of novel anticancer agents but also as durable nucleoside building blocks for nucleic acid‐based applications, including highly stable primers, probes, and other diagnostic tools. Overall, this study demonstrates that rigid triazole‐linked pyrimidine nucleoside dimers constitute a promising scaffold with favorable stability, selective anticancer activity, and a mechanism of action consistent with disruption of DNA synthesis. The obtained results provide a strong foundation for further structural optimization and evaluation of these compounds in both therapeutic and nucleic acid‐targeting applications. Beyond their direct therapeutic potential, the high chemical stability and nuclease resistance imparted by the triazole‐modified backbone suggest broader applicability of these nucleoside dimers as functional building blocks in nucleic acid chemistry. In particular, such modified DNA architectures may be exploited for the design of oligonucleotide probes, the assembly of synthetic genes, and the site‐specific incorporation of chemically modified nucleobases or sugars, as well as for the preparation of engineered RNA constructs for functional and mechanistic studies (El‐Sagheer and Brown 2012, 2015). Collectively, the present results establish triazole‐linked nucleoside dimers as a versatile medicinal chemistry platform with potential relevance to both anticancer drug development and nucleic acid–based technologies.

## 4 Experimental Protocols

### 4.1 Chemistry

Chemical reagents were purchased from Acros Organics, Alfa Aesar, Carbosynth, and Sigma‐Aldrich. Thin layer chromatography (TLC) was performed on silica gel 60 F254 precoated (0.2 mm) plates and vacuum flash column chromatography on silica gel 60 H (5–40 μm), purchased from Merck.

**3′‐azido pyrimidine nucleosides**

*Procedure for the synthesis of 2,3′‐anhydro‐2′‐deoxyuridine* (***20**, 2,3′‐anhydro‐dU*)

2′‐Deoxyuridine (dU, 1 mmol, 1 eq) was placed in a round‐bottomed flask and dissolved in ACN (12.2 mL), followed by addition of PPh_3_ (2 mmol). The whole mixture was dissolved and cooled to −15°C in a methanol‐ice bath. Then DEAD (2 mmol) was slowly added dropwise, so that the temperature did not exceed −5°C. TLC analysis (DCM/MeOH, 3:1, v/v) showed that the reaction was completed after 1 h. The reaction mixture was warmed to room temperature, so stirring was continued for an additional hour. The solvents were evaporated and the residue was purified by column chromatography on silica gel using DCM/MeOH (from 9:1 to 7:3, v/v) as an eluent. The yield of the anhydro‐nucleoside reaction was about 88%.

*Procedure for the synthesis of 3′‐azido‐2′,3′‐dideoxyuridine* (***21a**, AddU*)

In a round‐bottomed flask equipped with a reflux condenser with a CaCl_2_ tube, 2,3′‐anhydro‐2′‐deoxyuridine (1 mmol, 1 eq) was placed and dissolved in DMF (10 mL). Then, NaN_3_ (2 eqs) and *p*‐TsOHxH_2_O (1 eq) were added, and the whole was heated in an oil bath at 120°C for 3 h. After the reaction was completed (TLC: DCM/MeOH, 10:1, v/v), the mixture was evaporated to dryness, and the crude product was purified by column chromatography on silica gel using AcOEt or DCM/MeOH (from 95:5 to 90:10, v/v) as eluents. The compound AddU was obtained with a yield about 65%. ^1^H NMR *δ*: 11.32 (brs, 1H, H3), 7.83 (d, ^3^ *J*_HH_ = 8.11 Hz, 1H, H6), 6.07 (t, ^3^ *J*_HH_ = 6.28 Hz, 6.30 Hz, 1H, H1′), 5.63 (d, ^3^ *J*_HH_ = 8.11 Hz, 1H, H5), 5.20 (t, ^3^ *J*_HH_ = 5.19 Hz, 5.15 Hz, 1H, 5′OH), 4.37 (q, ^3^ *J*_HH_ = 5.19 Hz, 6.23 Hz, 5.32 Hz, 1H, H3′), 3.83 (q, ^3^ *J*_HH_ = 3.66 Hz, 4.10 Hz, 3.79 Hz, 1H, H4′), 3.65–3.56 (m, 2H, H5′, H5″), 2.40–2.28 (m, 2H, H2′, H2″). ^13^C NMR *δ*: 163.1 (C4), 150.4 (C2), 140.5 (C6), 101.8 (C5), 84.16 (C4′), 83.63 (C1′), 60.73 (C5′), 60.12 (C3′), 36.44 (C2′).

**3′‐TBDMS‐4′‐ethynyl‐5′‐deoxy‐5′‐nor‐pyrimidine nucleosides of 2′‐deoxyuridine, thymidine, and 5‐fluoro‐2′‐deoxyuridine (27a, 27b, 27c) and 4′‐ethynyl‐5′‐deoxy‐5′‐nor‐pyrimidine nucleosides of 2′‐deoxyuridine, thymidine, and 5‐fluoro‐2′‐deoxyuridine (28a, 28b, 28c)**

*General procedure for the synthesis of 5′‐O‐dimethoxytrityl‐pyrimidine nucleosides of type **22a*** **–*c***

Nucleoside (1 mmol, 1 eq) was dissolved in anhydrous pyridine (Py, *ca*. 2.4 mL) and dimethoxytrityl chloride (DMTr‐Cl, 1.05 eq) was added. The mixture was stirred on a magnetic stirrer at room temperature for 3 h, and then the reaction was monitored by TLC (DCM/MeOH, 9:1, v/v). Methanol (1 mL) was added to quench the reaction and it was evaporated to dryness. Then toluene was added and co‐evaporated to remove pyridine residues. Next, it was dissolved in DCM and extracted with sat. NaHCO_3_ solution. The organic layer was dried over anhydrous MgSO_4_, filtered, and evaporated to foam under reduced pressure. Add toluene and evaporate to remove any remaining pyridine. The residue was purified by column chromatography on silica gel using DCM/MeOH (from 98:2, 95:5 to 90:10, v/v) as an eluent. The reaction products were obtained with a yield of about 90%. In the case of 5′‐DMTr‐thymidine, this compound can be purified by precipitation from toluene. Dissolve 5′‐DMTr‐Thd in a small amount of CH_2_Cl_2_ and add about 10 mL of toluene, concentrate on an evaporator, and leave to crystallize.

*General procedure for the synthesis of 3′‐O‐tert‐butyldimethylsilyl‐5′‐O‐dimethoxytrityl‐pyrimidine nucleosides of type **23a*** **–*c***

5′‐*O*‐DMTr‐nucleoside **22a–c** (1 mmol, 1 eq) and imidazole (2.5 eqs) were co‐evaporated twice with anhydrous pyridine. Then pyridine (6 mL) and *tert*‐butyldimethylsilyl chloride (TBDMS‐Cl, 1.2 eq) were added. The reaction was carried out on a magnetic stirrer at room temperature and under argon atmosphere overnight. After TLC control (DCM/MeOH, 9:1, v/v), it was evaporated to dryness and then co‐evaporated twice with toluene. The residue was dissolved in DCM and extracted with sat. NaHCO_3_ solution. The combined organic layers were evaporated to dryness, and the obtained white oil was purified by column chromatography on silica gel using DCM/MeOH (from 98:2 to 95:5, v/v) as an eluent. The sililated product was obtained with 100% yield.

*General procedure for the synthesis of 3′‐O‐tert‐butyldimethylsilyl‐pyrimidine nucleosides of type **24a*** **–*c***

To the 5′‐*O*‐DMTr‐3′‐*O*‐TBDMS‐nucleoside **23a–c** (1 mmol, 1 eq) was added 5% solution of *p*‐TsOHx5H_2_O (5 eqs) in DCM/MeOH (9:1, v/v). The reaction was carried out for 15 min on a magnetic stirrer (TLC: DCM/MeOH, 20:1, v/v), which was terminated by the addition of sat. NaHCO_3_ solution (20 mL) and next DCM (3 × 10 mL). After phase separation, the organic phase was evaporated with silica gel, and the residue was purified by column chromatography on silica gel using DCM/MeOH (from 98:2, 95:5 to 90:10, v/v) as an eluent. The 3′‐*O*‐TBDMS‐products were obtained in the range of 40%–70% yield.

*General procedure for the synthesis of 3′‐O‐tert‐butyldimethylsilyl‐5′‐aldehyde‐pyrimidine nucleosides of type **25a*** **–*c***

3′‐*O*‐(*tert*‐butyldiphenylsilyl)nucleoside **24a–c** (1 mmol, 1 eq) was dissolved in anhydrous AcOEt (7 mL), and s‐IBX (2.2 eqs) was added. The suspension was immersed in an oil bath set to 80°C and stirred vigorously open to the atmosphere using an air cooler. After 3.5 h, the reaction mixture was cooled to room temperature and filtered. The precipitate was washed with 2 × 5 mL of AcOEt, diluted with AcOEt, and extracted with sat. NaHCO_3_ solution. After evaporation of the organic phase, the product was dried under oil pump and stored in a freezer. The desired aldehyde **25a–c** was used without purification for the next step. Experimental notes: AcOEt was dried over K_2_CO_3_, distilled over P_2_O_5_, and stored over 4 Å molecular sieves.

*General procedure for the synthesis of 3′‐O‐tert‐butyldimethylsilyl‐5′‐*(*di‐bromo‐vinyl*)*‐pyrimidine nucleosides of type **26a*** **–*c***

Nucleoside **25a–c** (1 mmol, 1 eq) was dissolved in anhydrous dioxane (16 mL), then ylide^1^ (Ph_3_CHBr_2_^+^Br^−^, 2 eqs) and zinc (2.2 eqs) were added. The suspension was heated under reflux (*ca*. 125°C) for 3 h, then cooled to room temperature and filtered. The filtrate was evaporated, dissolved in DCM (28 mL) and extracted with brine (17 mL). The organic layer was separated, dried over MgSO_4_ and concentrated. The crude product was purified by column chromatography on silica gel using DCM/MeOH (98:2, v/v) as an eluent, and next in *n*‐hexane/AcOEt (2:1, v/v). The product was obtained in about 70% yield.

*Preparation of dibromomethyltriphenylphosphonium bromide*

Carbon tetrabromide (CBr_4_, 0.33 g, 1 mmol, 1 eq) was added to a solution of triphenylphosphine (Ph_3_P, 0.52 g, 2 mmol, 2 eqs) in DCM (4.85 mL). The solution was stirred at room temperature for 15 min. Water (0.16 mL) was added to this red reaction mixture. After 15 min of vigorous magnetic stirring, the aqueous layer was separated from DCM. The organic layer was dried with anhydrous MgSO_4_, then evaporated at reduced pressure and stored in a desiccator over P_2_O_5_. Next, the salt was crystallized in anhydrous ACN at reflux, then filtered hot under vacuum, dried under vacuum over P_2_O_5_, and stored at −20°C under an argon atmosphere (yield 80%). Experimental notes: (a) CBr_4_ and PPh_3_‐ dried in a desiccator over P_2_O_5_, (b) ACN and DCM − dried over CaCl_2_, distilled over P_2_O_5_ and stored over 4 Å molecular sieves.

*General procedure for the synthesis of 3′‐O‐tert‐butyldimethylsilyl‐4′‐ethynyl‐5′‐deoxy‐5′‐nor‐pyrimidine nucleosides of type **27a*** **–*c***

The nucleoside substrate **26a–c** (1 mmol, 1 eq) was dissolved in anhydrous THF (7.4 mL), and then the mixture was cooled to −78°C in a cooling bath of dry ice and isopropanol under an argon atmosphere. Then 1.6 M *n*‐BuLi in hexane (2.62 mL, 4.19 mmol) was added dropwise. The reaction mixture was stirred vigorously for 45 min, and then warmed to room temperature (TLC: toluene/MeOH, 6:1, v/v). 13 mL of sat. NH_4_Cl solution was added and extracted with DCM (3 × 13 mL). Combined organic layers were washed with H_2_O (22 mL), dried over MgSO_4_ and evaporated with silica gel to dryness. The residue was purified by column chromatography on silica gel using DCM/MeOH (99:1, v/v) or *n*‐hexane/AcOEt (2:1, v/v) as eluents.

3′‐TBDMS‐4′‐ethynyl‐5′‐deoxy‐5′‐nor‐dU (**27a**): ^1^H NMR *δ*: 11.35 (brs, 1H), 7.60 (d, ^3^ *J*_HH_ = 8.1 Hz, 1H, H6), 6.18 (t, ^3^ *J*_HH_ = 6.8 Hz, 6.9 Hz, 1H, H1′), 5.72 (d, ^3^ *J*_HH_ = 8.1 Hz, 1H, H5), 4.55 (m, 1H, H3′), 4.47 (t, ^3^ *J*_HH_ = 2.6 Hz, ^4^ *J*_HH_ = 2.5 Hz, 1H, H4′), 3.82 (d, ^4^ *J*_HH_ = 2.2 Hz, 1H, H6′), 2.34–2.29 (m, 1H, H2′), 2.24–2.20 (m, 1H, H2″), 0.88 (s, 9H, CH_3_), 0.11 (s, 6H, CH_3_). ^13^C NMR *δ*: 163.0 (C4), 150.4 (C2), 140.2 (C6), 102.3 (C5), 85.09 (C1′), 80.66 (C5′), 79.16 (C6′), 76.74 (C3′), 75.39 (C4′), 38.55 (C2′), 25.62 (CH_3_), 17.70 (C‐tBu), −4.99 (CH_3_Si). ^15^N NMR *δ*: −145.7 (N1). ^29^Si NMR *δ*: 21.15. HRMS (ESI‐) calcd for C_16_H_23_N_2_O_4_Si [M − H]^−^ 335.1427, found 335.1448.

3′‐TBDMS‐4′‐ethynyl‐5′‐deoxy‐5′‐nor‐Thd (**27b**): ^1^H NMR *δ*: 11.36 (brs, 1H), 7.49 (d, ^4^ *J*_HH_ = 1.2 Hz, 1H, H6), 6.21 (t, ^3^ *J*_HH_ = 7.0 Hz, 7.1 Hz, 1H, H1′), 4.58–4.55 (m, 1H, H3′), 4.45 (t, ^3^ *J*_HH_ = 2.4 Hz, ^4^ *J*_HH_ = 2.5 Hz, 1H, H4′), 3.86 (d, ^4^ *J*_HH_ = 2.2 Hz, 1H, H6′), 2.37–2.31 (m, 1H, H2′), 2.21–2.15 (m, 1H, H2″), 1.80 (d, ^4^ *J*_HH_ = 1.2 Hz, 3H, ^5^CH_3_), 0.88 (s, 9H, CH_3_), 0.11 (s, 6H, CH_3_). ^13^C NMR *δ*: 163.6 (C4), 150.4 (C2), 135.8 (C6), 109.9 (C5), 84.77 (C1′), 80.83 (C5′), 79.22 (C6′), 76.82 (C3′), 75.32 (C4′), 38.40 (C2′), 25.61 (CH_3_), 17.69 (C‐tBu), 12.33 (^5^CH_3_), −4.98 (CH_3_Si), −4.99 (CH_3_Si). ^15^N NMR *δ*: −142.4 (N1). ^29^Si NMR *δ*: 21.06. HRMS (ESI‐) calcd for C_17_H_25_N_2_O_4_Si [M − H]^−^ 349.1583, found 349.1605. HRMS (ESI+) calcd for C_17_H_27_N_2_O_4_Si [M + H]^+^ 351.1740, found 351.1729.

3′‐TBDMS‐4′‐ethynyl‐5′‐deoxy‐5′‐nor‐5FdU (**27c**): ^1^H NMR *δ*: 11.90 (brs, 1H), 7.83 (d, ^3^ *J*_HF_ = 6.9 Hz, 1H, H6), 6.17 (t, ^3^ *J*_HH_ = 6.7 Hz, 6.6 Hz, 1H, H1′), 4.59–4.56 (m, 1H, H3′), 4.47 (t, ^3^ *J*_HH_ = 2.4 Hz, ^4^ *J*_HH_ = 2.6 Hz, 1H, H4′), 3.89 (d, ^4^ *J*_HH_ = 2.1 Hz, 1H, H6′), 2.41–2.34 (m, 1H, H2′), 2.24–2.18 (m, 1H, H2″), 0.87 (s, 9H, CH_3_), 0.11 (s, 6H, CH_3_). ^13^C NMR *δ*: 157.0 (d, ^2^ *J*_CF_ = 26 Hz, C4), 149.0 (C2), 140.1 (d, ^1^ *J*_CF_ = 231 Hz, C5), 124.4 (d, ^2^ *J*_CF_ = 34 Hz, C6), 85.49 (C1′), 80.64 (C5′), 79.35 (C6′), 76.54 (C3′), 75.41 (C4′), 38.88 (C2′), 25.61 (CH_3_), 17.68 (C‐tBu), −4.98 (CH_3_Si), −4.99 (CH_3_Si). ^15^N NMR *δ*: −134.6 (N1), −158.1 (N3). ^19^F NMR *δ*: −166.4. ^29^Si NMR *δ*: 21.16. HRMS (ESI‐) calcd for C_16_H_22_FN_2_O_4_Si [M − H]^−^ 353.1332, found 353.1352.

*General procedure for the synthesis of 4′‐ethynyl‐5′‐deoxy‐5′‐nor‐pyrimidine nucleosides of type **28a*** **–*c***

A solution of the 3′‐TBDMS‐4′‐ethynyl‐5′‐deoxy‐5′‐nor‐nucleoside **27a–c** (1 mmol, 1 eq) and NH_4_F (5 eqs) in anhydrous MeOH (18 mL) was heated at reflux for 4 h. The reaction was monitored by TLC (toluene/MeOH, 3:1, v/v), and finally, silica gel was added to the mixture. Everything was evaporated to dryness, and the residue was purified by column chromatography on silica gel using DCM/MeOH (95:5, v/v) as an eluent.

4′‐ethynyl‐5′‐deoxy‐5′‐nor‐dU (**28a**): ^1^H NMR *δ*: 11.37 (brs, 1H), 7.60 (d, ^3^ *J*_HH_ = 8.1 Hz, 1H, H6), 6.23 (t, ^3^ *J*_HH_ = 6.4 Hz, 7.61 Hz, 1H, H1′), 5.77–5.75 (m, 1H, 3′OH), 5.73–5.71 (dd, ^3^ *J*_HH_ = 8.1, ^4^ *J*_HH_ = 2.1 Hz 1H, H5), 4.51 (t, ^3^ *J*_HH_ = 2.0 Hz, ^4^ *J*_HH_ = 2.0 Hz, 1H, H4′), 4.39–4.37 (m, 1H, H3′), 3.79 (d, ^4^ *J*_HH_ = 2.1 Hz, 1H, H6′), 2.28–2.23 (m, 1H, H2′), 2.22–2.17 (m, 1H, H2″). ^13^C NMR *δ*: 163.09 (C4), 150.46 (C2), 139.93 (C6), 102.35 (C5), 85.60 (C1′), 81.23 (C5′), 79.16 (C6′), 75.90 (C4′), 75.60 (C3′), 38.76 (C2′). ^15^N NMR *δ*: −145.6 (N1), −158.2 (N3). HRMS (ESI‐) calcd for C_10_H_9_N_2_O_4_ [M − H]^−^ 221.0562, found 221.0562.

4′‐ethynyl‐5′‐deoxy‐5′‐nor‐Thd (**28b**): ^1^H NMR *δ*: 11.34 (brs, 1H), 7.50 (d, ^4^ *J*_HH_ = 1.2 Hz, 1H, H6), 6.27 (t, ^3^ *J*_HH_ = 6.9 Hz, 7.4 Hz, 1H, H1′), 5.72 (brs, 1H, 3′OH), 4.49 (t, ^3^ *J*_HH_ = 2.0 Hz, ^4^ *J*_HH_ = 2.0 Hz, 1H, H4′), 4.39 (brs, 1H, H3′), 3.85 (d, ^4^ *J*_HH_ = 2.1 Hz, 1H, H6′), 2.24–2.21 (m, 2H, H2′, H2″), 1.79 (d, ^4^ *J*_HH_ = 1.2 Hz, 3H, ^5^CH_3_). ^13^C NMR *δ*: 163.6 (C4), 150.5 (C2), 135.6 (C6), 109.9 (C5), 85.20 (C1′), 81.41 (C5′), 79.17 (C6′), 75.76 (C4′), 75.60 (C3′), 38.43 (C2′), 12.38 (^5^CH_3_). ^15^N NMR *δ*: −142.8 (N1). HRMS (ESI‐) calcd for C_11_H_11_N_2_O_4_ [M − H]^−^ 235.0718, found 235.0729. HRMS (ESI+) calcd for C_11_H_13_N_2_O_4_ [M + H]^+^ 237.0875, found 237.0868.

4′‐ethynyl‐5′‐deoxy‐5′‐nor‐5FdU (**28c**): ^1^H NMR *δ*: 11.88 (brs, 1H), 7.79 (d, ^3^ *J*_HF_ = 6.9 Hz, 1H, H6), 6.22 (td, ^3^ *J*_HH_ = 6.6 Hz, 7.2 Hz, ^5^ *J*_HF_ = 1.3 Hz, 1H, H1′), 5.72 (brs, 1H, 3′OH), 4.52 (t, ^3^ *J*_HH_ = 1.9 Hz, ^4^ *J*_HH_ = 1.9 Hz, 1H, H4′), 4.39 (brs, 1H, H3′), 3.88 (d, ^4^ *J*_HH_ = 2.2 Hz, 1H, H6′), 2.31–2.22 (m, 2H, H2′, H2″). ^13^C NMR *δ*: 157.0 (d, ^2^ *J*_CF_ = 26 Hz, C4), 149.0 (C2), 140.1 (d, ^1^ *J*_CF_ = 231 Hz, C5), 124.2 (d, ^2^ *J*_CF_ = 34 Hz, C6), 86.09 (C1′), 81.18 (C5′), 79.41 (C6′), 75.96 (C4′), 75.46 (C3′), 38.54 (C2′). ^19^F NMR *δ*: −166.3. HRMS (ESI‐) calcd for C_10_H_8_FN_2_O_4_ [M − H]^−^ 239.0468, found 239.0476. HRMS (ESI+) calcd for C_10_H_10_FN_2_O_4_ [M + H]^+^ 241.0624, found 241.0620.

**Dimers 1–18**

In a round‐bottomed flask, nucleoside azide (AddU, AZT, or AFddU, 1 mmol) and an equimolar amount of 3′‐TBDMS‐4′‐ethynyl‐5′‐deoxy‐5′‐nor‐ or 4′‐ethynyl‐5′‐deoxy‐5′‐nor‐ (**27a–c** or **28a–c**, respectively; 1 mmol) were placed. The substrates were dissolved in THF/H_2_O (3:1, 16/5 mL, v/v) and stirred using a magnetic stirrer at room temperature until completely dissolved. Subsequently, NaAsc (0.8 eq) was added. The mixture was stirred until a homogenous solution was obtained. Finally 1 M CuSO_4_ solution (0.4 eq) was added and the flask was placed under a reflux condenser with heating. The reaction mixture was vigorously stirred at the boiling point of solvents (about 70°C) for 3 h. When the reaction was completed (TLC: (a) AcOEt or toluene/EtOH (6:1, v/v), (b) AcOEt/MeOH (4:1, v/v) or toluene/EtOH (3:1, v/v)), the mixture was evaporated with silica gel, and the product was isolated by column chromatography on silica gel using AcOEt/MeOH (from 10:0 to 8:2, v/v) as an eluent. The compound was finally purified by silica gel chromatography in toluene/EtOH ((a) 6:1, v/v; (b) 3:1, v/v). The products were obtained as white solids with yield in the range 70%–90% after chromatographic purification. The symbols used in chromatography mean: (a) for dimers with an odd number, that is, with 3′‐TBDMS blocking group, and (b) for dimers with an even number, that is, with 3′‐OH group.

**Dimer 1:** ^1^H NMR *δ*: 11.35 (brs, 2H, H3A, H3B), 8.42 (s, 1H, H5C), 7.97 (d, ^3^ *J*_HH_ = 8.2 Hz, 1H, H6A), 7.88 (d, ^3^ *J*_HH_ = 8.1 Hz, 1H, H6B), 6.40 (t, ^3^ *J*_HH_ = 6.6 Hz, 6.7 Hz, 1H, H1′A), 6.29 (t, ^3^ *J*_HH_ = 6.6 Hz, 6.6 Hz, 1H, H1′B), 5.69 (dd, ^3^ *J*_HH_ = 8.1 Hz, 8.1 Hz, 2H, H5A, H5B), 5.39–5.36 (m, 1H, H3′B), 5.31 (t, ^3^ *J*_HH_ = 5.1 Hz, 5.0 Hz, 1H, 5′A‐OH), 4.89 (d, ^3^ *J*_HH_ = 3.9 Hz, 1H, H4′B), 4.65–4.62 (m, 1H, H3′B), 4.25–4.22 (m, 1H, H4′A), 3.72–3.68 (m, 1H, H5′A), 3.64–3.60 (m, 1H, H5″A), 2.78–2.73 (m, 1H, H2′A), 2.68–2.62 (m, 1H, H2″A), 2.50–2.46 (m, 1H, H2′B), 2.31–2.26 (m, 1H, H2″B), 0.82 (s, 9H, CH_3_), 0.02 (s, 3H, CH_3_), −0.03 (s, 3H, CH_3_). ^13^C NMR *δ*: 163.1 (C4A, C4B), 150.4 (C2B, C2A), 145.3 (C4C), 140.7 (C6A), 140.6 (C6B), 123.5 (C5C), 102.2 (C5A), 102.0 (C5B), 84.67 (C4′A), 84.33 (C1′A, C1′B), 79.91 (C4′B), 75.67 (C3′B), 60.68 (C5′A), 59.39 (C3′A), 39.51 (C2′B), 37.38 (C2′A), 25.60 (CH_3_), 17.67 (C‐tBu), −4.97 (CH_3_Si), −5.04 (CH_3_Si). ^15^N NMR *δ*: −146.2 (N1B), −147.1 (N1A), −158.1 (N3A, N3B), −254.3 (N1C), −350.8 (N3C), −360.8 (N2C). ^29^Si NMR *δ*: 20.62. HRMS (ESI‐) calcd for C_25_H_34_N_7_O_8_Si [M − H]^−^ 588.2238, found 588.2240. HRMS (ESI+) calcd for C_25_H_36_N_7_O_8_Si [M + H]^+^ 590.2394, found 590.2381.

**Dimer 2:** ^1^H NMR *δ*: 11.36 (brs, 2H, H3A, H3B), 8.37 (s, 1H, H5C), 7.97 (d, ^3^ *J*_HH_ = 8.2 Hz, 1H, H6A), 7.85 (d, ^3^ *J*_HH_ = 8.1 Hz, 1H, H6B), 6.41 (t, ^3^ *J*_HH_ = 6.6 Hz, 6.6 Hz, 1H, H1′A), 6.32 (t, ^3^ *J*_HH_ = 6.7 Hz, 6.9 Hz, 1H, H1′B), 5.69 (t, ^3^ *J*_HH_ = 7.7 Hz, 7.6 Hz, 2H, H5A, H5B), 5.63 (d, ^3^ *J*_HH_ = 4.3 Hz, 1H, 3′B‐OH), 5.35 (m, 1H, H3′A), 5.29 (t, ^3^ *J*_HH_ = 5.1 Hz, 5.1 Hz, 1H, 5′A‐OH), 4.92 (d, ^3^ *J*_HH_ = 3.1 Hz, 1H, H4′B), 4.48–4.45 (m, 1H, H3′B), 4.26 (m, 1H, H4′A), 3.72–3.68 (m, 1H, H5′A), 3.66–3.61 (m, 1H, H5″A), 2.79–2.74 (m, 1H, H2′A), 2.68–2.62 (m, 1H, H2″A), 2.44–2.39 (m, 1H, H2′B), 2.31–2.26 (m, 1H, H2″B). ^13^C NMR *δ*: 163.1 (C4A), 163.0 (C4B), 150.5 (C2B), 150.4 (C2A), 145.8 (C4C), 140.7 (C6A), 140.4 (C6B), 123.3 (C5C), 102.1 (C5A), 102.0 (C5B), 84.64 (C4′A), 84.51 (C1′A), 84.37 (C1′B), 80.07 (C4′B), 74.08 (C3′B), 60.79 (C5′A), 59.45 (C3′A), 39.51 (C2′B), 37.37 (C2′A). ^15^N NMR *δ*: −146.0 (N1b), −146.8 (N1A), −158.1 (N3A/N3B), −254.1 (N1C), −350.5 (N3C), −360.6 (N2C). HRMS (ESI‐) calcd for C_19_H_20_N_7_O_8_ [M − H]^−^ 474.1373, found 474.1387.

**Dimer 3:** ^1^H NMR *δ*: 11.34 (brs, 2H, H3A, H3B), 8.41 (s, 1H, H5C), 7.96 (d, ^3^ *J*_HH_ = 8.1 Hz, 1H, H6A), 7.67 (d, ^4^ *J*_HH_ = 1.2 Hz, 1H, H6B), 6.40 (t, ^3^ *J*_HH_ = 6.4 Hz, 6.6 Hz, 1H, H1′A), 6.30 (t, ^3^ *J*_HH_ = 6.7 Hz, 6.8 Hz, 1H, H1′B), 5.69 (d, ^3^ *J*_HH_ = 8.1 Hz, 1H, H5A), 5.40–5.36 (m, 1H, H3′A), 5.29 (t, ^3^ *J*_HH_ = 5.1 Hz, 5.0 Hz, 1H, 5′A‐OH), 4.89 (d, ^3^ *J*_HH_ = 3.7 Hz, 1H, H4′B), 4.70–4.67 (m, 1H, H3′B), 4.26–4.23 (m, 1H, H4′A), 3.72–3.68 (m, 1H, H5′A), 3.63–3.59 (m, 1H, H5″A), 2.80–2.74 (m, 1H, H2′A), 2.69–2.63 (m, 1H, H2″A), 2.53–2.48 (m, 1H, H2′B), 2.26–2.22 (m, 1H, H2″B), 1.77 (d, ^4^ *J*_HH_ = 1.2 Hz, 3H, ^5B^CH_3_), 0.84 (s, 9H, CH_3_), 0.03 (s, 3H, CH_3_), −0.01 (s, 3H, CH_3_). ^13^C NMR *δ*: 163.7 (C4A), 163.1 (C4B), 150.5 (C2B), 150.4 (C2A), 145.4 (C4C), 140.7 (C6A), 136.2 (C6B), 123.4 (C5C), 109.7 (C5B), 101.9 (C5A), 84.65 (C4′A), 84.33 (C1′A), 84.20 (C1′B), 79.98 (C4′B), 75.70 (C3′B), 60.63 (C5′A), 59.32 (C3′A), 39.51 (C2′B), 37.38 (C2′A), 25.61 (CH_3_), 17.68 (C‐tBu), 12.33 (^5B^CH_3_), −4.94 (CH_3_Si). ^15^N NMR *δ*: −143.2 (N1B), −147.2 (N1A), −156.1 (N3A, N3B), −254.3 (N1C), −350.7 (N3C), −360.1 (N2C). ^29^Si NMR *δ*: 20.49. HRMS (ESI‐) calcd for C_26_H_36_N_7_O_8_Si [M − H]^−^ 602.2394, found 602.2396.

**Dimer 4:** ^1^H NMR *δ*: 11.34 (brs, 2H, H3A, H3B), 8.38 (s, 1H, H5C), 7.96 (d, ^3^ *J*_HH_ = 8.1 Hz, 1H, H6A), 7.65 (d, ^4^ *J*_HH_ = 1.2 Hz, 1H, H6B), 6.40 (t, ^3^ *J*_HH_ = 6.5 Hz, 6.6 Hz, 1H, H1′A), 6.33 (t, ^3^ *J*_HH_ = 6.6 Hz, 6.6 Hz, 1H, H1′B), 5.69 (d, ^3^ *J*_HH_ = 8.1 Hz, 1H, H5A), 5.62 (d, ^3^ *J*_HH_ = 4.2 Hz, 1H, 3′B‐OH), 5.38–5.36 (m, 1H, H3′A), 5.30 (t, ^3^ *J*_HH_ = 5.1 Hz, 5.0 Hz, 1H, 5′A‐OH), 4.91 (d, ^3^ *J*_HH_ = 3.6 Hz, 1H, H4′B), 4.52 (m, 1H, H3′B), 4.26 (m, 1H, H4′A), 3.72–3.68 (m, 1H, H5′A), 3.65–3.61 (m, 1H, H5″B), 2.80–2.75 (m, 1H, H2′A), 2.68–2.62 (m, 1H, H2″A), 2.46–2.40 (m, 1H, H2′B), 2.27–2.23 (m, 1H, H2″B), 1.75 (d, ^4^ *J*_HH_ = 1.2 Hz, 3H,^5B^CH_3_). ^13^C NMR *δ*: 163.6 (C4A), 163.1 (C4B), 150.5 (C2A), 150.4 (C2B), 145.9 (C4C), 140.7 (C6A), 136.1 (C6B), 123.3 (C5C), 109.6 (C5B), 101.9 (C5A), 84.63 (C4′A), 84.34 (C1′A), 84.25 (C1′B), 80.04 (C4′B), 74.00 (C3′B), 60.72 (C5′A), 59.35 (C3′A), 39.51 (C2′B), 37.38 (C2′A), 12.31 (^5B^ CH_3_). ^15^N NMR *δ*: −143.5 (N1B), −147.1 (N1A), −158.1 (N3A), −254.1 (N1C), −350.6 (N3C), −360.7 (N2C). HRMS (ESI‐) calcd for C_20_H_22_N_7_O_8_ [M − H]^−^ 488.1529, found 488.1539.

**Dimer 5:** ^1^H NMR *δ*: 11.85 (brs, 1H, H3B), 11.37 (brs, 1H, H3A), 8.44 (s, 1H, H5C), 8.24 (d, ^3^ *J*_HF_ = 7.1 Hz, 1H, H6B), 7.97 (d, ^3^ *J*_HH_ = 8.1 Hz, 1H, H6A), 6.40 (t, ^3^ *J*_HH_ = 6.5 Hz, 6.6 Hz, 1H, H1′A), 6.27 (td, ^3^ *J*_HH_ = 6.5 Hz, 6.5 Hz, ^5^ *J*_HF_ = 1.6 Hz, 1H, H1′B), 5.69 (d, ^3^ *J*_HH_ = 8.1 Hz, 1H, H5A), 5.40–5.36 (m, 1H, H3′A), 5.30 (brs, 1H, 5′A‐OH), 4.89 (d, ^3^ *J*_HH_ = 4.1 Hz, 1H, H4′B), 4.64–4.61 (m, 1H, H3′B), 4.26–4.23 (m, 1H, H4′A), 3.72–3.69 (m, 1H, H5′A), 3.63–3.60 (m, 1H, H5″A), 2.79–2.74 (m, 1H, H2′A), 2.69–2.63 (m, 1H, H2″A), 2.53–2.48 (m, 1H, H2′B), 2.29–2.24 (m, 1H, H2″B), 0.82 (s, 9H, CH_3_), 0.01 (s, 3H, CH_3_), −0.04 (s, 3H, CH_3_). ^13^C NMR *δ*: 163.1 (C4A), 157.3 (d, ^2^J_CF_ = 25 Hz, C4B), 150.4 (C2A), 149.2 (C2B), 145.3 (C4C), 140.7 (C6A), 140.2 (d, ^1^J_CF_ = 231 Hz, C5B), 124.8 (d, ^2^J_CF_ = 36 Hz, C6B), 123.6 (C5C), 101.9 (C5A), 84.72 (C1′B), 84.64 (C4′A), 84.34 (C1′A), 79.87 (C4′B), 75.58 (C3′B), 60.61 (C5′A), 59.33 (C3′A), 39.50 (C2′B), 37.40 (C2′A), 25.59 (CH_3_), 17.66 (C‐tBu), −4.97 (CH_3_Si). ^15^N NMR *δ*: −135.9 (N1B), −147.1 (N1A), −156.1 (N3A), −254.2 (N1C), −349.5 (N3C), −360.8 (N2C). ^19^F NMR *δ*: −166.6. ^29^Si NMR *δ*: 20.67. HRMS (ESI‐) calcd for C_25_H_33_FN_7_O_8_Si [M − H]^−^ 606.2143, found 606.2155.

**Dimer 6:** ^1^H NMR *δ*: 11.86 (brs, 1H, H3B), 11.37 (brs, 1H, H3A), 8.40 (s, 1H, H5C), 8.24 (d, ^3^ *J*_HF_ = 7.2 Hz, 1H, H6B), 7.97 (d, ^3^ *J*_HH_ = 8.1 Hz, 1H, H6A), 6.40 (t, ^3^ *J*_HH_ = 6.5 Hz, 6.6 Hz, 1H, H1′A), 6.30 (t, ^3^ *J*_HH_ = 5.9 Hz, 6.1 Hz, 1H, H1′B), 5.69 (d, ^3^ *J*_HH_ = 8.1 Hz, 1H, H5A), 5.63 (d, ^3^ *J*_HH_ = 4.2 Hz, 1H, 3′B‐OH), 5.39–5.35 (m, 1H, H3′A), 5.29 (t, ^3^ *J*_HH_ = 4.7 Hz, 4.9 Hz, 1H, 5′A‐OH), 4.93 (d, ^3^ *J*_HH_ = 3.2 Hz, 1H, H4′B), 4.46–4.43 (m, 1H, H3′B), 4.26 (m, 1H, H4′A), 3.72–3.68 (m, 1H, H5′A), 3.65–3.61 (m, 1H, H5″A), 2.80–2.75 (m, 1H, H2′A), 2.68–2.63 (m, 1H, H2″A), 2.47–2.42 (m, 1H, H2′B), 2.30–2.25 (m, 1H, H2″B). ^13^C NMR *δ*: 163.1 (C4A), 157.0 (d, ^2^ *J*_CF_ = 26 Hz, C4B), 150.4 (C2A), 149.1 (C2B), 145.8 (C4C), 140.7 (d, ^1^ *J*_CF_ = 231 Hz, C5B), 140.1 (C6A), 124.8 (d, ^2^ *J*_CF_ = 34 Hz, C6B), 123.4 (C5C), 101.9 (C5A), 84.97 (C1′B), 84.63 (C4′A), 84.37 (C1′A), 80.11 (C4′B), 74.07 (C3′B), 60.72 (C5′A), 59.40 (C3′A), 38.72 (C2′B), 37.38 (C2′A). ^15^N NMR *δ*: −135.6 (N1B), −146.9 (N1A), −158.1 (N3A), −254.1 (N1C), −349.6 (N3C), −360.6 (N2C). ^19^F NMR *δ*: −166.6. HRMS (ESI‐) calcd for C_19_H_19_FN_7_O_8_ [M − H]^−^ 492.1279, found 492.1280.

**Dimer 7:** ^1^H NMR *δ*: 11.35 (brs, 2H, H3A, H3B), 8.42 (s, 1H, H5C), 7.87 (d, ^3^ *J*_HH_ = 8.1 Hz, 1H, H6B), 7.81 (d, ^4^ *J*_HH_ = 1.2 Hz, 1H, H6A), 6.43 (t, ^3^ *J*_HH_ = 6.7 Hz, 6.7 Hz, 1H, H1′A), 6.29 (t, ^3^ *J*_HH_ = 6.6 Hz, 6.8 Hz, 1H, H1′B), 5.70 (d, ^3^ *J*_HH_ = 8.1 Hz, 1H, H5B), 5.41–5.37 (m, 1H, H3′A), 5.29 (t, ^3^ *J*_HH_ = 4.4 Hz, 4.5 Hz, 1H, 5′A‐OH), 4.90 (d, ^3^ *J*_HH_ = 3.2 Hz, 1H, H4′B), 4.65–4.62 (m, 1H, H3′B), 4.22–4.20 (m, 1H, H4′A), 3.73–3.69 (m, 1H, H5′A), 3.64–3.60 (m, 1H, H5″A), 2.75–2.63 (m, 2H, H2′A, H2″A), 2.50–2.46 (m, 1H, H2′B), 2.31–2.26 (m, 1H, H2″B), 1.81 (d, ^4^ *J*_HH_ = 1.2 Hz, 3H, ^5A^CH_3_), 0.83 (s, 9H, CH_3_), 0.02 (s, 3H, CH_3_), −0.03 (s, 3H, CH_3_). ^13^C NMR *δ*: 163.7 (C4A), 163.0 (C4B), 150.4 (C2B, C2A), 145.3 (C4C), 140.6 (C6B), 136.2 (C6A), 123.4 (C5C), 109.6 (C5A), 102.2 (C5B), 84.43 (C4′A), 84.33 (C1′B), 83.85 (C1′A), 79.91 (C4′B), 75.66 (C3′B), 60.69 (C5′A), 59.35 (C3′A), 39.51 (C2′B), 37.10 (C2′A), 25.59 (CH_3_), 17.67 (C‐tBu), 12.25 (^5A^CH_3_), −4.97 (CH_3_Si), −5.04 (CH_3_Si). ^15^N NMR *δ*: −144.0 (N1A), −146.5 (N1B), −156.1 (N3A), −158.2 (N3B), −254.2 (N1C), −350.6 (N3C), −360.8 (N2C). ^29^Si NMR *δ*: 20.64. HRMS (ESI+) calcd for C_26_H_38_N_7_O_8_Si [M + H]^+^ 604.2551, found 604.2548.

**Dimer 8:** ^1^H NMR *δ*: 11.34 (brs, 2H, H3A, H3B), 8.41 (s, 1H, H5C), 7.86 (d, ^3^ *J*_HH_ = 8.1 Hz, 1H, H6B), 7.83 (d, ^4^ *J*_HH_ = 1.2 Hz, 1H, H6A), 6.43 (t, ^3^ *J*_HH_ = 6.7 Hz, 6.7 Hz, 1H, H1′A), 6.32 (t, ^3^ *J*_HH_ = 6.5 Hz, 6.9 Hz, 1H, H1′B), 5.68 (d, ^3^ *J*_HH_ = 8.1 Hz, 1H, H5B), 5.66 (d, ^3^ *J*_HH_ = 4.3 Hz, 1H, 3′B‐OH), 5.41–5.37 (m, 1H, H3′A), 5.34 (t, ^3^ *J*_HH_ = 4.4 Hz, 4.6 Hz, 1H, 5′A‐OH), 4.93 (d, ^3^ *J*_HH_ = 3.1 Hz, 1H, H4′B), 4.48–4.45 (m, 1H, H3′B), 4.24–4.21 (m, 1H, H4′A), 3.73–3.69 (m, 1H, H5′A), 3.66–3.62 (m, 1H, H5′B), 2.76–2.71 (m, 1H, H2′A), 2.68–2.62 (m, 1H, H2″B), 2.44–2.38 (m, 1H, H2′B), 2.31–2.26 (m, 1H, H2″B), 1.81 (d, ^4^ *J*_HH_ = 1.1 Hz, 1H, ^5A^CH_3_). ^13^C NMR *δ*: 163.7 (C4A), 163.1 (C4B), 150.5 (C2B/C2A), 145.8 (C4C), 140.4 (C6B), 136.2 (C6A), 123.3 (C5C), 109.7 (C5A), 102.1 (C5B), 84.51 (C4′A), 84.47 (C1′B), 83.90 (C1′A), 80.09 (C4′B), 74.08 (C3′B), 60.79 (C5′A), 59.44 (C3′A), 38.89 (C2′B), 37.08 (C2′A), 12.25 (^5A^ CH_3_). ^15^N NMR *δ*: −144.1 (N1A), −146.1 (N1B), −158.1 (N3B), −254.3 (N1C), −350.2 (N3C), −360.7 (N2C). HRMS (ESI‐) calcd for C_20_H_22_N_7_O_8_ [M − H]^−^ 488.1529, found 488.1531.

**Dimer 9:** ^1^H NMR *δ*: 11.34 (brs, 2H, H3A, H3B), 8.42 (s, 1H, H5C), 7.81 (d, ^4^ *J*_HH_ = 1.2 Hz, 1H, H6A), 7.67 (d, ^4^ *J*_HH_ = 1.2 Hz, 1H, H6B), 6.42 (t, ^3^ *J*_HH_ = 6.7 Hz, 6.7 Hz, 1H, H1′A), 6.30 (t, ^3^ *J*_HH_ = 6.5 Hz, 6.5 Hz, 1H, H1′B), 5.41–5.37 (m, 1H, H3′A), 5.30 (t, ^3^ *J*_HH_ = 4.5 Hz, 4.6 Hz, 1H, 5′A‐OH), 4.89 (d, ^3^ *J*_HH_ = 3.2 Hz, 1H, H4′B), 4.69–4.67 (m, 1H, H3′B), 4.23–4.20 (m, 1H, H4′A), 3.73–3.69 (m, 1H, H5′A), 3.63–3.59 (m, 1H, H5″A), 2.76–2.71 (m, 1H, H2′A), 2.69–2.63 (m, 1H, H2″A), 2.53–2.48 (m, 1H, H2′B), 2.26–2.22 (m, 1H, H2″B), 1.81 (d, ^4^ *J*_HH_ = 1.2 Hz, 1H, ^5A^CH_3_), 1.77 (d, ^4^ *J*_HH_ = 1.2 Hz, 1H, ^5B^CH_3_), 0.83 (s, 9H, CH_3_), 0.03 (s, 3H, CH_3_), −0.01 (s, 3H, CH_3_). ^13^C NMR *δ*: 163.7 (C4A, C4B), 150.5 (C2B), 150.4 (C2A), 145.4 (C4C), 136.2 (C6A, C6B), 123.4 (C5C), 109.7 (C5A, C5B), 84.44 (C4′A), 84.22 (C1′A), 83.86 (C1′B), 79.99 (C4′B), 75.71 (C3′B), 60.65 (C5′A), 59.30 (C3′A), 39.51 (C2′B), 37.12 (C2′A), 25.61 (CH_3_), 17.69 (C‐tBu), 12.34 (^5A^CH_3_), 12.26 (^5B^CH_3_), −4.93 (CH_3_Si), −4.99 (CH_3_Si). ^15^N NMR *δ*: −143.2 (N1B), −144.1 (N1A), −254.2 (N1C), −350.7 (N3C), −360.8 (N2C). ^29^Si NMR *δ*: 20.50. HRMS (ESI+) calcd for C_27_H_40_N_7_O_8_Si [M + H]^+^ 618.2707, found 618.2702.

**Dimer 10:** ^1^H NMR *δ*: 11.33 (brs, 2H, H3A, H3B), 8.40 (s, 1H, H5C), 7.82 (d, ^4^ *J*_HH_ = 1.2 Hz, 1H, H6A), 7.66 (d, ^4^ *J*_HH_ = 1.2 Hz, 1H, H6B), 6.42 (t, ^3^ *J*_HH_ = 6.7 Hz, 6.7 Hz, 1H, H1′A), 6.33 (t, ^3^ *J*_HH_ = 6.5 Hz, 6.5 Hz, 1H, H1′B), 5.62 (d, ^3^ *J*_HH_ = 4.1 Hz, 1H, 3′B‐OH), 5.41–5.37 (m, 1H, H3′A), 5.29 (brs, 1H, 5′A‐OH), 4.91 (d, ^3^ *J*_HH_ = 3.1 Hz, 1H, H4′B), 4.54–4.51 (m, 1H, H3′B), 4.25–4.22 (m, 1H, H4′A), 3.73–3.69 (m, 1H, H5′A), 3.65–3.61 (m, 1H, H5″A), 2.77–2.72 (m, 1H, H2′A), 2.69–2.63 (m, 1H, H2″A), 2.46–2.41 (m, 1H, H2′B), 2.27–2.23 (m, 1H, H2″B), 1.81 (d, ^4^ *J*_HH_ = 1.1 Hz, 1H, ^5A^CH_3_), 1.75 (d, ^4^ *J*_HH_ = 1.1 Hz, 1H, ^5B^CH_3_). ^13^C NMR *δ*: 163.7 (C4A, C4B), 150.5 (C2B), 150.4 (C2A), 145.9 (C4C), 136.2 (C6A), 136.1 (C6B), 123.2 (C5C), 109.6 (C5A, C5B), 84.42 (C4′A), 84.27 (C1′B), 83.87 (C1′A), 80.05 (C4′B), 74.01 (C3′B), 60.74 (C5′A), 59.35 (C3′A), 38.69 (C2′B), 37.11 (C2′A), 12.32 (^5A^ CH_3_), 12.25 (^5B^ CH_3_). ^15^N NMR *δ*: −143.2 (N1B), −144.1 (N1A), −254.3 (N1C), −350.5 (N3C), −360.5 (N2C). HRMS (ESI‐) calcd for C_21_H_24_N_7_O_8_ [M − H]^−^ 502.1686, found 502.1679.

**Dimer 11:** ^1^H NMR *δ*: 11.83 (brs, 1H, H3B), 11.34 (brs, 1H, H3A), 8.45 (s, 1H, H5C), 8.26 (d, ^3^ *J*_HF_ = 7.2 Hz, 1H, H6B), 7.81 (d, ^4^ *J*_HH_ = 1.2 Hz, 1H, H6A), 6.42 (t, ^3^ *J*_HH_ = 6.6 Hz, 6.6 Hz, 1H, H1′A), 6.27 (td, ^3^ *J*_HH_ = 6.6 Hz, 6.5 Hz, ^5^ *J*_HF_ = 1.5 Hz, 1H, H1′B), 5.42–5.38 (m, 1H, H3′A), 5.29 (brs, 1H, 5′A‐OH), 4.90 (d, ^3^ *J*_HH_ = 4.0 Hz, 1H, H4′B), 4.65–4.61 (m, 1H, H3′B), 4.24–4.21 (m, 1H, H4′A), 3.73–3.69 (m, 1H, H'5A), 3.64–3.60 (m, 1H, H5″A), 2.77–2.63 (m, 2H, H2′A, H2″A), 2.55–2.50 (m, 1H, H2′B), 2.31–2.24 (m, 1H, H2″B), 1.81 (s, 3H, ^5A^CH_3_), 0.82 (s, 9H, CH_3_), 0.01 (s, 3H, CH_3_), −0.03 (s, 3H, CH_3_). ^13^C NMR *δ*: 163.7 (C4A), 157.0 (d, ^2^ *J*_CF_ = 25 Hz, C4B), 150.4 (C2A), 149.1 (C2B), 145.3 (C4C), 140.1 (d, ^1^ *J*_CF_ = 231 Hz, C5B), 136.2 (C6A), 124.9 (d, ^2^ *J*_CF_ = 36 Hz, C6B), 123.5 (C5C), 109.6 (C5A), 84.74 (C1′B), 84.43 (C4′A), 83.87 (C1′A), 79.91 (C4′B), 75.58 (C3′B), 60.63 (C5′A), 59.32 (C3′A), 38.75 (C2′B), 37.12 (C2′A), 25.59 (CH_3_), 17.65 (C‐tBu), 12.24 (^5B^ CH_3_), −4.98 (CH_3_Si), −5.05 (CH_3_Si). ^15^N NMR *δ*: −135.7 (N1B), −144.2 (N1A), −254.3 (N1C), −350.1 (N3C), −361.1 (N2C). ^19^F NMR *δ*: −166.6. ^29^Si NMR *δ*: 20.71. HRMS (ESI‐) calcd for C_26_H_35_FN_7_O_8_Si [M − H]^−^ 620.2300, found 620.2323.

**Dimer 12:** ^1^H NMR *δ*: 11.85 (brs, 1H, H3B), 11.35 (brs, 1H, H3A), 8.41 (s, 1H, H5C), 8.24 (d, ^3^ *J*_HF_ = 7.2 Hz, 1H, H6B), 7.82 (s, ^4^ *J*_HH_ = 1.2 Hz, 1H, H6A), 6.43 (t, ^3^ *J*_HH_ = 6.6 Hz, 6.6 Hz, 1H, H1′A), 6.30 (t, ^3^ *J*_HH_ = 6.6 Hz, 6.5 Hz, 1H, H1′B), 5.63 (brs, 1H, 3′B‐OH), 5.40–5.36 (m, 1H, H3′A), 5.29 (brs, 1H, 5′A‐OH), 4.93 (d, ^3^ *J*_HH_ = 4.0 Hz, 1H, H4′B), 4.45 (brs, 1H, H3′B), 4.24 (d, ^3^ *J*_HH_ = 2.1 Hz, 1H, H4′A), 3.73–3.70 (m, 1H, H5′A), 3.65–3.62 (m, 1H, H5″A), 2.78–2.62 (m, 2H, H2′A, H2″A), 2.46–2.41 (m, 1H, H2′B), 2.31–2.25 (m, 1H, H2″B), 1.81 (s, 3H, ^5A^CH_3_). ^13^C NMR *δ*: 163.7 (C4A), 157.0 (d, ^2^ *J*_CF_ = 25 Hz, C4B), 150.4 (C2A), 149.1 (C2B), 145.8 (C4C), 140.1 (d, ^1^ *J*_CF_ = 231 Hz, C5B), 136.2 (C6A), 124.8 (d, ^2^ *J*_CF_ = 36 Hz, C6B), 123.3 (C5C), 109.6 (C5A), 84.97 (C1′B), 84.41 (C4′A), 83.89 (C1′A), 80.12 (C4′B), 74.07 (C3′B), 60.73 (C5′A), 59.39 (C3′A), 38.73 (C2′B), 37.11 (C2′A), 12.25 (^5A^ CH_3_). ^15^N NMR *δ*: −135.9 (N1B), −144.2 (N1A), −254.3 (N1C), −349.0 (N3C), −360.3 (N2C). ^19^F NMR *δ*: −166.6. HRMS (ESI‐) calcd for C_20_H_21_FN_7_O_8_ [M − H]^−^ 506.1435, found 506.1444.

**Dimer 13:** ^1^H NMR *δ*: 11.33 (brs, 1H, H3B), 8.43 (s, 1H, H5C), 8.29 (d, ^3^ *J*_HF_ = 7.1 Hz, 1H, H6A), 7.88 (d, ^3^ *J*_HH_ = 8.1 Hz, 1H, H6B), 6.39 (td, ^3^ *J*_HH_ = 6.6 Hz, 6.6 Hz, ^5^ *J*_HF_ = 1.5 Hz, 1H, H1′A), 6.29 (t, ^3^ *J*_HH_ = 6.6 Hz, 6.5 Hz, 1H, H1′B), 5.69 (d, ^3^ *J*_HH_ = 8.2 Hz, 1H, H5B), 5.45 (brs, 1H, 5′A‐OH), 5.41–5.37 (m, 1H, H3′A), 4.89 (d, ^3^ *J*_HH_ = 4.1 Hz, 1H, H4′B), 4.65–4.62 (m, 1H, H3′B), 4.24–4.22 (m, 1H, H4′A), 3.74–3.71 (m, 1H, H5′A), 3.64–3.61 (m, 1H, H5″A), 2.78–2.73 (m, 1H, H2′A), 2.69–2.63 (m, 1H, H2″A), 2.50–2.45 (m, 1H, H2′B), 2.30–2.25 (m, 1H, H2″B), 0.82 (s, 9H, CH_3_), 0.01 (s, 3H, CH_3_), −0.03 (s, 3H, CH_3_). ^13^C NMR *δ*: 163.1 (C4B), 157.8 (d, ^2^ *J*_CF_ = 25 Hz, C4A), 150.5 (C2B), 149.6 (C2A), 145.2 (C4C), 140.6 (C6B), 140.2 (d, ^1^ *J*_CF_ = 231 Hz, C5A), 124.5 (d, ^2^ *J*_CF_ = 36 Hz, C6A), 123.5 (C5C), 102.2 (C5B), 84.73 (C4′A), 84.56 (C1′A), 84.32 (C1′B), 79.91 (C4′B), 75.68 (C3′B), 60.46 (C5′A), 59.08 (C3′A), 39.51 (C2′B), 37.40 (C2′A), 25.60 (CH_3_), 17.68 (C‐tBu), −4.97 (CH_3_Si), −5.04 (CH_3_Si). ^15^N NMR *δ*: −136.5 (N1A), −146.4 (N1B), −158.5 (N3B), −253.9 (N1C), −350.7 (N3C), −360.6 (N2C). ^19^F NMR *δ*: −166.8. ^29^Si NMR *δ*: 20.63. HRMS (ESI‐) calcd for C_25_H_33_FN_7_O_8_Si [M − H]^−^ 606.2143, found 606.2140.

**Dimer 14:** ^1^H NMR *δ*: 11.84 (brs, 1H, H3A), 11.35 (brs, 1H, H3B), 8.38 (s, 1H, H5C), 8.27 (d, 1H, ^3^ *J*_HF_ = 7.1 Hz, H6A), 7.86 (d, ^3^ *J*_HH_ = 8.1 Hz, 1H, H6B), 6.39 (td, ^3^ *J*_HH_ = 6.5 Hz, 6.6 Hz, ^5^ *J*_HF_ = 1.5 Hz, 1H, H1′A), 6.32 (t, ^3^ *J*_HH_ = 6.6 Hz, 6.5 Hz, 1H, H1′B), 5.68 (d, ^3^ *J*_HH_ = 8.1 Hz, 1H, H5B), 5.66 (brs, 1H, 3′B‐OH), 5.45 (brs, 1H, 5′B‐OH), 5.39–5.35 (m, 1H, H3′A), 4.92 (d, ^3^ *J*_HH_ = 4.1 Hz, 1H, H4′B), 4.47–4.45 (m, 1H, H3′B), 4.26–4.24 (m, 1H, H4′A), 3.74–3.71 (m, 1H, H5′A), 3.66–3.63 (m, 1H, H5″A), 2.78–2.73 (m, 1H, H2′A), 2.68–2.62 (m, 1H, H2″A), 2.43–2.38 (m, 1H, H2′B), 2.30–2.26 (m, 1H, H2″B). ^13^C NMR *δ*: 163.1 (C4B), 158.1 (d, ^2^ *J*_CF_ = 25 Hz, C4A), 150.5 (C2B), 149.8 (C2A), 145.8 (C4C), 140.4 (C6B), 140.3 (d, ^1^J_CF_ = 231 Hz, C5A), 124.4 (d, ^2^J_CF_ = 36 Hz, C6A), 123.3 (C5C), 102.1 (C5B), 84.69 (C4′A), 84.60 (C1′A), 84.52 (C1′B), 80.08 (C4′B), 74.08 (C3′B), 60.62 (C5′A), 59.18 (C3′A), 38.88 (C2′B), 37.38 (C2′A). ^15^N NMR *δ*: −136.3 (N1A), −146.3 (N1B), −157.8 (N3B), −253.8 (N1C), −350.4 (N3C), −360.5 (N2C). ^19^F NMR *δ*: −166.7. HRMS (ESI‐) calcd for C_19_H_19_FN_7_O_8_ [M − H]^−^ 492.1279, found 492.1281.

**Dimer 15:** ^1^H NMR *δ*: 11.88 (brs, 1H, H3A), 11.34 (brs, 1H, H3B), 8.41 (s, 1H, H5C), 8.34 (d, 1H, ^3^ *J*_HF_ = 7.1 Hz, H6A), 7.67 (d, ^4^ *J*_HH_ = 1.2 Hz, 1H, H6B), 6.38 (td, ^3^ *J*_HH_ = 6.6 Hz, 6.6 Hz, ^5^ *J*_HF_ = 1.6 Hz, 1H, H1′A), 6.30 (t, ^3^ *J*_HH_ = 6.6 Hz, 6.5 Hz, 1H, H1′B), 5.43 (t, ^3^ *J*_HH_ = 4.4 Hz, 4.6 Hz, 1H, 5′A‐OH), 5.41–5.37 (m, 1H, H3′A), 4.89 (d, ^3^ *J*_HH_ = 3.2 Hz, 1H, H4′B), 4.70–4.67 (m, 1H, H3′B), 4.27–4.24 (m, 1H, H4′A), 3.76–3.72 (m, 1H, H5′A), 3.64–3.60 (m, 1H, H5″A), 2.81–2.76 (m, 1H, H2′A), 2.71–2.65 (m, 1H, H2″A), 2.53–2.48 (m, 1H, H2′B), 2.26–2.22 (m, 1H, H2″B), 1.77 (d, ^4^ *J*_HH_ = 1.2 Hz, 1H, ^5B^CH_3_), 0.83 (s, 9H, CH_3_), 0.03 (s, 3H, CH_3_), −0.01 (s, 3H, CH_3_). ^13^C NMR *δ*: 163.7 (C4B), 157.0 (d, ^2^J_CF_ = 26 Hz, C4A), 150.5 (C2B), 149.0 (C2A), 145.4 (C4C), 140.0 (d, ^1^ *J*_CF_ = 230 Hz, C5A), 136.2 (C6B), 124.9 (d, ^2^ *J*_CF_ = 34 Hz, C6A), 123.4 (C5C), 109.6 (C5B), 84.74 (C4′A), 84.53 (C1′A), 84.20 (C1′B), 79.96 (C4′B), 75.69 (C3′B), 60.36 (C5′A), 58.91 (C3′A), 39.51 (C2′B), 37.40 (C2′B), 25.60 (CH_3_), 17.68 (C‐tBu), 12.32 (^5B^ CH_3_), −5.01 (CH_3_Si). ^15^N NMR *δ*: −136.5 (N1A), −143.4 (N1B), −253.8 (N1C), −350.8 (N3C), −360.8 (N2C). ^19^F NMR *δ*: −167.1. ^29^Si NMR *δ*: 20.50. HRMS (ESI‐) calcd for C_26_H_35_FN_7_O_8_Si [M − H]^−^ 620.2300, found 620.2310.

**Dimer 16:** ^1^H NMR *δ*: 11.87 (brs, 1H, H3A), 11.32 (brs, 1H, H3B), 8.38 (s, 1H, H5C), 8.34 (d, ^3^ *J*_HF_ = 7.1 Hz, 1H, H6A), 7.65 (d, ^4^ *J*_HH_ = 1.2 Hz, 1H, H6B), 6.38 (td, ^3^ *J*_HH_ = 6.6 Hz, 6.6 Hz, ^5^ *J*_HF_ = 1.6 Hz, 1H, H1′A), 6.33 (t, ^3^ *J*_HH_ = 6.6 Hz, 6.5 Hz, 1H, H1′B), 5.61 (d, ^3^ *J*_HH_ = 4.1 Hz, 1H, 3′B‐OH), 5.42 (t, ^3^ *J*_HH_ = 4.4 Hz, 4.6 Hz, 1H, 5′A‐OH), 5.40–5.36 (m, 1H, H3′A), 4.91 (d, ^3^ *J*_HH_ = 3.2 Hz, 1H, H4′B), 4.54–4.50 (m, 1H, H3′B), 4.28–4.26 (m, 1H, H4′A), 3.76–3.72 (m, 1H, H5′A), 3.66–3.62 (m, 1H, H5″A), 2.82–2.76 (m, 1H, H2′A), 2.70–2.65 (m, 1H, H2″A), 2.46–2.40 (m, 1H, H2′B), 2.27–2.23 (m, 1H, H2″B), 1.75 (s, 3H, ^5B^CH_3_). ^13^C NMR *δ*: 163.6 (C4B), 157.8 (d, ^2^ *J*_CF_ = 26 Hz, C4A), 150.5 (C2B), 149.0 (C2A), 145.9 (C4C), 140.0 (d, ^1^ *J*_CF_ = 230 Hz, C5A), 136.1 (C6B), 124.9 (d, ^2^ *J*_CF_ = 34 Hz, C6A), 123.3 (C5C), 109.6 (C5B), 84.74 (C4′A), 84.56 (C1′A), 84.26 (C1′B), 80.04 (C4′B), 74.01 (C3′B), 60.47 (C5′A), 58.98 (C3′A), 38.66 (C2′B), 37.39 (C2′A), 12.31 (^5B^ CH_3_). ^15^N NMR *δ*: −136.6 (N1A), −143.5 (N1B), −156.1 (N3B), −253.7 (N1C), −350.7 (N3C), −360.5 (N2C). ^19^F NMR *δ*: −167.1. HRMS (ESI‐) calcd for C_20_H_21_FN_7_O_8_ [M − H]^−^ 506.1435, found 506.1442.

**Dimer 17:** ^1^H NMR *δ*: 11.88 (brs, 2H, H3A, H3B), 8.44 (s, 1H, H5C), 8.34 (d, ^3^ *J*_HF_ = 7.1 Hz, 1H, H6B), 8.26 (d, ^3^ *J*_HF_ = 7.1 Hz, 1H, H6A), 6.37 (td, ^3^ *J*_HH_ = 6.5 Hz, 6.6 Hz, ^5^ *J*_HF_ = 1.5 Hz, 1H, H1′A), 6.27 (td, ^3^ *J*_HH_ = 6.5 Hz, 6.5 Hz, ^5^ *J*_HF_ = 1.5 Hz, 1H, H1′B), 5.43–5.37 (m, 2H, H3′A, 5′A‐OH), 4.89 (d, ^3^ *J*_HH_ = 3.2 Hz, 1H, H4′B), 4.64–4.61 (m, 1H, H3′B), 4.28–4.25 (m, 1H, H4′A), 3.77–3.72 (m, 1H, H5′A), 3.65–3.60 (m, 1H, H5″A), 2.81–2.75 (m, 1H, H2′A), 2.72–2.65 (m, 1H, H2″A), 2.55–2.48 (m, 1H, H2′B), 2.30–2.24 (m, 1H, H2″B), 0.82 (s, 9H, CH_3_), 0.01 (s, 3H, CH_3_), −0.04 (s, 3H, CH_3_). ^13^C NMR *δ*: 157.1 (d, ^2^ *J*_CF_ = 26 Hz, C4A), 156.9 (d, ^2^ *J*_CF_ = 26 Hz, C4B), 149.0 (C2B, C2A), 145.2 (C4C), 140.1 (d, ^1^ *J*_CF_ = 230 Hz, C5B), 140.0 (d, ^1^ *J*_CF_ = 230 Hz, C5A), 125.1 (d, ^2^ *J*_CF_ = 34 Hz, C6A), 124.8 (d, ^2^ *J*_CF_ = 34 Hz, C6B), 123.6 (C5C), 84.72 (C1′B, C4′A), 84.54 (C1′A), 79.88 (C4′B), 75.54 (C3′B), 60.34 (C5′A), 58.91 (C3′A), 38.88 (C2′B), 37.40 (C2′A), 25.57 (CH_3_), 17.63 (C‐tBu), −5.00 (CH_3_Si), −5.08 (CH_3_Si). ^15^N NMR *δ*: −135.5 (N1B), −136.3 (N1A), −158.1 (N3A, N3B), −253.5 (N1C), −349.7 (N3C), −360.5 (N2C). ^19^F NMR *δ*: −166.7, 167.1. ^29^Si NMR *δ*: 20.69. HRMS (ESI‐) calcd for C_25_H_32_F_2_N_7_O_8_Si [M − H]^−^ 624.2049, found 624.2072. HRMS (ESI+) calcd for C_25_H_34_F_2_N_7_O_8_Si [M + H]^+^ 626.2206.

**Dimer 18:** ^1^H NMR *δ*: 11.88 (brs, 2H, H3A, H3B), 8.39 (s, 1H, H5C), 8.34 (d, ^3^ *J*_HF_ = 7.1 Hz, 1H, H6B), 8.23 (d, ^3^ *J*_HF_ = 7.1 Hz, 1H, H6A), 6.38 (td, ^3^ *J*_HH_ = 6.5 Hz, 6.6 Hz, ^5^ *J*_HF_ = 1.5 Hz, 1H, H1′A), 6.30 (td, ^3^ *J*_HH_ = 6.5 Hz, 6.5 Hz, ^5^ *J*_HF_ = 1.5 Hz, 1H, H1′B), 5.62 (d, 1H, ^3^ *J*_HH_ = 4.2 Hz, 3′B‐OH), 5.43 (t, ^3^ *J*_HH_ = 4.5 Hz, 4.6 Hz, 1H, 5′A‐OH), 5.40–5.36 (m, 1H, H3′A), 4.93 (d, ^3^ *J*_HH_ = 3.2 Hz, 1H, H4′B), 4.46–4.43 (m, 1H, H3′B), 4.29–4.26 (m, 1H, H4′A), 3.76–3.72 (m, 1H, H5′A), 3.66–3.62 (m, 1H, H5″A), 2.81–2.76 (m, 1H, H2′A), 2.70–2.65 (m, 1H, H2′B), 2.47–2.42 (m, 1H, H2′B), 2.30–2.25 (m, 1H, H2″B). ^13^C NMR *δ*: 157.2 (d, ^2^ *J*_CF_ = 26 Hz, C4A, C4B), 149.2 (C2A, C2B), 145.9 (C4C), 140.2 (d, ^1^ *J*_CF_ = 230 Hz, C5A, C5B), 125.0 (d, ^2^ *J*_CF_ = 35 Hz, C6A), 124.9 (d, ^2^ *J*_CF_ = 35 Hz, C6B), 123.5 (C5C), 85.12 (C1′B), 84.88 (C4′A), 84.74 (C1′A), 80.19 (C4′B), 74.19 (C3′B), 60.61 (C5′A), 59.19 (C3′A), 38.83 (C2′B), 37.52 (C2′A). ^15^N NMR *δ*: −135.6 (N1A), −136.4 (N1B), −253.7 (N1C), −349.1 (N3C), −360.1 (N2C). ^19^F NMR *δ*: −166.6, −166.7. HRMS (ESI‐) calcd for C_19_H_18_F_2_N_7_O_8_ [M − H]^−^ 510.1184, found 510.1190.

### 4.2 NMR, MS, and HPLC Data

1D and 2D ^1^H, ^13^C, ^15^N, ^19^F, and ^29^Si NMR spectra were recorded on a Bruker Avance III 500 MHz spectrometer, equipped with 5 mm broad‐band multinuclear (PABBO) probe in DMSO‐d_6_ at 298 K. Chemical shifts (*δ*) for ^1^H, ^13^C, and ^29^Si NMR were reported in ppm relative to the tetramethylsilane (TMS) peak. For ^31^F and ^15^N NMR *δ* were reported in ppm, relative to trichlorofluoromethane and liquid NH_3_, respectively. ^1^H, ^13^C, and ^15^N signals were assigned by means of ^1^H–^1^H COSY, ^1^H–^13^C/^15^N HSQC, and ^1^H–^13^C/^15^N HMBC experiments. The 2‐D homo‐ or heteronuclear correlation experiments were carried out using a standard set of parameters. The spectra were processed, analyzed, and prepared with TopSpin 4.5 Bruker Software.

MS analysis of samples was performed on an Exploris 480 quadrupole‐Orbitrap mass spectrometer (Thermo Scientific, Bremen, Germany) equipped with an HESI ion source. The source was operated at a gas pressure of 1.25 psi, and the ionization voltage was set to 4 kV. External calibration was used using a recommended Thermo Calibration Mixture (FlexMix). MS data were acquired in positive ion mode within the range of m/z 150–1500 at the resolution of 120,000 (at m/z 200, FWHM). Automatic gain control was set to target value of 1 × 10^6^ and ion injection time (IT) was 100 ms. MS/MS acquisition was carried out in data‐dependent mode at the resolution of 30,000 (at m/z 200, FWHM). Raw data were analyzed in Thermo FreeStyle 1.8 software.

HPLC analysis was performed using 0.02 mM concentration for all samples on the column LiChrosorb RP‐18 (5.0 mm, 4.6 mm × 250 mm), using two buffers (A + B): buffer A: 0.01 M aqueous triethylammonium acetate, pH 7.4; buffer B: A/acetonitrile (1:4, v/v), at 35°C, flow rate 1.5 mL/min; events: 5 min A 100%, linear gradient of B 0%–100% in 20 min, 5 min B 100% and A 100% 15 min wash.

### 4.3 Biology

#### 4.3.1 In Silico Pharmacokinetic Prediction

Calculations of pharmacokinetic profile descriptors of synthesized compounds were performed by the SwissADME server (Daina et al. 2017).

#### 4.3.2 Cell Line and Culture Conditions

GBM cell lines (U‐251 MG, U‐138 MG), SK‐OV‐3 (ovarian cancer cell line), HeLa (cervical cancer cell line), T47D (breast cancer cell line), and non‐cancerous lung fibroblast cell line (MRC‐5) were purchased from ATCC (Manassas, USA). All cell lines were from human origin. HeLa and T47D were cultured in RPMI 1640 medium. U‐251 MG, U‐138 MG, and MRC‐5 were cultured in EMEM medium. SK‐OV‐3 was cultured in McCoy's 5A medium. Each medium was supplemented with 10% fetal bovine serum (FBS) and 10 mg/mL antibiotics (penicillin and streptomycin). Cells were cultured at 37°C with 5% CO_2_ in humidified air. Cell media (RPMI 1640, DMEM, EMEM, McCoy's 5A), Human Blood Plasma (HBP) were obtained from Sigma–Aldrich Chemie GmbH (Steinheim, Germany) and ATCC. Cell concentrations in culture were adjusted to allow for exponential growth.

#### 4.3.3 Morphology Analysis

After treatment with nucleoside dimers, U‐251 MG cells were imaged using a Keyence BZ‐X810 fluorescence microscope at a magnification of 10× (Keyence, Neu‐Isenburg, Germany).

#### 4.3.4 Cytotoxicity Assay (IC_50_ , μM)

Cell viability was evaluated by a dye staining method using 3‐(4,5‐dimethyl‐2‐thiazolyl)‐2,5‐diphenyl‐2H‐tetrazolium bromide (MTT). The protocol was adapted from the literature methods (Xavier et al. 2011). The monolayer cell culture was trypsinized and counted. To each well of the 96‐well plate, 100 μL of the diluted cell suspension (1 × 10^4^ cells) was added. After 24 h, when a partial monolayer was formed, 100 μL of fresh medium with different compound concentrations (15.625, 31.25, 62.5, 125, 250, 500, 1000 and 2000 μg/mL) was added to the cells. After 72 h, the supernatant was washed out and 100 μL of MTT solution in medium (final concentration 0.5 mg/mL) was added to each well for 2 h. After the incubation time was complete, unreacted dye was removed by aspiration. The formazan crystals were dissolved in 100 μL/well DMSO and measured spectrophotometrically in a multi‐well Synergy2 plate reader (BioTek Instruments, USA) at a test wavelength of 492 nm and a reference wavelength of 690 nm. The results were calculated as an IC_50_ (inhibitory concentration 50) – the IC_50_ corresponds to the concentration of tested compound that inhibits cell viability/proliferation by 50%. Results are presented as mean of at least three independent experiments.

#### 4.3.5 In Vitro Cell Viability (Live/Dead Staining)

For the visualization of live and dead U‐251 MG cells upon incubation with nucleoside dimers and controls, a Live/Dead staining was performed. Cells were seeded in black polystyrene 96‐wells flat bottom plate with the transparent bottom (Greiner Bio‐One GmbH) at densities of 1 × 10^4^ cells/well. After 24 h, medium containing an increasing concentration of nucleoside dimers was added to each well. Following 24 and 48 h exposure to the nanomaterials, cells were incubated with 2 μM calcein AM (L3224, ThermoFisher Scientific), 2 μM ethidium homodimer‐1 (L3224, ThermoFisher Scientific), and 8 μM Hoechst 33342 (14533, Sigma‐Aldrich) containing PBS (100 μL/well) during 30 min at 37°C. Finally, the cells were analyzed with the Keyence BZ‐X810 fluorescence microscope at 10× magnification (Keyence, Neu‐Isenburg, Germany). Viable cells (green signal) were imaged using the GFP excitation/emission filter (475/525 nm), while for the dead cells (red signal), the TRITC ex/em filter (545/605 nm) combination was applied. DAPI ex/em filter (360/460 nm) was applied to detect the Hoechst 33342 blue signal (cell nuclei).

#### 4.3.6 Measurement of ROS

Reactive oxygen species (ROS) in live U‐251 MG cells were quantified using a DCF‐DA (C2938, ThermoFischer Scientific). 2′,7′‐dichlorofluorescin diacetate (DCF‐DA) is a cell‐permeant reagent that undergoes deacetylation and transforms into a non‐fluorescent substance in living cells. ROS oxidize it into 2′,7′‐dichlorofluorescein (DCF), an intensely fluorescent compound.

Cells were seeded 96‐well plate (1 × 104 cells/well) and allowed to attach overnight. After a single wash with HBSS buffer, the cells were stained for 30 min at 37°C with 25 μM DCF‐DA and then washed once more with HBSS buffer. In the next step, nucleoside dimers in HBSS buffer were added to the cells for 30 min at 37°C and then washed once more with HBSS buffer. Fluorescence was assessed using a Hidex Sense multimode reader (Hidex Oy, Turku, Finland) with excitation and emission wavelengths set to 485 and 530 nm, respectively. All measurements were performed in 30‐min steps.

#### 4.3.7 Migration Analysis Using the IncuCyte System

Cell migration was evaluated using the live‐cell imaging platform IncuCyte Live‐Cell Analysis System, which enables real‐time, non‐invasive monitoring of cellular behavior under physiological conditions. The assay was performed using a wound healing (scratch) approach. Briefly, cells were seeded in 96‐well ImageLock plates and cultured until a confluent monolayer was formed. A uniform scratch (“wound”) was then generated in each well using a dedicated wound‐making tool. After removing detached cells by gentle washing with culture medium, fresh medium containing the tested nucleoside dimers was added. Plates were immediately transferred to the IncuCyte system, where images of each well were automatically acquired at regular intervals (every 6 h) over the course of the experiment. The system's integrated software was used to quantify cell migration by measuring the relative wound density or percentage of wound closure over time. Migration was expressed as the rate of wound closure compared to control conditions. This method allowed continuous monitoring of treatment effects on cell motility, providing quantitative and kinetic data on how nucleoside dimers influence cellular migration.

#### 4.3.8 Cell Cycle Analysis

Cell cycle distribution was analyzed by flow cytometry following treatment with nucleoside dimers. U‐251 MG cells were exposed to nucleoside dimers at concentrations of 5, 10, and 50 μM for 48 h. After treatment, cells were harvested, treated with RNase A, and stained with propidium iodide (PI) according to the manufacturer's protocol. DNA content was analyzed using an Agilent NovoCyte 3000 flow cytometer (Agilent Technologies), and the distribution of cells in the G0/G1, S, and G2/M phases of the cell cycle was determined based on DNA content.

#### 4.3.9 Clonogenic Assay

The clonogenic assay was performed to evaluate the long‐term proliferative capacity of U‐251 MG cells following treatment with nucleoside dimers. Cells were seeded into 6‐well plates at a density of 500 cells per well and allowed to attach for 24 h. Subsequently, the cells were treated with nucleoside dimers at concentrations of 5, 10, and 50 μM. After treatment, the cells were cultured for an additional 7 days to allow colony formation. At the end of the incubation period, colonies were fixed and stained with crystal violet, and colonies consisting of at least 50 cells were counted manually. The colony formation efficiency was expressed relative to the untreated control.

## Author Contributions

**Dagmara Baraniak:** conceptualization, investigation, funding acquisition, writing – original draft, methodology, writing – review and editing, formal analysis, project administration, data curation, supervision. **Dariusz Wawrzyniak:** investigation, writing – original draft, methodology, formal analysis. **Daniel Baranowski:** investigation, writing – original draft, methodology, formal analysis.

## Funding

This work was supported by the National Science Centre, Poland (2017/01/X/ST5/00381, 2019/35/D/NZ7/03637).

## Conflicts of Interest

The authors declare no conflicts of interest.

## Supporting information


**Figure S1:** HPLC chromatogram.
**Figure S2:** ^1^H NMR spectrum in DMSO‐d_6_.
**Figure S3:** ^13^C NMR spectrum in DMSO‐d_6_.
**Figure S4:** HPLC chromatogram.
**Figure S5:** HPLC chromatogram.
**Figure S6:** HPLC chromatogram.
**Figure S7:** (−) MS‐ESI spectrum.
**Figure S8:** ^1^H NMR spectrum in DMSO‐d_6_.
**Figure S9:** ^13^C NMR spectrum in DMSO‐d_6_.
**Figure S10:** ^1^H‐^1^H COSY spectrum in DMSO‐d_6_.
**Figure S11:** ^1^H‐^13^C gHSQC spectrum in DMSO‐d_6_.
**Figure S12:** ^1^H‐^13^C gHMBC spectrum in DMSO‐d_6_.
**Figure S13:** ^1^H‐^15^N gHSQC spectrum in DMSO‐d_6_.
**Figure S14:** ^1^H‐^15^N gHMBC spectrum in DMSO‐d_6_.
**Figure S15:** HPLC chromatogram.
**Figure S16:** (−) MS‐ESI spectrum.
**Figure S17:** (+) MS‐ESI spectrum.
**Figure S18:** ^1^H NMR spectrum in DMSO‐d_6_.
**Figure S19:** ^13^C NMR spectrum in DMSO‐d_6_.
**Figure S20:** ^1^H‐^1^H COSY spectrum in DMSO‐d_6_.
**Figure S21:** ^1^H‐^13^C gHSQC spectrum in DMSO‐d_6_.
**Figure S22:** ^1^H‐^13^C gHMBC spectrum in DMSO‐d_6_.
**Figure S23:** ^1^H‐^15^N gHMBC spectrum in DMSO‐d_6_.
**Figure S24:** HPLC chromatogram.
**Figure S25:** (−) MS‐ESI spectrum.
**Figure S26:** (+) MS‐ESI spectrum.
**Figure S27:** ^1^H NMR spectrum in DMSO‐d_6_.
**Figure S28:** ^13^C NMR spectrum in DMSO‐d_6_.
**Figure S29:** ^19^F NMR spectrum in DMSO‐d_6_.
**Figure S30:** HPLC chromatogram.
**Figure S31:** (−) MS‐ESI spectrum.
**Figure S32:** (+) MS‐ESI spectrum.
**Figure S33:** ^1^H NMR spectrum in DMSO‐d_6_.
**Figure S34:** ^13^C NMR spectrum in DMSO‐d_6_.
**Figure S35:** ^1^H‐^1^H COSY spectrum in DMSO‐d_6_.
**Figure S36:** ^1^H‐^13^C gHSQC spectrum in DMSO‐d_6_.
**Figure S37:** ^1^H‐^13^C gHMBC spectrum in DMSO‐d_6_.
**Figure S38:** ^1^H‐^15^N gHMBC spectrum in DMSO‐d_6_.
**Figure S39:** ^29^Si NMR spectrum in DMSO‐d_6_.
**Figure S40:** HPLC chromatogram.
**Figure S41:** (−) MS‐ESI spectrum.
**Figure S42:** (+) MS‐ESI spectrum.
**Figure S43:** ^1^H NMR spectrum in DMSO‐d_6_.
**Figure S44:** ^13^C NMR spectrum in DMSO‐d_6_.
**Figure S45:** ^1^H‐^1^H COSY spectrum in DMSO‐d_6_.
**Figure S46:** ^1^H‐^13^C gHSQC spectrum in DMSO‐d_6_.
**Figure S47:** ^1^H‐^13^C gHMBC spectrum in DMSO‐d_6_.
**Figure S48:** ^1^H‐^15^N gHMBC spectrum in DMSO‐d_6_.
**Figure S49:** ^29^Si NMR spectrum in DMSO‐d_6_.
**Figure S50:** HPLC chromatogram.
**Figure S51:** (−) MS‐ESI spectrum.
**Figure S52:** (+) MS‐ESI spectrum.
**Figure S53:** ^1^H NMR spectrum in DMSO‐d_6_.
**Figure S54:** ^13^C NMR spectrum in DMSO‐d_6_.
**Figure S55:** ^1^H‐^1^H COSY spectrum in DMSO‐d_6_.
**Figure S56:** ^1^H‐^13^C gHSQC spectrum in DMSO‐d_6_.
**Figure S57:** ^1^H‐^13^C gHMBC spectrum in DMSO‐d_6_.
**Figure S58:** ^1^H‐^15^N gHSQC spectrum in DMSO‐d_6_.
**Figure S59:** ^1^H‐^15^N gHMBC spectrum in DMSO‐d_6_.
**Figure S60:** ^29^Si NMR spectrum in DMSO‐d.
**Figure S61:** ^19^F NMR spectrum in DMSO‐d_6_.
**Figure S62:** HPLC chromatogram.
**Figure S63:** (−) MS‐ESI spectrum.
**Figure S64:** (+) MS‐ESI spectrum.
**Figure S65:** ^1^H NMR spectrum in DMSO‐d_6_.
**Figure S66:** ^13^C NMR spectrum in DMSO‐d_6_.
**Figure S67:** ^1^H‐^1^H COSY spectrum in DMSO‐d_6_.
**Figure S68:** ^1^H‐^13^C gHSQC spectrum in DMSO‐d_6_.
**Figure S69:** ^1^H‐^13^C gHMBC spectrum in DMSO‐d_6_.
**Figure S70:** ^1^H‐^15^N gHSQC spectrum in DMSO‐d_6_.
**Figure S71:** ^1^H‐^15^N gHMBC spectrum in DMSO‐d_6_.
**Figure S72:** ^29^Si NMR spectrum in DMSO‐d_6_.
**Figure S73:** HPLC chromatogram.
**Figure S74:** (−) MS‐ESI spectrum.
**Figure S75:** ^1^H NMR spectrum in DMSO‐d_6_.
**Figure S76:** ^13^C NMR spectrum in DMSO‐d_6_.
**Figure S77:** ^1^H‐^1^H COSY spectrum in DMSO‐d_6_.
**Figure S78:** ^1^H‐^13^C gHSQC spectrum in DMSO‐d_6_.
**Figure S79:** ^1^H‐^13^C gHMBC spectrum in DMSO‐d_6_.
**Figure S80:** ^1^H‐^15^N gHMBC spectrum in DMSO‐d_6_.
**Figure S81:** HPLC chromatogram.
**Figure S82:** (−) MS‐ESI spectrum.
**Figure S83:** ^1^H NMR spectrum in DMSO‐d_6_.
**Figure S84:** ^13^C NMR spectrum in DMSO‐d_6_.
**Figure S85:** ^1^H‐^1^H COSY spectrum in DMSO‐d_6_.
**Figure S86:** ^1^H‐^13^C gHSQC spectrum in DMSO‐d_6_.
**Figure S87:** ^1^H‐^13^C gHMBC spectrum in DMSO‐d_6_.
**Figure S88:** ^1^H‐^15^N gHSQC spectrum in DMSO‐d_6_.
**Figure S89:** ^1^H‐^15^N gHMBC spectrum in DMSO‐d_6_.
**Figure S90:** ^29^Si NMR spectrum in DMSO‐d_6_.
**Figure S91:** HPLC chromatogram.
**Figure S92:** (−) MS‐ESI spectrum.
**Figure S93:** ^1^H NMR spectrum in DMSO‐d_6_.
**Figure S94:** ^13^C NMR spectrum in DMSO‐d_6_.
**Figure S95:** ^1^H‐^1^H COSY spectrum in DMSO‐d_6_.
**Figure S96:** ^1^H‐^13^C gHSQC in DMSO‐d_6_.
**Figure S97:** ^1^H‐^13^C gHMBC spectrum in DMSO‐d_6_.
**Figure S98:** ^1^H‐^15^N gHMBC spectrum in DMSO‐d_6_.
**Figure S99:** HPLC chromatogram.
**Figure S100:** (−) MS‐ESI spectrum.
**Figure S101:** ^1^H NMR spectrum in DMSO‐d_6_.
**Figure S102:** ^13^C NMR spectrum in DMSO‐d_6_.
**Figure S103:** ^1^H‐^1^H COSY spectrum in DMSO‐d_6_.
**Figure S104:** ^1^H‐^13^C gHSQC spectrum in DMSO‐d_6_.
**Figure S105:** ^1^H‐^13^C gHMBC spectrum in DMSO‐d_6_.
**Figure S106:** ^1^H‐^15^N gHMBC spectrum in DMSO‐d_6_.
**Figure S107:** ^19^F NMR spectrum in DMSO‐d_6_.
**Figure S108:** ^29^Si NMR spectrum in DMSO‐d_6_.
**Figure S109:** HPLC chromatogram.
**Figure S110:** (−) MS‐ESI spectrum.
**Figure S111:** ^1^H NMR spectrum in DMSO‐d_6_.
**Figure S112:** ^13^C NMR spectrum in DMSO‐d_6_.
**Figure S113:** ^1^H‐^1^H COSY spectrum in DMSO‐d_6_.
**Figure S114:** ^1^H‐^13^C gHSQC spectrum in DMSO‐d_6_.
**Figure S115:** ^1^H‐^13^C gHMBC spectrum in DMSO‐d_6_.
**Figure S116:** ^1^H‐^15^N gHSQC spectrum in DMSO‐d_6_.
**Figure S117:** ^1^H‐^15^N gHMBC spectrum in DMSO‐d_6_.
**Figure S118:** ^19^F NMR spectrum in DMSO‐d_6_.
**Figure S119:** HPLC chromatogram.
**Figure S120:** (−) MS‐ESI spectrum.
**Figure S121:** (+) MS‐ESI spectrum.
**Figure S122:** ^1^H NMR spectrum in DMSO‐d_6_.
**Figure S123:** ^13^C NMR spectrum in DMSO‐d_6_.
**Figure S124:** ^1^H‐^1^H COSY spectrum in DMSO‐d_6_.
**Figure S125:** ^1^H‐^13^C gHSQC in DMSO‐d_6_.
**Figure S126:** ^1^H‐^13^C gHMBC spectrum in DMSO‐d_6_.
**Figure S127:** ^1^H‐^15^N gHSQC spectrum in DMSO‐d_6_.
**Figure S128:** ^1^H‐^15^N gHMBC spectrum in DMSO‐d_6_.
**Figure S129:** ^29^Si NMR spectrum in DMSO‐d_6_.
**Figure S130:** HPLC chromatogram.
**Figure S131:** (−) MS‐ESI spectrum.
**Figure S132:** ^1^H NMR spectrum in DMSO‐d_6_.
**Figure S133:** ^13^C NMR spectrum in DMSO‐d_6_.
**Figure S134:** ^1^H‐^1^H COSY spectrum in DMSO‐d_6_.
**Figure S135:** ^1^H‐^13^C gHSQC in DMSO‐d_6_.
**Figure S136:** ^1^H‐^13^C gHMBC spectrum in DMSO‐d_6_.
**Figure S137:** ^1^H‐^15^N gHMBC spectrum in DMSO‐d_6_.
**Figure S138:** HPLC chromatogram.
**Figure S139:** (−) MS‐ESI spectrum.
**Figure S140:** (+) MS‐ESI spectrum.
**Figure S141:** ^1^H NMR spectrum in DMSO‐d_6_.
**Figure S142:** ^13^C NMR spectrum in DMSO‐d_6_.
**Figure S143:** ^1^H‐^1^H COSY spectrum in DMSO‐d_6_.
**Figure S144:** ^1^H‐^13^C gHSQC spectrum in DMSO‐d_6_.
**Figure S145:** ^1^H‐^13^C gHMBC spectrum in DMSO‐d_6_.
**Figure S146:** ^1^H‐^15^N gHMBC spectrum in DMSO‐d_6_.
**Figure S147:** ^29^Si NMR spectrum in DMSO‐d_6_.
**Figure S148:** HPLC chromatogram.
**Figure S149:** (−) MS‐ESI spectrum.
**Figure S150:** ^1^H NMR spectrum in DMSO‐d_6_.
**Figure S151:** ^13^C NMR spectrum in DMSO‐d_6_.
**Figure S152:** ^1^H‐^1^H COSY spectrum in DMSO‐d_6_.
**Figure S153:** ^1^H‐^13^C gHSQC spectrum in DMSO‐d_6_.
**Figure S154:** ^1^H‐^13^C gHMBC spectrum in DMSO‐d_6_.
**Figure S155:** ^1^H‐^15^N gHMBC spectrum in DMSO‐d_6_.
**Figure S156:** HPLC chromatogram.
**Figure S157:** (−) MS‐ESI spectrum.
**Figure S158:** (+) MS‐ESI spectrum.
**Figure S159:** ^1^H NMR spectrum in DMSO‐d_6_.
**Figure S160:** ^13^C NMR spectrum in DMSO‐d_6_.
**Figure S161:** ^1^H‐^1^H COSY spectrum in DMSO‐d_6_.
**Figure S162:** ^1^H‐^13^C gHSQC spectrum in DMSO‐d_6_.
**Figure S163:** ^1^H‐^13^C gHMBC spectrum in DMSO‐d_6_.
**Figure S164:** ^1^H‐^15^N gHMBC spectrum in DMSO‐d_6_.
**Figure S165:** ^19^F NMR spectrum in DMSO‐d_6_.
**Figure S166:** ^29^Si NMR spectrum in DMSO‐d_6_.
**Figure S167:** HPLC chromatogram.
**Figure S168:** (−) MS‐ESI spectrum.
**Figure S169:** ^1^H NMR spectrum in DMSO‐d_6_.
**Figure S170:** ^13^C NMR spectrum in DMSO‐d_6_.
**Figure S171:** ^1^H‐^1^H COSY spectrum in DMSO‐d_6_.
**Figure S172:** ^1^H‐^13^C gHSQC spectrum in DMSO‐d_6_.
**Figure S173:** ^1^H‐^13^C gHMBC NMR spectrum in DMSO‐d_6_.
**Figure S174:** ^1^H‐^15^N gHMBC spectrum in DMSO‐d_6_.
**Figure S175:** ^19^F NMR spectrum in DMSO‐d_6_.
**Figure S176:** HPLC chromatogram.
**Figure S177:** (−) MS‐ESI spectrum.
**Figure S178:** ^1^H NMR spectrum in DMSO‐d_6_.
**Figure S179:** ^13^C NMR spectrum in DMSO‐d_6_.
**Figure S180:** ^1^H‐^1^H COSY spectrum in DMSO‐d_6_.
**Figure S181:** ^1^H‐^13^C gHSQC spectrum in DMSO‐d_6_.
**Figure S182:** ^1^H‐^13^C gHMBC NMR spectrum in DMSO‐d_6_.
**Figure S183:** ^1^H‐^15^N gHMBC NMR spectrum in DMSO‐d_6_.
**Figure S184:** ^19^F NMR spectrum in DMSO‐d_6_.
**Figure S185:** ^29^Si NMR spectrum in DMSO‐d_6_.
**Figure S186:** HPLC chromatogram.
**Figure S187:** (−) MS‐ESI spectrum.
**Figure S188:** ^1^H NMR spectrum in DMSO‐d_6_.
**Figure S189:** ^13^C NMR spectrum in DMSO‐d_6_.
**Figure S190:** ^1^H‐^1^H COSY spectrum in DMSO‐d_6_.
**Figure S191:** ^1^H‐^13^C gHSQC spectrum in DMSO‐d_6_.
**Figure S192:** ^1^H‐^13^C gHMBC NMR spectrum in DMSO‐d_6_.
**Figure S193:** ^1^H‐^15^N gHMBC spectrum in DMSO‐d_6_.
**Figure S194:** ^19^F NMR spectrum in DMSO‐d_6_.
**Figure S195:** HPLC chromatogram.
**Figure S196:** (−) MS‐ESI spectrum.
**Figure S197:** ^1^H NMR spectrum in DMSO‐d_6_.
**Figure S198:** ^13^C NMR spectrum in DMSO‐d_6_.
**Figure S199:** ^1^H‐^1^H COSY spectrum in DMSO‐d_6_.
**Figure S200:** ^1^H‐^13^C gHSQC spectrum in DMSO‐d_6_.
**Figure S201:** ^1^H‐^13^C gHMBC NMR spectrum in DMSO‐d_6_.
**Figure S202:** ^1^H‐^15^N gHMBC spectrum in DMSO‐d_6_.
**Figure S203:** ^19^F NMR spectrum in DMSO‐d_6_.
**Figure S204:** ^29^Si NMR spectrum in DMSO‐d_6_.
**Figure S205:** HPLC chromatogram.
**Figure S206:** (−) MS‐ESI spectrum.
**Figure S207:** ^1^H NMR spectrum in DMSO‐d_6_.
**Figure S208:** ^13^C NMR spectrum in DMSO‐d_6_.
**Figure S209:** ^1^H‐^1^H COSY spectrum in DMSO‐d_6_.
**Figure S210:** ^1^H‐^13^C gHSQC spectrum in DMSO‐d_6_.
**Figure S211:** ^1^H‐^13^C gHMBC NMR spectrum in DMSO‐d_6_.
**Figure S212:** ^1^H‐^15^N gHSQC spectrum in DMSO‐d_6_.
**Figure S213:** ^1^H‐^15^N gHMBC spectrum in DMSO‐d_6_.
**Figure S214:** ^19^F NMR spectrum in DMSO‐d_6_.
**Figure S215:** HPLC chromatogram.
**Figure S216:** (−) MS‐ESI spectrum.
**Figure S217:** (+) MS‐ESI spectrum.
**Figure S218:** ^1^H NMR spectrum in DMSO‐d_6_.
**Figure S219:** ^13^C NMR spectrum in DMSO‐d_6_.
**Figure S220:** ^1^H‐^1^H COSY spectrum in DMSO‐d_6_.
**Figure S221:** ^1^H‐^13^C gHSQC spectrum in DMSO‐d_6_.
**Figure S222:** ^1^H‐^13^C gHMBC spectrum in DMSO‐d_6_.
**Figure S223:** ^1^H‐^15^N gHSQC spectrum in DMSO‐d_6_.
**Figure S224:** ^1^H‐^15^N gHMBC spectrum in DMSO‐d_6_.
**Figure S225:** ^19^F NMR spectrum in DMSO‐d_6_.
**Figure S226:** ^29^Si NMR spectrum in DMSO‐d_6_.
**Figure S227:** HPLC chromatogram.
**Figure S228:** (−) MS‐ESI spectrum.
**Figure S229:** ^1^H NMR spectrum in DMSO‐d_6_.
**Figure S230:** ^13^C NMR spectrum in DMSO‐d_6_.
**Figure S231:** ^1^H‐^1^H COSY spectrum in DMSO‐d_6_.
**Figure S232:** ^1^H‐^13^C gHSQC spectrum in DMSO‐d_6_.
**Figure S233:** ^1^H‐^13^C gHMBC spectrum in DMSO‐d_6_.
**Figure S234:** ^1^H‐^15^N gHMBC spectrum in DMSO‐d_6_.
**Figure S235:** ^19^F NMR spectrum in DMSO‐d_6_.
**Figure S236:** Physicochemical descriptors of **AddU** (**21a**) and molecule bioavailability radar chart showing drug‐like properties.
**Figure S237:** Physicochemical descriptors of **AZT** (**21b**) and molecule bioavailability radar chart showing drug‐like properties.
**Figure S238:** Physicochemical descriptors of **FAddU** (**21c**) and molecule bioavailability radar chart showing drug‐like properties.
**Figure S239:** Physicochemical descriptors of **28a** and molecule bioavailability radar chart showing drug‐like properties.
**Figure S240:** Physicochemical descriptors of **28b** and molecule bioavailability radar chart showing drug‐like properties.
**Figure S241:** Physicochemical descriptors of **28c** and molecule bioavailability radar chart showing drug‐like properties.
**Figure S242:** Physicochemical descriptors of **27a** and molecule bioavailability radar chart showing drug‐like properties.
**Figure S243:** Physicochemical descriptors of **27b** and molecule bioavailability radar chart showing drug‐like properties.
**Figure S244:** Physicochemical descriptors of **27c** and molecule bioavailability radar chart showing drug‐like properties.
**Figure S245:** Physicochemical descriptors of **Dimer 1** and molecule bioavailability radar chart showing drug‐like properties.
**Figure S246:** Physicochemical descriptors of **Dimer 2** and molecule bioavailability radar chart showing drug‐like properties.
**Figure S247:** Physicochemical descriptors of **Dimer 3** and molecule bioavailability radar chart showing drug‐like properties.
**Figure S248:** Physicochemical descriptors of **Dimer 4** and molecule bioavailability radar chart showing drug‐like properties.
**Figure S249:** Physicochemical descriptors of **Dimer 5** and molecule bioavailability radar chart showing drug‐like properties.
**Figure S250:** Physicochemical descriptors of **Dimer 6** and molecule bioavailability radar chart showing drug‐like properties.
**Figure S251:** Physicochemical descriptors of **Dimer 7** and molecule bioavailability radar chart showing drug‐like properties.
**Figure S252:** Physicochemical descriptors of **Dimer 8** and molecule bioavailability radar chart showing drug‐like properties.
**Figure S253:** Physicochemical descriptors of **Dimer 9** and molecule bioavailability radar chart showing drug‐like properties.
**Figure S254:** Physicochemical descriptors of **Dimer 10** and molecule bioavailability radar chart showing drug‐like properties.
**Figure S255:** Physicochemical descriptors of **Dimer 11** and molecule bioavailability radar chart showing drug‐like properties.
**Figure S256:** Physicochemical descriptors of **Dimer 12** and molecule bioavailability radar chart showing drug‐like properties.
**Figure S257:** Physicochemical descriptors of **Dimer 13** and molecule bioavailability radar chart showing drug‐like properties.
**Figure S258:** Physicochemical descriptors of **Dimer 14** and molecule bioavailability radar chart showing drug‐like properties.
**Figure S259:** Physicochemical descriptors of **Dimer 15** and molecule bioavailability radar chart showing drug‐like properties.
**Figure S260:** Physicochemical descriptors of **Dimer 16** and molecule bioavailability radar chart showing drug‐like properties.
**Figure S261:** Physicochemical descriptors of **Dimer 17** and molecule bioavailability radar chart showing drug‐like properties.
**Figure S262:** Physicochemical descriptors of **Dimer 18** and molecule bioavailability radar chart showing drug‐like properties.

## Data Availability

The data supporting this article have been included as part of the Supporting Information (SI). SI containing HPLC chromatograms, detailed copies of MS‐ESI and NMR spectra, and bioavailability graphs is available online. See DOI: https://doi.org/10.1111/cbdd.70407. Raw data are stored on the corresponding author's server and are available upon request.

## Endnotes

^1^
Preparation of dibromomethyltriphenylphosphonium bromide (Dolhem et al. 2002).
