# Supplementary material for: Triazole‐Linked Pyrimidine Nucleoside Dimers: Design, Synthesis, and Applications in Medicinal and Nucleic Acid Chemistry. Part IV
Source: Chem Biol Drug Des. 2026 Oct 4;108(4):e70407. doi: 10.1111/cbdd.70407 (PMC13635774; doi:10.1111/cbdd.70407)
Supplement: Supplementary file 1 [file CBDD-108-e70407-s001.docx]

Electronic Supplementary Information

***Triazole-linked pyrimidine nucleoside dimers: design, synthesis, and applications
in medicinal and nucleic acid chemistry. Part IV***

Dagmara Baraniak^a,^*, Dariusz Wawrzyniak^a^, Daniel Baranowski^a^

*^a^Institute of Bioorganic Chemistry, Polish Academy of Sciences, Noskowskiego St 12/14,
61-704 Poznań, Poland*

**Table of contents**

[Spectral analysis of compounds	2](#_Toc216712258)

[3'-azido-2',3'-dideoxyuridine (**21a**, AddU)	2](#_Toc216712259)

[3'-azido-thymidine (**21b**, AZT)	3](#_Toc216712260)

[3'-azido-5-fluoro-2',3'-dideoxyuridine (**21c**, AFddU)	4](#_Toc216712261)

[4'-ethynyl-2',5'-dideoxy-5'-noruridine (**28a**)	4](#_Toc216712262)

[4'-ethynyl-5'-deoxy-5'-northymidine (**28b**)	11](#_Toc216712263)

[4'-ethynyl-5-fluoro-2',5'-dideoxy-5'-noruridine (**28c**)	16](#_Toc216712264)

[3'-TBDMS-4'-ethynyl-2',5'-dideoxy-5'-noruridine (**27a**)	19](#_Toc216712265)

[3'-TBDMS-4'-ethynyl-5'-deoxy-5'-northymidine (**27b**)	26](#_Toc216712266)

[3'-TBDMS-4'-ethynyl-5-fluoro-2',5'-dideoxy-5'-noruridine (**27c**)	32](#_Toc216712267)

[Dimer **1**	39](#_Toc216712268)

[Dimer **2**	45](#_Toc216712269)

[Dimer **3**	50](#_Toc216712270)

[Dimer **4**	56](#_Toc216712271)

[Dimer **5**	62](#_Toc216712272)

[Dimer **6**	67](#_Toc216712273)

[Dimer **7**	73](#_Toc216712274)

[Dimer **8**	79](#_Toc216712275)

[Dimer **9**	83](#_Toc216712276)

[Dimer **10**	90](#_Toc216712277)

[Dimer **11**	95](#_Toc216712278)

[Dimer **12**	102](#_Toc216712279)

[Dimer **13**	108](#_Toc216712280)

[Dimer **14**	114](#_Toc216712281)

[Dimer **15**	120](#_Toc216712282)

[Dimer **16**	126](#_Toc216712283)

[Dimer **17**	133](#_Toc216712284)

[Dimer **18**	140](#_Toc216712285)

[Biological evaluation	146](#_Toc216712286)

# Spectral analysis of compounds

## 3'-azido-2',3'-dideoxyuridine (21a, AddU)

Figure S_HPLC chromatogram

run time (min): 45.00, retention time (min): 14.377, purity: 100%

Figure S_^1^H NMR spectrum in DMSO-d_6_

Figure S_^13^C NMR spectrum in DMSO-d_6_

## 3'-azido-thymidine (21b, AZT)

Figure S_HPLC chromatogram

run time (min): 45.00, retention time (min): 15.178, purity: 86.80%

## 3'-azido-5-fluoro-2',3'-dideoxyuridine (21c, AFddU)

Figure S_HPLC chromatogram

run time (min): 45.00, retention time (min): 14.422, purity: 100%

## 4'-ethynyl-2',5'-dideoxy-5'-noruridine (28a)

Figure S_HPLC chromatogram

run time (min): 45,00, retention time (min): 13,508, purity: 90.39%

Figure S_(−) MS-ESI spectrum

Figure S_^1^H NMR spectrum in DMSO-d_6_

Figure S_^13^C NMR spectrum in DMSO-d_6_

Figure S_^1^H-^1^H COSY spectrum in DMSO-d_6_

Figure S_^1^H-^13^C gHSQC spectrum in DMSO-d_6_

Figure S_^1^H-^13^C gHMBC spectrum in DMSO-d_6_

Figure S_^1^H-^15^N gHSQC spectrum in DMSO-d_6_

Figure S_^1^H-^15^N gHMBC spectrum in DMSO-d_6_

## 4'-ethynyl-5'-deoxy-5'-northymidine (28b)

Figure S_HPLC chromatogram

run time (min): 45,00, retention time (min): 14,923, purity: 100%

Figure S_(−) MS-ESI spectrum

Figure S_(+) MS-ESI spectrum

Figure S_^1^H NMR spectrum in DMSO-d_6_

Figure S_^13^C NMR spectrum in DMSO-d_6_

Figure S_^1^H-^1^H COSY spectrum in DMSO-d_6_

Figure S_^1^H-^13^C gHSQC spectrum in DMSO-d_6_

Figure S_^1^H-^13^C gHMBC spectrum in DMSO-d_6_

Figure S_^1^H-^15^N gHMBC spectrum in DMSO-d_6_

## 4'-ethynyl-5-fluoro-2',5'-dideoxy-5'-noruridine (28c)

Figure S_HPLC chromatogram

run time (min): 45,00, retention time (min): 13,953, purity: 100%

Figure S_(−) MS-ESI spectrum

Figure S_(+) MS-ESI spectrum

Figure S_^1^H NMR spectrum in DMSO-d_6_

Figure S_^13^C NMR spectrum in DMSO-d_6_

Figure S_^19^F NMR spectrum in DMSO-d_6_

## 3'-TBDMS-4'-ethynyl-2',5'-dideoxy-5'-noruridine (27a)

Figure S_HPLC chromatogram

run time (min): 45,00, retention time (min): 25,910, purity: 95.15%

Figure S_(−) MS-ESI spectrum

Figure S_(+) MS-ESI spectrum

Figure S_^1^H NMR spectrum in DMSO-d_6_

Figure S_^13^C NMR spectrum in DMSO-d_6_

Figure S_^1^H-^1^H COSY spectrum in DMSO-d_6_

Figure S_^1^H-^13^C gHSQC spectrum in DMSO-d_6_

Figure S_^1^H-^13^C gHMBC spectrum in DMSO-d_6_

Figure S_^1^H-^15^N gHMBC spectrum in DMSO-d_6_

Figure S_^29^Si NMR spectrum in DMSO-d_6_

## 3'-TBDMS-4'-ethynyl-5'-deoxy-5'-northymidine (27b)

Figure S_HPLC chromatogram

run time (min): 45,00, retention time (min): 26,822, purity: 97.41%

Figure S_(−) MS-ESI spectrum

Figure S_(+) MS-ESI spectrum

Figure S_^1^H NMR spectrum in DMSO-d_6_

Figure S_^13^C NMR spectrum in DMSO-d_6_

Figure S_^1^H-^1^H COSY spectrum in DMSO-d_6_

Figure S_^1^H-^13^C gHSQC spectrum in DMSO-d_6_

Figure S_^1^H-^13^C gHMBC spectrum in DMSO-d_6_

Figure S_^1^H-^15^N gHMBC spectrum in DMSO-d_6_

Figure S_^29^Si NMR spectrum in DMSO-d_6_

## 3'-TBDMS-4'-ethynyl-5-fluoro-2',5'-dideoxy-5'-noruridine (27c)

Figure S_HPLC chromatogram

run time (min): 45,00, retention time (min): 26,038, purity: 100%

Figure S_(−) MS-ESI spectrum

Figure S_(+) MS-ESI spectrum

Figure S_^1^H NMR spectrum in DMSO-d_6_

Figure S_^13^C NMR spectrum in DMSO-d_6_

Figure S_^1^H-^1^H COSY spectrum in DMSO-d_6_

Figure S_^1^H-^13^C gHSQC spectrum in DMSO-d_6_

Figure S_^1^H-^13^C gHMBC spectrum in DMSO-d_6_

Figure S_^1^H-^15^N gHSQC spectrum in DMSO-d_6_

Figure S_^1^H-^15^N gHMBC spectrum in DMSO-d_6_

Figure S_^29^Si NMR spectrum in DMSO-d

Figure S_^19^F NMR spectrum in DMSO-d_6_

## Dimer 1

Figure S_HPLC chromatogram

run time (min): 45,00, retention time (min): 21,360, purity: 100%

Figure S_(−) MS-ESI spectrum

Figure S_(+) MS-ESI spectrum

Figure S_^1^H NMR spectrum in DMSO-d_6_

Figure S_^13^C NMR spectrum in DMSO-d_6_

Figure S_^1^H-^1^H COSY spectrum in DMSO-d_6_

Figure S_^1^H-^13^C gHSQC spectrum in DMSO-d_6_

Figure S_^1^H-^13^C gHMBC spectrum in DMSO-d_6_

Figure S_^1^H-^15^N gHSQC spectrum in DMSO-d_6_

Figure S_^1^H-^15^N gHMBC spectrum in DMSO-d_6_

Figure S_^29^Si NMR spectrum in DMSO-d_6_

## Dimer 2

Figure S_HPLC chromatogram

run time (min): 45,00, retention time (min): 13,647, purity: 98.42%

Figure S_(−) MS-ESI spectrum

Figure S_^1^H NMR spectrum in DMSO-d_6_

Figure S_^13^C NMR spectrum in DMSO-d_6_

Figure S_^1^H-^1^H COSY spectrum in DMSO-d_6_

Figure S_^1^H-^13^C gHSQC spectrum in DMSO-d_6_

Figure S_^1^H-^13^C gHMBC spectrum in DMSO-d_6_

Figure S_^1^H-^15^N gHMBC spectrum in DMSO-d_6_

## Dimer 3

Figure S_HPLC chromatogram

run time (min): 45,00, retention time (min): 21,837, purity: 92.92%

Figure S_(−) MS-ESI spectrum

Figure S_^1^H NMR spectrum in DMSO-d_6_

Figure S_^13^C NMR spectrum in DMSO-d_6_

Figure S_^1^H-^1^H COSY spectrum in DMSO-d_6_

Figure S_^1^H-^13^C gHSQC spectrum in DMSO-d_6_

Figure S_^1^H-^13^C gHMBC spectrum in DMSO-d_6_

Figure S_^1^H-^15^N gHSQC spectrum in DMSO-d_6_

Figure S_^1^H-^15^N gHMBC spectrum in DMSO-d_6_

Figure S_^29^Si NMR spectrum in DMSO-d_6_

## Dimer 4

Figure S_HPLC chromatogram

run time (min): 45,00, retention time (min): 14,193, purity: 100%

Figure S_(−) MS-ESI spectrum

Figure S_^1^H NMR spectrum in DMSO-d_6_

Figure S_^13^C NMR spectrum in DMSO-d_6_

Figure S_^1^H-^1^H COSY spectrum in DMSO-d_6_

Figure S_^1^H-^13^C gHSQC in DMSO-d_6_

Figure S_^1^H-^13^C gHMBC spectrum in DMSO-d_6_

Figure S_^1^H-^15^N gHMBC spectrum in DMSO-d_6_

## Dimer 5

Figure S_HPLC chromatogram

run time (min): 45,00, retention time (min): 22,500, purity: 89.52%

Figure S_(−) MS-ESI spectrum

Figure S_^1^H NMR spectrum in DMSO-d_6_

Figure S_^13^C NMR spectrum in DMSO-d_6_

Figure S_^1^H-^1^H COSY spectrum in DMSO-d_6_

Figure S_^1^H-^13^C gHSQC spectrum in DMSO-d_6_

Figure S_^1^H-^13^C gHMBC spectrum in DMSO-d_6_

Figure S_^1^H-^15^N gHMBC spectrum in DMSO-d_6_

Figure S_^19^F NMR spectrum in DMSO-d_6_

Figure S_^29^Si NMR spectrum in DMSO-d_6_

## Dimer 6

Figure S_HPLC chromatogram

run time (min): 45,00, retention time (min): 13,805, purity: 100%

Figure S_(−) MS-ESI spectrum

Figure S_^1^H NMR spectrum in DMSO-d_6_

Figure S_^13^C NMR spectrum in DMSO-d_6_

Figure S_^1^H-^1^H COSY spectrum in DMSO-d_6_

Figure S_^1^H-^13^C gHSQC spectrum in DMSO-d_6_

Figure S_^1^H-^13^C gHMBC spectrum in DMSO-d_6_

Figure S_^1^H-^15^N gHSQC spectrum in DMSO-d_6_

Figure S_^1^H-^15^N gHMBC spectrum in DMSO-d_6_

Figure S_^19^F NMR spectrum in DMSO-d_6_

## Dimer 7

Figure S_HPLC chromatogram

run time (min): 45,00, retention time (min): 21,875, purity: 96.14%

Figure S_(−) MS-ESI spectrum

Figure S_(+) MS-ESI spectrum

Figure S_^1^H NMR spectrum in DMSO-d_6_

Figure S_^13^C NMR spectrum in DMSO-d_6_

Figure S_^1^H-^1^H COSY spectrum in DMSO-d_6_

Figure S_^1^H-^13^C gHSQC in DMSO-d_6_

Figure S_^1^H-^13^C gHMBC spectrum in DMSO-d_6_

Figure S_^1^H-^15^N gHSQC spectrum in DMSO-d_6_

z

Figure S_^1^H-^15^N gHMBC spectrum in DMSO-d_6_

Figure S_^29^Si NMR spectrum in DMSO-d_6_

## Dimer 8

Figure S_HPLC chromatogram

run time (min): 45,00, retention time (min): 14,212, purity: 100%

Figure S_(−) MS-ESI spectrum

Figure S_^1^H NMR spectrum in DMSO-d_6_

Figure S_^13^C NMR spectrum in DMSO-d_6_

Figure S_^1^H-^1^H COSY spectrum in DMSO-d_6_

Figure S_^1^H-^13^C gHSQC in DMSO-d_6_

Figure S_^1^H-^13^C gHMBC spectrum in DMSO-d_6_

Figure S_^1^H-^15^N gHMBC spectrum in DMSO-d_6_

## Dimer 9

Figure S_HPLC chromatogram

run time (min): 45,00, retention time (min): 22,352, purity: 96.04%

Figure S_(−) MS-ESI spectrum

Figure S_(+) MS-ESI spectrum

Figure S_^1^H NMR spectrum in DMSO-d_6_

Figure S_^13^C NMR spectrum in DMSO-d_6_

Figure S_^1^H-^1^H COSY spectrum in DMSO-d_6_

Figure S_^1^H-^13^C gHSQC spectrum in DMSO-d_6_

Figure S_^1^H-^13^C gHMBC spectrum in DMSO-d_6_

Figure S_^1^H-^15^N gHMBC spectrum in DMSO-d_6_

Figure S_^29^Si NMR spectrum in DMSO-d_6_

## Dimer 10

Figure S_HPLC chromatogram

run time (min): 45,00, retention time (min): 14,810, purity: 100%

Figure S_(−) MS-ESI spectrum

Figure S_^1^H NMR spectrum in DMSO-d_6_

Figure S_^13^C NMR spectrum in DMSO-d_6_

Figure S_^1^H-^1^H COSY spectrum in DMSO-d_6_

Figure S_^1^H-^13^C gHSQC spectrum in DMSO-d_6_

Figure S_^1^H-^13^C gHMBC spectrum in DMSO-d_6_

Figure S_^1^H-^15^N gHMBC spectrum in DMSO-d_6_

## Dimer 11

Figure S_HPLC chromatogram

run time (min): 45,00, retention time (min): 21,918, purity: 96.52%

Figure S_(−) MS-ESI spectrum

Figure S_(+) MS-ESI spectrum

Figure S_^1^H NMR spectrum in DMSO-d_6_

Figure S_^13^C NMR spectrum in DMSO-d_6_

Figure S_^1^H-^1^H COSY spectrum in DMSO-d_6_

Figure S_^1^H-^13^C gHSQC spectrum in DMSO-d_6_

Figure S_^1^H-^13^C gHMBC spectrum in DMSO-d_6_

Figure S_^1^H-^15^N gHMBC spectrum in DMSO-d_6_

Figure S_^19^F NMR spectrum in DMSO-d_6_

Figure S_^29^Si NMR spectrum in DMSO-d_6_

## Dimer 12

Figure S_HPLC chromatogram

run time (min): 45,00, retention time (min): 14,412, purity: 97.28%

Figure S_(−) MS-ESI spectrum

Figure S_^1^H NMR spectrum in DMSO-d_6_

Figure S_^13^C NMR spectrum in DMSO-d_6_

Figure S_^1^H-^1^H COSY spectrum in DMSO-d_6_

Figure S_^1^H-^13^C gHSQC spectrum in DMSO-d_6_

Figure S_^1^H-^13^C gHMBC NMR spectrum in DMSO-d_6_

Figure S_^1^H-^15^N gHMBC spectrum in DMSO-d_6_

Figure S_^19^F NMR spectrum in DMSO-d_6_

## Dimer 13

Figure S_HPLC chromatogram

run time (min): 45,00, retention time (min): 21,347, purity: 95.44%

Figure S_(−) MS-ESI spectrum

Figure S_^1^H NMR spectrum in DMSO-d_6_

Figure S_^13^C NMR spectrum in DMSO-d_6_

Figure S_^1^H-^1^H COSY spectrum in DMSO-d_6_

Figure S_^1^H-^13^C gHSQC spectrum in DMSO-d_6_

Figure S_^1^H-^13^C gHMBC NMR spectrum in DMSO-d_6_

Figure S_^1^H-^15^N gHMBC NMR spectrum in DMSO-d_6_

Figure S_^19^F NMR spectrum in DMSO-d_6_

Figure S_^29^Si NMR spectrum in DMSO-d_6_

## Dimer 14

Figure S_HPLC chromatogram

run time (min): 45,00, retention time (min): 13,683, purity: 100%

Figure S_(−) MS-ESI spectrum

Figure S_^1^H NMR spectrum in DMSO-d_6_

Figure S_^13^C NMR spectrum in DMSO-d_6_

Figure S_^1^H-^1^H COSY spectrum in DMSO-d_6_

Figure S ^1^H-^13^C gHSQC spectrum in DMSO-d_6_

Figure S_^1^H-^13^C gHMBC NMR spectrum in DMSO-d_6_

Figure S_^1^H-^15^N gHMBC spectrum in DMSO-d_6_

Figure S_^19^F NMR spectrum in DMSO-d_6_

## Dimer 15

Figure S_HPLC chromatogram

run time (min): 45,00, retention time (min): 21,812, purity: 94.75%

Figure S_(−) MS-ESI spectrum

Figure S_^1^H NMR spectrum in DMSO-d_6_

Figure S_^13^C NMR spectrum in DMSO-d_6_

Figure S_^1^H-^1^H COSY spectrum in DMSO-d_6_

Figure S_^1^H-^13^C gHSQC spectrum in DMSO-d_6_

Figure S_^1^H-^13^C gHMBC NMR spectrum in DMSO-d_6_

Figure S_^1^H-^15^N gHMBC spectrum in DMSO-d_6_

Figure S_^19^F NMR spectrum in DMSO-d_6_

Figure S_^29^Si NMR spectrum in DMSO-d_6_

## Dimer 16

Figure S_HPLC chromatogram

run time (min): 45,00, retention time (min): 14,270, purity: 83.96%

Figure S_(−) MS-ESI spectrum

Figure S_^1^H NMR spectrum in DMSO-d_6_

Figure S_^13^C NMR spectrum in DMSO-d_6_

Figure S_^1^H-^1^H COSY spectrum in DMSO-d_6_

Figure S_^1^H-^13^C gHSQC spectrum in DMSO-d_6_

Figure S_^1^H-^13^C gHMBC NMR spectrum in DMSO-d_6_

Figure S_^1^H-^15^N gHSQC spectrum in DMSO-d_6_

Figure S_^1^H-^15^N gHMBC spectrum in DMSO-d_6_

Figure S_^19^F NMR spectrum in DMSO-d_6_

## Dimer 17

Figure S_HPLC chromatogram

run time (min): 45,00, retention time (min): 21,460, purity: 92.69%

Figure S_(−) MS-ESI spectrum

Figure S (+) MS-ESI spectrum

Figure S_^1^H NMR spectrum in DMSO-d_6_

Figure S_^13^C NMR spectrum in DMSO-d_6_

Figure S_^1^H-^1^H COSY spectrum in DMSO-d_6_

Figure S_^1^H-^13^C gHSQC spectrum in DMSO-d_6_

Figure S_^1^H-^13^C gHMBC spectrum in DMSO-d_6_

Figure S_^1^H-^15^N gHSQC spectrum in DMSO-d_6_

Figure S_^1^H-^15^N gHMBC spectrum in DMSO-d_6_

Figure S_^19^F NMR spectrum in DMSO-d_6_

Figure S_^29^Si NMR spectrum in DMSO-d_6_

## Dimer 18

Figure S_HPLC chromatogram

run time (min): 45,00, retention time (min): 13,978, purity: 100%

Figure S_(−) MS-ESI spectrum

Figure S_^1^H NMR spectrum in DMSO-d_6_

Figure S_^13^C NMR spectrum in DMSO-d_6_

Figure S_^1^H-^1^H COSY spectrum in DMSO-d_6_

Figure S_^1^H-^13^C gHSQC spectrum in DMSO-d_6_

Figure S_^1^H-^13^C gHMBC spectrum in DMSO-d_6_

Figure S_^1^H-^15^N gHMBC spectrum in DMSO-d_6_

Figure S_^19^F NMR spectrum in DMSO-d_6_

# Biological evaluation

**Data related to biological research**

Legend for all bioavailability radar charts:

The pink color represents the optimal range for each property. LIPO – lipophilicity (XLOGP3 range between 0.7 and 5.0); SIZE – molecular weight (MW range between 150 and 500 g/mol); POLAR – polarity (TPSA range between 20 and 130 Å); INSOLU – solubility (LogS value not greater than 6); INSATU – saturation (fraction of C atoms in sp^3^ hybridization not less than 0.25); FLEX – flexibility (number of rotating bonds not more than 9).

Figure S_Physicochemical descriptors of **AddU** (**21a**) and molecule bioavailability radar chart showing drug-like properties

Figure S_Physicochemical descriptors of **AZT** (**21b**) and molecule bioavailability radar chart showing drug-like properties

Figure S_Physicochemical descriptors of **FAddU** (**21c**) and molecule bioavailability radar chart showing drug-like properties

Figure S_Physicochemical descriptors of **28a** and molecule bioavailability radar chart showing drug-like properties

Figure S_Physicochemical descriptors of **28b** and molecule bioavailability radar chart showing drug-like properties

Figure S_Physicochemical descriptors of **28c** and molecule bioavailability radar chart showing drug-like properties

Figure S_Physicochemical descriptors of **27a** and molecule bioavailability radar chart showing drug-like properties

Figure S_Physicochemical descriptors of **27b** and molecule bioavailability radar chart showing drug-like properties

Figure S_Physicochemical descriptors of **27c** and molecule bioavailability radar chart showing drug-like properties

Figure S_Physicochemical descriptors of **Dimer 1** and molecule bioavailability radar chart showing drug-like properties

Figure S_Physicochemical descriptors of **Dimer 2** and molecule bioavailability radar chart showing drug-like properties

Figure S_Physicochemical descriptors of **Dimer 3** and molecule bioavailability radar chart showing drug-like properties

Figure S_Physicochemical descriptors of **Dimer 4** and molecule bioavailability radar chart showing drug-like properties

Figure S_Physicochemical descriptors of **Dimer 5** and molecule bioavailability radar chart showing drug-like properties

Figure S_Physicochemical descriptors of **Dimer 6** and molecule bioavailability radar chart showing drug-like properties

Figure S_Physicochemical descriptors of **Dimer 7** and molecule bioavailability radar chart showing drug-like properties

Figure S_Physicochemical descriptors of **Dimer 8** and molecule bioavailability radar chart showing drug-like properties

Figure S_Physicochemical descriptors of **Dimer 9** and molecule bioavailability radar chart showing drug-like properties

Figure S_Physicochemical descriptors of **Dimer 10** and molecule bioavailability radar chart showing drug-like properties

Figure S_Physicochemical descriptors of **Dimer 11** and molecule bioavailability radar chart showing drug-like properties

Figure S_Physicochemical descriptors of **Dimer 12** and molecule bioavailability radar chart showing drug-like properties

Figure S_Physicochemical descriptors of **Dimer 13** and molecule bioavailability radar chart showing drug-like properties

Figure S_Physicochemical descriptors of **Dimer 14** and molecule bioavailability radar chart showing drug-like properties

Figure S_Physicochemical descriptors of **Dimer 15** and molecule bioavailability radar chart showing drug-like properties

Figure S_Physicochemical descriptors of **Dimer 16** and molecule bioavailability radar chart showing drug-like properties

Figure S_Physicochemical descriptors of **Dimer 17** and molecule bioavailability radar chart showing drug-like properties

Figure S_Physicochemical descriptors of **Dimer 18** and molecule bioavailability radar chart showing drug-like properties
